# Improving Children’s Diets by Introducing Fruits and Vegetables in Group-Based Settings: A Scoping Review

**DOI:** 10.1093/nutrit/nuaf092

**Published:** 2025-07-03

**Authors:** Abigail Pickard, Emma Alving-Jessep, Christopher Delivett, Rosemary H Jenkins, Luke Pullar, Claire Farrow, Jacqueline Blissett

**Affiliations:** School of Psychology and Institute of Health and Neurodevelopment, Aston University, Birmingham B4 7ET, United Kingdom; School of Health in Social Science, University of Edinburgh, Edinburgh EH8 9AG, United Kingdom; School of Psychology and Institute of Health and Neurodevelopment, Aston University, Birmingham B4 7ET, United Kingdom; Institute of Applied Health Research, University of Birmingham, Birmingham B15 2TT, United Kingdom; School of Psychology and Institute of Health and Neurodevelopment, Aston University, Birmingham B4 7ET, United Kingdom; Food System Team, Public Health Division, Birmingham City Council, Birmingham B7 4BL, United Kingdom; School of Psychology and Institute of Health and Neurodevelopment, Aston University, Birmingham B4 7ET, United Kingdom; School of Exercise and Nutrition Sciences, Deakin University, Geelong, Victoria VIC 3220, Australia; School of Psychology and Institute of Health and Neurodevelopment, Aston University, Birmingham B4 7ET, United Kingdom; School of Psychology and Institute of Health and Neurodevelopment, Aston University, Birmingham B4 7ET, United Kingdom

**Keywords:** preschool children, diet, educational settings, scoping review

## Abstract

**Context:**

In 2022, less than 18% of UK children aged 5-7 years consumed 5 portions of fruit and vegetables, with an average intake of 3 portions per day. Group settings (eg, schools or nurseries) present an opportunity to apply policies to encourage children’s consumption of novel and healthy foods.

**Objective:**

The extent and types of evidence regarding efforts to increase the consumption of fruits and vegetables by young children in group settings who reside in high-income countries was investigated in this scoping review.

**Data Sources:**

A systematic scoping review was conducted that followed the JBI guidelines and included articles, published from 2012 onward, about methods to increase consumption of novel and/or healthy foods by children aged 3-7 years in settings within local government control and who were residing in high-income countries.

**Data Extraction:**

A total of 7000 articles were initially identified, of which 114 were included in this review after screening.

**Data Analysis:**

Intervention methods included an array of different techniques from educational programs to repeated exposure, food modification, and behavioral modeling approaches.

**Conclusion:**

Interventions administered to children aged between 3 and 7 years in group-based settings have been shown to improve the consumption of healthy foods, specifically fruits and vegetables. Behavioral modeling and sensory play interventions, in particular, present the highest level of success.

## INTRODUCTION

A healthy and balanced diet has long been acknowledged as essential for optimal developmental trajectories.^1–4^ This is globally promoted by the World Health Organization, which supports the consumption of health-beneficial fruits, vegetables, and fibers, from the very start of solid food consumption.^5^ Following these recommendations has been linked to physical, cognitive, and behavioral benefits throughout life.^6–8^ Childhood is a key period for establishing healthy eating habits and fostering a balanced and varied diet.^9^

The UK government advises consuming 5 portions of fruits and vegetables (FV) each day.^10^ However, in 2022, less than 18% of UK children aged 5-7 years consumed 5 portions of FV; the average intake was 3 portions per day.^11^ Additionally, lower socioeconomic status and limited availability or accessibility of vegetables at home are associated with reduced intake.^12^ Community-based approaches are thus ideal in that they have a universal approach, reaching children from all backgrounds to increase FV consumption by young children regardless of the socioeconomic setting. Furthermore, group settings that are controlled by local government, such as schools, provide the opportunity to apply and execute food policy in a way that reduces inequity in access to high-quality food and better nutrition.

Several studies have shown the effectiveness of group settings and school-based interventions in promoting health behaviors, such as increased physical activity in young children.^13–16^ Thus, group settings are promising places for children to learn to engage with new and healthy food. But to make actionable recommendations on how best to achieve this, we need to collate the relevant evidence to determine the effectiveness of what works in these contexts.

A systematic review by Evans et al,^17^ published in 2012, examined school-based interventions to improve FV intake by children 5-12 years old. Therefore, our objective for this scoping review was to map the existing literature regarding evidence aiming to increase the consumption of novel and/or healthy foods by children aged 3-7 years, within group settings, in high-income countries, from 2012 onward. By setting 2012 as the starting point, we aimed to build on the findings of Evans et al^17^ while focusing on more recent research that might reflect evolving methodologies, updated intervention strategies, and newer health guidelines. This approach allowed us to avoid duplicating previous work and to concentrate on the latest developments in this field.

Given the volume of systematic reviews,^18^ a scoping review, which aims to map the existing literature on a particular research area to identify key concepts, gaps, types of evidence, and the overall scope of the research, was considered fundamental to guide researchers and policymakers. This specific age range was selected because early childhood (ages 3-7 years) is a crucial stage for establishing lifelong dietary habits. Children in this age range are forming food preferences and are highly receptive to dietary interventions. Additionally, this age group is often targeted in early education settings, where structured programs can effectively support healthy eating behaviors.

## METHODS

The proposed scoping review was conducted per the JBI methodology for scoping reviews, which provides guidelines on methodological issues, such as when a scoping review is (or is not) appropriate, and how to extract, analyze, and present results. The JBI guidelines also provide clarification for implications for practice and research.^19^ A preliminary search of Web of Science and PROSPERO’s international prospective register of systematic reviews was conducted, and no current or underway systematic or scoping reviews on the topic were identified.

### Search Strategy

The search strategy aimed to locate both qualitative and quantitative sources of evidence pertaining to the review question. An initial limited search of Web of Science was undertaken to identify articles on the topic. The topic terms contained in the titles and abstracts of relevant articles, and the index terms used to describe the articles were used to develop a full search strategy (Table S1). The search strategy, including all identified keywords and index terms, was adapted for each included database and/or information source. Searched databases included Web of Science, Scopus, PsycINFO (via ProQuest platform), and MEDLINE. The exploration of grey literature involved hand-searching reference lists for additional applicable sources. Searches were conducted in February 2024.

### Study Selection

Inclusion criteria were kept broad deliberately to fully encapsulate the literature as best as possible. Sources of evidence, therefore, included scientific articles published in journals, as well as literature reviews and meta-analyses, conference papers and proceedings, and doctoral theses. Quantitative and qualitative studies were considered, provided they examined, through experimental or interventional design, children’s consumption of novel or “healthy” foods. Searches were limited to the English language for practical reasons. Only studies published since 2012 were included because the similar 2012 review by Evans et al^17^ examined school-based interventions to improve FV intake by children aged 5-12 years.^17^ Studies involving parents were included in this review if they took place within group settings controlled by the local government.

Table 1 presents the study eligibility criteria, which fall into 4 categories: participants, concept, context, and types of sources. All identified citations were collated and uploaded into Excel, and duplicates were removed. After a pilot test, titles and abstracts were then screened by 3 independent reviewers (A.P., C.D., and L.P.) for assessment against the inclusion criteria for the review. Each of those reviewers received an equal number of articles to screen, in addition to a subset of articles (∼10%) from the other reviewers to check for inter-rater reliability. Potentially relevant sources were retrieved in full, and their citation details were imported. The full texts of selected citations were assessed in detail against the inclusion criteria by the same 3 independent reviewers. Reasons for excluding sources of evidence that did not meet the inclusion criteria were recorded and are reported in the scoping review. Any disagreements that arose between the reviewers at each stage of the selection process were resolved through discussion.

**Table 1.** Study Eligibility Criteria for the Inclusion and Exclusion of Articles

Parameter	Criteria
Participants	Children between the ages of 3 and 7 years with no known clinical condition
	Exclude evidence looking at children before the age of 3 years or after the age of 7 years, unless the mean age of the sample at baseline falls within 3-7 years old. Age range criteria based upon 'early years foundation stage' (ages 3-5 years) and 'key stage 1' (ages 5-7 years) in UK children’s school settings.
	Exclude evidence examining children with a clinical condition (eg, avoidant/restrictive food intake disorder)
	Exclude evidence that solely focuses on obesity as the outcome variable
Concept	Interventions and/or studies looking to increase consumption of novel and/or healthy foods, within group settings controlled by local government (eg, childcare and education settings)
	Searches will be limited to evidence written in the English language, for practical reasons.
	Searches will be limited to evidence published during or after 2012 in recognition of a similar review conducted by Evans et al.^17^
	Exclude evidence without measures of actual consumption (eg, studies measuring indirect outcomes such as food preference) Exclude evidence from privately owned initiatives and/or organizations that operate outside local government control (eg, The Scout Association)
Context	Interventions and/or studies investigating children residing in high-income countries
	Exclude evidence on children residing in low- and middle-income countries, based on the Organisation for Economic Co-operation and Development register
Types of sources	Sources of evidence may include scientific articles published in journals, literature reviews, meta-analyses, conference papers and proceedings, as well as doctoral theses. Both quantitative and qualitative studies will be considered, provided they examine children’s consumption of novel or healthy foods.

### Synthesizing the Data

Data were extracted from eligible papers by 3 independent reviewers (A.P., E.A., and L.P.) using a data extraction tool developed by the reviewers (see https://osf.io/hfzgy for Supplementary Materials). The data extracted included specific details about the participants, concept, context, study methods, and key findings relevant to the review question. A draft extraction form is provided in Table S2. To refine the data for the extraction form, 2 reviewers (A.P. and E.A.) evaluated the same 10 articles using the proposed form, as did another combination of reviewers (A.P. and L.P.). for another 25 articles, and they discussed any disagreements. After the discussion, the draft extraction tool was modified to include a section for other findings as well as the originally included key findings. After data extraction, the results were organized in tabular format, outlining the intervention and/or study methodology, factors associated with the consumption of novel and healthy foods, and other key findings relating to the review question.

## RESULTS

**Figure 1.**
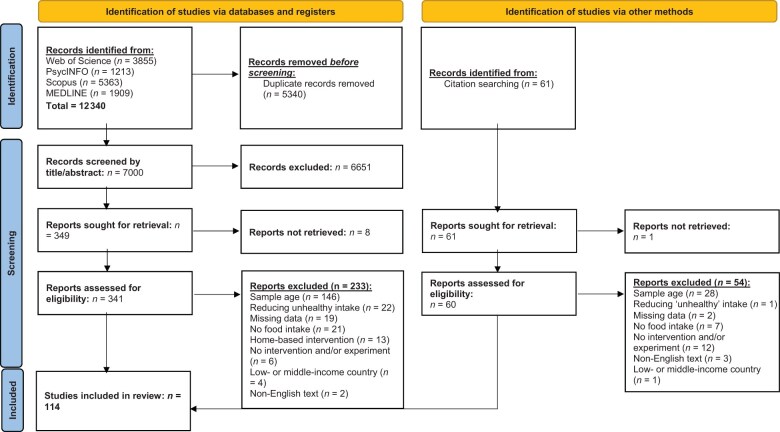
PRISMA Flowchart of the Number and Sources of Articles Included in this Review^20^

A total of 12 340 articles were identified during the search. Duplicate articles (*n* = 5340) were removed, and 7000 articles were screened by title and abstract. After the initial screening, 6651 articles did not meet the eligibility criteria and were removed. Full-text screening was carried out on the 341 articles that were accessible, of which 233 were excluded because they did not meet the eligibility criteria. A total of 108 articles were included in the review from this screening process. Additionally, 61 articles were identified from citations and sought for retrieval. It was not possible to retrieve 1 of these articles and that article was removed from the review. After the screening, a total of 6 articles were brought forward to be included in the scoping review, which took the final total number of articles up to 114, as shown in Figure 1.^20^

The predominant reason for exclusion was the age of the participants in the study. On the basis of the strict age range of children aged between 3 and 7 years, 174 papers were excluded. A total of 18 papers either did not include an intervention or experiment, 28 had no measure of food intake, 23 investigated solely the reduction of intake of “unhealthy” food, 13 were home-based interventions rather than preschool settings, and 5 were non-English texts.

### Study Characteristics

The 114 studies included in this review varied widely in study location. The largest proportion were conducted in the United States (*n* = 50; 44%), followed by the United Kingdom (*n* = 18; 15%); the remainder of the articles reported on studies predominantly from throughout Europe, Japan, Canada, South Korea, and Australia. An overview of the study characteristics is presented in Tables S3 and S4. Study populations varied greatly from as few as 6 children^21^ to nearly 5000 participants,^22^ which also reflects the wide variety of research designs used. A total of 6 studies targeted low-income families; however, the majority of studies were carried out with families who had higher-than-average income and education for their respective locations. Predominantly, the children in the studies were aged between 3 and 5 years and were in kindergarten, nursery, or preschool settings. One study extended recruitment to children up to age 10 years^23^; however, findings were stratified and are reported here for the relevant age group only.

All studies in this review included an outcome measure of consumption of fruit and/or vegetables by children. Several included weighted calorific or energy consumption during snacking or mealtimes, as well as parent reports of food consumption. Additionally, several studies measured anthropometric data, including weight, height, body mass index (BMI) and BMI z-score, as well as measures of child liking of the foods included in the studies, child food neophobia, food fussiness, and child eating behavior.

### Intervention Methodology

Several methods were used in the reviewed studies to increase the quantity of healthy foods consumed in school settings. These ranged in duration and structure, with a large amount of difference in success (Table S5 provides a full description of each methodology).

**Figure 2.**
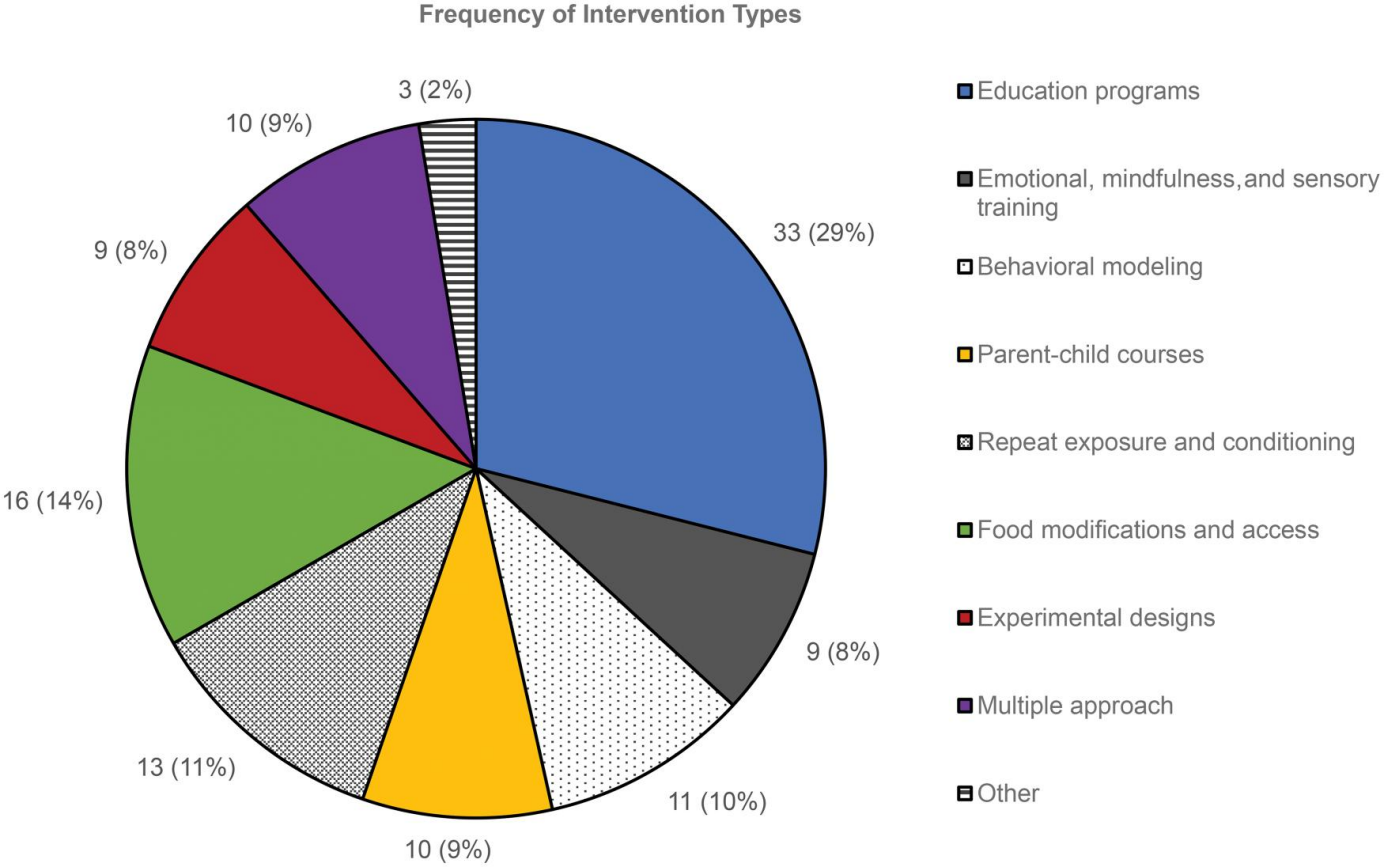
Frequency of Intervention Types

After the synthesis of data, the following categories were determined and used to discuss the interventions (Table S3): (1) education programs; (2) emotional, mindfulness, and sensory training; (3) behavioral modelling using fictional characters through media such as television, books, or toys; (4) parent-child courses; (5) repeat exposure and conditioning; (6) food modification and food access; and (7) experimental design. Three of the studies were not categorized within the categories discussed, and 10 of the studies used multiple approaches within their interventions. Figure 2 provides a visualization of the distribution of intervention types.

### Educational Program Interventions

A total of 33 studies included interventions that were designed in the form of educational programs. The interventions ranged in length from a single day^24^ to 2 years^25^of follow-up. Of the 33 studies, 22 were successful at eliciting significant increases in FV consumption (Table S3). Some studies reported time-limited effects; for example, Kornilaki et al^26^ observed significantly greater consumption of FV in the intervention group compared with the control group, but this effect was absent at the 3-month follow-up. Two of the studies that found nonsignificant effects of the intervention showed an interesting, and unexpected, effect: an increase in the selection of vegetables despite no increased consumption.^27^^,^^28^ Leis et al^27^ reported consequent significant increases in FV waste.

Studies in which a significant increase was found in the consumption of FV or novel foods also reported a large variation in the amount of success achieved. A 12-week educational intervention plan aimed at increasing young children’s theoretical understanding of nutrition^29^ saw an increase in the consumption of vegetables from an average of 3.8 pieces before the intervention to 9.1 pieces after, in the intervention group only. Consumption by the control group did not significantly increase; however, their baseline consumption was higher, at 6.9 pieces, and remained stable. Interestingly, the number of unique foods consumed was also measured for this intervention, and despite food knowledge significantly increasing in the intervention group, there were no significant differences in the number of unique foods consumed between groups. Baseline discrepancies were also noted in 2 other studies, with significantly more healthy foods consumed by the intervention group than by the control group,^26^^,^^30^ which may have further influenced the postintervention findings.

Kristiansen et al^31^ also noted the impact the education provider’s opinion of the intervention also had on the amount of vegetables consumed. Surprisingly, in schools where staff reported that supplementary materials were perceived as useful “to some degree,” a significantly smaller increase in children’s vegetable intake was observed compared with those whose staff reported perceived usefulness to be “not at all/to a small degree.”

Overall, the inconsistent and mixed effectiveness of these educational programs may be due to the variety of educational approaches and synergistic effects of multiple intervention components. Furthermore, the influence of those delivering the program further impedes effectiveness within educational settings, suggesting it may be necessary for staff to have additional training to facilitate effectiveness.

### Behavioral Modelling Interventions

A total of 11 studies used a behavioral modelling approach in their intervention to encourage healthy food consumption. Multiple methods were used, such as puppets, books, TV programs, including advertisements, and facial displays of emotion modelled by adults. Of the 11 interventions, only 1 study found no significant increase in consumption.^32^ This study was implemented for 12 months and was designed so that staff provided modelling of physically active play and healthy eating every day, along with prompting the children and verbally encouraging them.

Of the studies that showed significant effects, 4 presented interventions based on TV programs in which the characters ranged from peer representation to identifiable cartoon characters such as “Shaun the Sheep.”^33–36^ Each of these focused on the health benefits that their “hero” experienced as a result of eating vegetables. All those children who watched the program ate more vegetables than did children who were not exposed to the modelling behavior. Staiano et al^34^ found that after watching their peers interacting with and eating green bell peppers, the interaction group ate, on average, 15.5 g more of the same vegetable than did the control group. These modelling programs also include positive messages about vegetables, including that they make you “super-fast and strong,” as illustrated in a Dutch study where two TV idols, Ernst and Bobbie, are enthusiastic about vegetables, especially carrots.^36^ This study found significant increases in the consumption of carrots that persisted for 9 months after the intervention. Similar findings can be seen when the modelling behavior is communicated through story time and books.^37–39^ For example, de Droog et al^37^ found that interactive shared reading with children in the educational setting has a positive influence on the consumption of carrots.

One behavioral modelling study investigated the specific element of modelling that influences the consumption of FV in children. Edwards et al^40^ investigated the influence of facial displays of emotion on children’s acceptance and intake of raw broccoli, which is both a commonly disliked vegetable as well as an unfamiliar way of consuming the vegetable for most children. They found that in comparison with a control group, positive facial displays of emotion were associated with greater frequency of tasting of the raw broccoli as well as increased consumption. This finding suggests that witnessing others experience positive responses to eating novel or disliked vegetables may, in turn, positively increase children’s tasting and consumption of the same vegetable. This strategy is easy to implement and could potentially be a method that could be used in educational settings.

Overall, the findings of the modelling interventions present compelling evidence that these types of interventions can successfully influence healthy food consumption. However, most of these studies were of short duration, which leaves a gap in understanding the long-term efficacy of these types of intervention. Further research is needed to determine the extent to which these modelling behaviors influence children’s eating habits and whether it is necessary for substantial repetition to be implemented.

### Parent-Child Interventions

Closely linked to the behavioral modelling interventions are those specifically focused on parent and child interactions. This process often uses a strategy of behavioral modelling, improving parents’ eating behavior to influence the child. However, this differs slightly from behavioral modeling interventions because it also uses elements of behavioral change due to cognitive dissonance, an approach that has been found to be effective in adults to change their behavior for the better.^41^ In these situations, cognitive dissonance is created when a parent is placed in a situation where they might not want to try healthy behavior, such as a new food, or increasing vegetable consumption; however, they do so because they are also asking their own child to do it. This can lead to a positive motivational change in their own behavior to set a good example of modelling the improved behavior for their child. Additionally, the parent-child courses also aim to improve parental education to encourage a longer-term change, because the parents can maintain the child’s education beyond the intervention timeline.

In total, 10 studies included in this review involved interventions designed for both parent and child, which took place in a group setting. The designs of the parent-child interventions ranged from cooking together, family snack time, tasting sessions, healthy lunches, a cooperative giving away food, and educational programs. Of the 10 studies, 3 saw no significant improvement in the consumption of healthy food in the educational setting.^42–44^ Crespo et al^43^ used a multimodal approach to influence family eating at home as well as in educational settings, and although there was a significant increase in the daily consumption of FV in the family-only branch of this study, there was no significant increase in the school settings. Similarly, Willis et al^44^ reported a significant increase in the consumption of cooked vegetables, fresh fruit and baked beans, lentils, or chickpeas.

The variation of methods used in the remaining 7 studies makes it difficult to determine whether it is the influence of parental modelling that increased children’s FV or healthy food consumption, or alternative factors, including increased access to FV (eg, provided by the cooperative approach of the “Bright Bites” Intervention).^45^^,^^46^ Replication and extension of these investigations to delineate these effects are necessary. However, in 1 study, the effect of the intervention persisted for 6 months, and in another study, the effect lasted 1 year.^47^^,^^48^ Both studies used a parental educational approach to increase consumption of health foods, as well as child homework sessions and lessons; however, the intervention of Nyberg et al^48^ only found persisting significant effects in boys. Nonetheless, this approach may have some potential to create a long-term impact on children’s healthy food intake despite uncertainty about the mechanism of effect.

### Emotional, Mindfulness, and Sensory Interventions

Emotional, mindfulness, and sensory interventions were used in 9 studies, all of which reported significant increases in “healthy” food choices or FV consumption. For example, Youssef et al^49^ witnessed significant increases in the consumption of individual target foods; the experimental group consumed more pepper omelets than did the control group, with a medium effect size. Approaches used included mindfulness (*n* = 1), incentivizing (*n* = 1), and acceptance and commitment therapy (*n* = 1), and a large majority (*n* = 6) included sensory stimulation or play. Both the mindfulness^50^ and acceptance and commitment therapy^21^ studies used multisensory techniques to increase consumption of target foods through increasing self-awareness of the experience. All the children in these studies were guided to increase their experience with food through increased experience of smell, tactile play, and tasting. One study^51^ accompanied the sensory play with a rendition of the *Very Hungry Caterpillar*,^52^ a well-known children’s book about a caterpillar exploring FV. The researchers encouraged children to use FV to make a picture on a paper plate and, while doing so, to touch, squeeze, smell, and shape the vegetables necessary to make the picture. In the sensory-play intervention groups, there was a significant increase in the number of food tries, compared with a nonfood exposure group. From these results, increasing children’s sensory experiences of food through guided or mindful methods appears to result in their increased consumption and willingness to try healthy foods such as FV.

### Repeated Exposure and Conditioning Interventions

There were several different nuances to take consider when examining repeated-exposure interventions, because of variations in design and target foods, from repeated exposure to novel foods, commonly disliked vegetables, and familiar foods, among others. In total, 13 articles reported on studies that used this approach to increase consumption of healthy food and/or FV. Ahern et al^53^ found that after a repeated-exposure intervention, children consumed more single-vegetable snacks than mixed-vegetable snacks. These target vegetables included baby corn, red pepper, and celery, and were previously identified as foods that were familiar to the children. However, repeated exposure was not always effective in isolation: children’s consumption of raw broccoli did not increase through repeated exposure, but consumption of the vegetable was increased when concomitantly provided with a familiar palatable sauce, particularly by children with a sensitivity to bitter tastes.^54^

Nonetheless, of the 13 studies that used repeated-exposure methods to increase consumption, most saw some degree of success; however, some only showed moderate success that did not persist over time (Table 1). In 3 studies, an increase in consumption of target vegetables was observed in control groups, which persisted up to 1 year after the intervention.^55–57^ This effect may have been due to the type of control group used; for example, 2 of these studies,^56^^,^^57^ were from the same cohort in Copenhagen and explored increasing the consumption of a food novel to them: daikon. The control group was presented with daikon prepared in the same form, and the intervention group was presented with multiple preparations of daikon. Perhaps unsurprisingly, consumption of daikon increased at the 3-month follow-up for both groups, suggesting that mere repetition of exposure to the vegetable at the moment of testing was enough to increase consumption rather than the planned intervention to expose children to different preparations of a novel food.

### Food Modification Interventions

Several interventions used the methodology of modifying food, predominantly vegetables, to be more palatable for children. This took place in the forms of portion-size control; modification of food by altering the taste profiles of the food; visual alterations, such as cutting methods and shape alteration; as well as cooking methods (eg, raw vs cooked). In total, 16 studies investigated the impact of food modification on the consumption of healthy foods, FV, and novel foods. Mathias et al^58^ manipulated the portion sizes of 1 fruit (canned peaches in light syrup) and 1 vegetable (cooked broccoli). They found that children consumed 41 g (70%) more fruit in the large-portion conditions compared with the reference conditions. Similarly, in the large-portion conditions with larger side dishes of broccoli, children ate an average of 12 g (37%) more. However, not all studies showed a positive portion size effect: in 1 study, overall portion size was a nonsignificant predictor of vegetable consumption.^59^ Nonetheless, of the 16 studies with interventions that used food modification approaches, 10 found significant increases in consumption. A Slovenian study modified kindergarten menus to meet recommended food intake (RDFI) guidelines and found that children in the intervention group consumed significantly greater amounts of refined foods and potatoes (57% vs 103% of the RDFI) and fruit (56% vs 67% RDFI) than the control group.^60^ However, this significant increase was only observed in the food intake data from kindergarten and not the intake data reported by parents.

In a study on the effect of food presentation, Correia et al^61^ found increases in broccoli consumption, but only when this was served on a pizza as opposed to being served alone. Interestingly, pizza consumption decreased by 14.3 g when topped with broccoli. The use of dip to increase the consumption of broccoli was only significant in children who had a genetic sensitivity to bitter tastes.^54^ For fruits in particular, 1 study found that dishware influenced the amount consumed, with children eating larger amounts from adult-sized plates.^62^ However, this did not translate to vegetables; only vegetable liking influenced the amount eaten in this study. The wide variety of methods used can substantially affect the success of this intervention type and make it difficult to summarize the effectiveness of this approach. More research is needed to ascertain whether food modification is the best option for designing interventions for group settings, and if so, what type of modification is most effective.

### Multiple Approach Interventions

In total, 10 studies included in this review used multiple interventions as an approach to increasing FV or healthy food consumption. As expected from this category, the designs of the study varied widely; however, only 4 of these 10 studies were successful in their aims. Nekitsing et al^63^ used behavioral modeling, as well as sensory play, to increase the consumption of carrots or celeriac. In every condition, the children were read stories and were encouraged to play with 1 of the 2 vegetables. Given that both sensory play and behavioral modeling substantially influenced the children’s consumption of vegetables, it cannot be ascertained whether there was a larger influence of 1 type of intervention than the other, or whether they had an additive effect.

Four studies included teacher, classroom, and parental components, involving education, modeling, and play to increase children’s FV or other healthy food consumption.^64–67^ Bell et al^67^ manipulated food provision, mealtime environment, and curriculum to increase vegetable intake by children aged 2-5 years, but there were no statistically significant differences between any of the intervention groups and the control group for vegetable intake. The SuperFIT program consisted of a preschool component and a family component to promote healthy dietary behaviors based on systems theories and socioecological frameworks.^68^ Although the preliminary findings seem promising in terms of increasing vegetable intake, the authors note that the sample size was too small to detect any significant effects.^68^ Finally, Farrow et al^69^ used a multiple-method approach combining repeated exposure, modeling, and food reward through the use of a vegetable-inspired math game available on mobile devices. This game uses pictures of real vegetables as characters in a child’s mathematical learning environment. The researchers found increased consumption of vegetables by children who played the vegetable math game compared with a control app, showing that repeated exposure to a visual image of a real vegetable has the potential to influence intake.

### Experimental Designs

Nine studies used experimental designs rather than interventions to investigate the influence of various manipulations on the consumption of FV (Table 1). Four of these studies investigated the impact of child choice on the consumption of FV.^70–73^ Two studies found that when children were given no choice, they are significantly fewer vegetables,^70^^,^^73^ whereas Olsen et al^72^ found that for carrots specifically, there was an increase in consumption when there was no other choice. This finding was not replicated for sugar snap peas or baby corn. The choice of snack was also influenced by the alternatives provided. When the snack was another vegetable, the choice increased intake; however, when the choice was an appealing alternative, such as cookies, there was not an increase in vegetable intake, even with the use of rewards.^73^ Reward types were further investigated by Vandeweghe et al,^74^ who found that a reward increased the consumption of vegetables regardless of reward type (ie, social or token). Scarcity of vegetables was also investigated experimentally by Maimaran and Salant,^75^ who saw significant increases in both carrot and grape consumption when children were told there was a limited supply of these foods.

### Other Intervention Types

Three of the studies included in this review used techniques that did not align with any other studies; therefore, we describe them here.^76–78^ Hughes et al^76^ investigated the impact of a government-led initiative, known as the “School Fruit and Vegetables Scheme,” to provide FV to children in school. By providing children FV in the school setting, the researchers observed that 61.6% of children ate at least 1 fruit or vegetable from the scheme, and mean daily FV consumption increased to 5.4 portions, compared with 4.7 portions prior to the scheme. This finding supports the basic premise that access to FV can increase consumption when these foods are the only available option.

Tani et al^78^ designed a cross-sectional study in which they observed the schools that encouraged children to eat vegetables first at mealtimes saw increased vegetable consumption throughout the meal than the schools that did not implement any vegetable -eating strategies at lunchtime. This is an interesting concept, that encouraging, or “nudging,” the order of which food should be consumed first can positively increase vegetable consumption. This also provides a very easily replicable tool for education settings. However, because only 1 study has investigated this in young children, thus far, more research is necessary to verify this result. Finally, Lumeng et al^77^ used an intervention approach targeted at improving self-regulation in children to improve FV consumption to subsequently prevent obesity. There was no significant effect on food consumption.

## DISCUSSION

Determining ways in which to improve dietary quality and variety of types of foods eaten by children is a continuing challenge for health practitioners. One way to address these problems is to identify evidence-based interventions that promote the consumption of FV by young children.^6–8^ The Evans et al review^17^, published in 2012, and found intake of fruits by older children could be improved, but there was very little improvement in terms of vegetable intake.^17^

Here, we aimed to collate and summarize the existing literature regarding interventions aimed at improving children’s diets within local government-controlled settings for children 3 to 7 years old. A wide array of intervention designs was used, with 6 broad subsets of interventions identified, including educational programs, behavioral modeling, emotional moderation, mindfulness and sensory play, parent-child courses, repeated exposure, and food modification, all aiming to improve consumption of healthy foods and FV. Some articles included designs that incorporated more than 1 methodology, and others included entirely unique designs. Finally, a group of articles included a design that fell into an experimental category.

The intervention designs that appear to achieve the greatest increase in FV consumption were interventions falling under the categories of (1) behavioral modeling and emotional moderation and (2) mindfulness and sensory play. All but 1 intervention included in these 2 categories resulted in increased consumption of FV. Behavioral Modeling is a powerful influence on multiple aspects of the development of children from birth onward, with the first influences being parents and siblings, and then branching out to wider family, peers, and teachers.^79^ The power of modeling has also been widely studied with regard to eating behaviors.^80–82^ It is not surprising, therefore, that interventions implementing this strategy to increase healthy eating behaviors encounter success. Within this group, there is a general use of either peer modeling or the use of a hero character to encourage children to consume more healthy foods, both of which are strategies that could be implemented in group settings with relative ease.

Sensory play and mindfulness approaches were particularly effective: all the articles included in this subset reported success with these methods to increase healthy food consumption. These approaches used all senses to explore food and to become familiar with the types of FVs offered in the interventions. The use of the senses to explore and learn has been associated with positive child development, starting even as early as pregnancy and taste exposure in utero.^83^ Exploration of foods in a novel way, to learn and have fun with them, may increase acceptance through the enjoyment via senses other than taste. Furthermore, a study that used multiple approaches to increase healthy food consumption^63^ combined both behavioral modeling and sensory play. Although this approach was successful, it was not possible to parse out which element of the intervention was having an impact on the increased consumption of FV. Nonetheless, combining these 2 effective approaches within group settings may maximize the likelihood of improving children’s FV consumption.

Of the articles included in this review, educational programs were the majority choice of intervention; however, the relative success of this type of intervention is somewhat lacking. Within the educational programs, only 1 study^31^ noted the impact of the teacher’s perception of the usefulness of the intervention upon its success. The lack of success witnessed in educational interventions could stem from numerous reasons, such as a lack of follow-up in the intervention efforts, particularly in the home setting, or the educational approach that was taken in the interventions and the level of child engagement in the activities.

### Strengths and Limitations

This protocol closely followed the updated JBI methodology for scoping reviews.^19^ With strict appraisal of each screening process, a large number of articles was included for full-text extraction. In-depth exploration of each article led to a large quantity of data available to evaluate all possible methodological relationships. However, the large variety of methodologies used in the studies reviewed posed its own difficulties when synthesizing data and summarizing the results of each intervention type. Methodological variation may affect the comparability of results and the generalizability of findings. Future studies should focus on standardizing intervention methodologies, incorporating long-term follow-up, and exploring the synergistic effects of combining different approaches.

Scoping reviews are designed to answer broader questions and describe the methods that have been used, rather than having the primary goal of determining an intervention’s success; therefore, quality assessments were not performed. Nonetheless, we observed that although most of the studies were of good quality to accurately examine the effects of interventions to increase FV consumption, there were a few that could not determine robust and reliable effects. To help determine the effectiveness of interventions, future research should conduct quality assessments, such as those within a systematic review.

Finally, few of the specific studies included in this review recorded or requested information about the practical application of the interventions, whether the intervention itself not only achieved its goal but also whether it was something that can be easily implemented and continued within the educational settings. This information would add to the understanding of how to ensure effective strategies for improving children’s healthy eating can be practically implemented within group settings for children.

### Conclusion

This scoping review collates and synthesizes current evidence of studies published from 2012 onward that investigated methods to improve children’s diets by introducing new and healthy foods in group-based settings. This scoping review highlights the diverse strategies used to increase FV consumption among young children in group settings. Although many interventions demonstrate potential, their success is often influenced by factors such as implementation fidelity, the specific approach used, and the duration of the intervention. Behavioral modeling, particularly those involving relatable role models and sensory engagement, appears to be the most promising strategy. However, the variability in results underscores the need for further research to refine these interventions and assess their long-term efficacy. The field would benefit from quality assessments conducted through a systematic review as well as standardized methodologies and intervention frameworks.

A tentative pattern emerged, suggesting that behavioral modelling and sensory play resulted in the greatest increase in FV consumption by 3- to 7-year-old children in group settings. However, this result should be viewed with caution because there was no quality assessment of the articles. Vegetables were largely the main targets to improve diet quality. Furthermore, most of the identified studies were carried out with families who had higher-than-average income and education. A proportionate universalism approach needs to be investigated with specific interventions for people of lower socioeconomic background, particularly given the associations with poorer diet quality. By addressing these gaps, researchers and policymakers can develop more robust and scalable interventions to promote healthier eating habits among children from all backgrounds, ultimately supporting their optimal development and well-being.

## Supplementary Material

nuaf092_Supplementary_Data
