# Supplementary material for: Improving Children’s Diets by Introducing Fruits and Vegetables in Group-Based Settings: A Scoping Review
Source: Nutr Rev. 2025 Jul 3;84(5):1039–50. doi: 10.1093/nutrit/nuaf092 (PMC13075485; doi:10.1093/nutrit/nuaf092)
Supplement: nuaf092_Supplementary_Data [file nuaf092_supplementary_data.zip › Supplementary Table I. Search Strategy.docx]

### Supplementary Table I: Search strategy

Searches included the same key terms for each database. Results were limited to only those published in English, from 2012 onwards. An example from Web of Science can be found here: <https://www.webofscience.com/wos/woscc/summary/9fcf2a86-f99c-466b-a19f-cd9486a0ccf7-2ad57581/relevance/1>

| **Topic**  (i.e., title, abstract and keywords) | “child*” OR “student*” OR “pupil*” OR “kid*” OR “schoolchildren” OR “boy*” OR “girl*” |
| --- | --- |
| **AND TOPIC**  (i.e., title, Abstract and Keywords) | “first grade” OR “primary-aged” OR “pre-school” OR “preschool” OR “kindergarten” OR “day care” OR “nurser*” OR “playgroup” OR “playschool” OR “primary school” OR “elementary school” OR “junior school” OR “infant school” OR “extracurricular” OR “classroom” OR “club*” OR “communit*” OR “community-based” OR “key stage 1” OR “3-4” OR “3-5” OR “3-6” OR “3-7” OR “4-5” OR “4-6” OR “4-7” OR “5-6” OR “5-7” OR “6-7” |
| **AND TOPIC**  (i.e., title, Abstract and Keywords) | “intervention*” OR “program*” OR “scheme*” OR “initiative*” OR “project*” OR “campaign*” OR “strateg*” OR “service*” OR “polic*” OR “access*” OR “availab*” OR “provision” OR “provide” OR “suppl*” OR “distribut*” OR “promot*” OR “subsid*” OR “education*” OR “garden*” OR “allotment*” OR “food preparation” OR “prepar* food” OR “cook*” OR “game” OR “experiment*” OR “stud*” |
| **AND TOPIC**  (i.e., title, Abstract and Keywords) | “fruit*” OR “veg*” OR “salad” OR “five a day” OR “five-a-day” OR “5 a day” OR “5-a-day” OR “health* food*” OR “health* eat*” OR “health* snack*” OR “picky eat*” OR “fussy eat*” OR “new food” OR “novel food” OR “unfamiliar food” |
| **AND TOPIC**  (i.e., title, Abstract and Keywords) | “intake” OR “consum*” OR “eat*” OR “diet*” OR “portion*” OR “serving*” OR “choos*” OR “choice*” OR “eat* behavio$r” OR “accept*” |
| **NOT TOPIC**  (i.e., title, Abstract and Keywords) | “secondary school” OR “high school” OR “middle school” OR “prep school” OR “preparatory school” OR “junior high school” OR “intermediate school” OR “college” OR “sixth form” OR “sixth-form” OR “eating disorder” OR “anorexia” OR “ARFID” OR “avoidant/restrictive food intake disorder” OR “avoidant restrictive food intake disorder” OR “bulimia” OR hyperphagia OR "Prader-Willi syndrome" OR "learn* disabilit*" OR "nasogastric" OR “intubation” OR "gastrostomy" OR “g-tube” OR "jejunostomy" OR "feeding tube" OR "tube feeding" OR "autism" OR “neurological disorder” OR “Afghan*” OR “Albania*” OR “Algeria*” OR “Angola*” OR “Antigua and Barbuda” OR “Argentin*” OR “Armenia*” OR “Azerbaijan*” OR “Bangladesh*” OR “Belarus*” OR “Belize*” OR “Benin” OR “Bhutan*” OR “Bolivia*” OR “Bosnia and Herzegovina” OR “Botswana*” OR “Brazil*” OR “Burkina Faso” OR “Burundi*” OR “Cabo Verde” OR “Cambodia*” OR “Cameroon*” OR “Central African Republic*” OR “Chad” OR “China” OR “Colombia*” OR “Comoros” OR “Congo*” OR “Costa Rica*” OR “Côte d'Ivoire” OR “Ivory Coast” OR “Cuba*” OR “Djibouti*” OR “Dominica*” OR “Dominican Republic” OR “Ecuador*” OR “Egypt*” OR “El Salvador” OR “Equatorial Guinea*” OR “Eritrea*” OR “Eswatini*” OR “Ethiopia*” OR “Fiji*” OR “Gabon*” OR “Gambia*” OR “Georgia*” OR “Ghana*” OR “Grenada*” OR “Guatemala*” OR “Guinea*” OR “Guinea-Bissau*” OR “Guyana*” OR “Haiti*” OR “Hondura*” OR “India*” OR “Indonesia*” OR “Iran*” OR “Iraq*” OR “Jamaica*” OR “Jordan” OR “Kazakhstan*” OR “Kenya*” OR “Kiribati” OR “Democratic People's Republic of Korea” OR “North Korea” OR “Kosovo*” OR “Kyrgyzstan*” OR “Lao People's Democratic Republic” OR “Lebanon*” OR “Lesotho” OR “Liberia*” OR “Libya*” OR “Madagasca*” OR “Malawi*” OR “Malaysia” OR “Maldives” OR “Mali” OR “Marshall Islands” OR “Mauritania*” OR “Mauritius” OR “Mexic*” OR “Micronesia*” OR “Moldova*” OR “Mongolia*” OR “Montenegr*” OR “Montserrat” OR “Morocc*” OR “Mozambique” OR “Myanmar” OR “Namibia*” OR “Nauru*” OR “Nepal*” OR “Nicaragua*” OR “Niger*” OR “Nigeria*” OR “Niue*” OR “North Macedonia*” OR “Pakistan*” OR “Palau*” OR “Panama*” OR “Papua New Guinea*” OR “Paraguay*” OR “Peru*” OR “Philippines” OR “Filipinos” OR “Rwand*” OR “Saint Helena” OR “Saint Lucia*” OR “Saint Vincent and the Grenadines” OR “Samoa*” OR “São Tomé and Príncipe” OR “Senegal*” OR “Serbia*” OR “Sierra Leone*” OR “Solomon Islands” OR “Somalia*” OR “South Africa*” OR “South Sudan*” OR “Sri Lanka*” OR “Sudan*” OR “Suriname*” OR “Syrian Arab Republic” OR “Tajikistan” OR “Tanzania*” OR “Thailand*” OR “Timor-Leste” OR “Togo” OR “Tokelau*” OR “Tonga*” OR “Tunisia*” OR “Turkey” OR “Turkmenistan” OR “Tuvalu*” OR “Uganda*” OR “Ukrain*” OR “Uzbekistan” OR “Vanuatu” OR “Venezuela*” OR “Vietnam*” OR “Wallis and Futuna” OR “West Bank and Gaza Strip” OR “Yemen*” OR “Zambia*” OR “Zimbabwe*” |
| **NOT TITLE** | “Disease” |
