# Supplementary material for: Improving Children’s Diets by Introducing Fruits and Vegetables in Group-Based Settings: A Scoping Review
Source: Nutr Rev. 2025 Jul 3;84(5):1039–50. doi: 10.1093/nutrit/nuaf092 (PMC13075485; doi:10.1093/nutrit/nuaf092)
Supplement: nuaf092_Supplementary_Data [file nuaf092_supplementary_data.zip › Supplementary Table II. Data Extraction Instrument.docx]

### Mbi Supplementary Table II: Data extraction instrument

| Author(s) |  |
| --- | --- |
| Year of publication |  |
| Country of origin (i.e., where the study was published) |  |
| Local setting (if applicable) |  |
| Aims |  |
| Study/intervention duration |  |
| Study population (including age, % male, other special characteristics if applicable e.g., low income, ethnicity etc.) |  |
| Number of participants (sample size) |  |
| Methodology |  |
| Outcomes (i.e., types of food, how consumption measured) |  |
| Intervention type and duration, comparison, outcome measures (if applicable) |  |
| Details of statistical analyses (if applicable) |  |
| Key findings that relate to scoping review question(s) |  |
| Comments |  |
