# Supplementary material for: Improving Children’s Diets by Introducing Fruits and Vegetables in Group-Based Settings: A Scoping Review
Source: Nutr Rev. 2025 Jul 3;84(5):1039–50. doi: 10.1093/nutrit/nuaf092 (PMC13075485; doi:10.1093/nutrit/nuaf092)
Supplement: nuaf092_Supplementary_Data [file nuaf092_supplementary_data.zip › Supplementary Table III. Summary of Studies.docx]

| **Table III.** **Summary of Methodology, Outcomes and Key findings of study included across all included articles (N=114)** | | | | |
| --- | --- | --- | --- | --- |
| Author | Intervention Type | Number of participants | Outcomes | Key findings that relate to scoping review |
| (Adab et al., 2018) | Multiple methods | 1467 participants were recruited, 1249 pupils continued to first follow up and 1145 at the second follow up. | The protocol defined primary outcomes, assessed blind to allocation, were between arm difference in body mass index (BMI) z score, at 15 and 30 months. Secondary outcomes were further anthropometric, dietary physical activity, and psychological measurements and difference in BMI z score at 39 months in a subset. The difference between BMI z scores at 15 and 30 months. Dietary intake measured using the Child and Diet Evaluation Tool (CADET). Body fat % Waist Circumference, Skinfold Thickness, Physical Activity, Blood Pressure, Quality of Life, Social Acceptance, Body Image Dissatisfaction. | Diet was statistically non-significant between groups at both follow ups. |
| (Ahern et al., 2019) | Repeat Exposure | 95 children. | Anthropometrics.  Parental Food Frequency Questionnaire.  Parental measure of Food Neophobia.  A child FFQ.  Child Food Neophobia Scale,  And the Child Eating Behaviour Questionnaire. | Pre-intervention consumption did not vary as a result of snack type. Children in the repeat exposure variety group consumed similar amounts of the variety and single snack. Those in the repeat exposure single snack type at significantly more of the mixed vegetable snack.  Children consumed more red pepper than the other two single vegetable snacks.  Post intervention intake showed that the single vegetable snack was significantly higher than the mixed vegetables. This was significant for the RE group but not the V group.  There were no post intervention differences in the number of vegetables consumed for the single snacks.  ANCOVA analysis revealed that there was a significant effect of time pre to post-test consumption of the vegetables. Children in the V group showed a significant increase in mixed vegetable consumption post-test, showing a significant interaction effect. |
| (Anzman-Fransca et al., 2012) | Repeat Exposure | Experiment 1: 41 Children Experiment 2: 43 Children | Food Liking measured as “Yummy”, “Yucky”, or “just OK” Food intake measured as weighed amount in grams from before and after intake session.  Anthropometric Measures of height and weight were also used to calculate BMI. | Experiment 1.  There was also significant increase in vegetable intake from pre-to post test. This was an average increase of 16.8 grams. Of vegetable.  There was a positive linear relationship between liking of vegetables and vegetable intake. This was also found for experiment 2. |
| (Bai et al., 2018) | Education Programme | 71 children participated. | Vegetable consumption behaviour  Vegetable intention to eat. | There was no significant difference in the baseline measures for consumption behaviour, intention and personal factors. Following the intervention there was a significant improvement in the vegetable consumption behaviour of those in the intervention group. The control group children showed no change for any of the measured variables. |
| (Bell et al., 2023) | Multiple methods | 1039 total children took part. 129 in control, 105 in curriculum only condition, 143 in curriculum and mealtime environment condition, 162 in curriculum and food provision condition, 144 in food provision only condition, 122 in food provision and mealtime environment condition, 121 in mealtime environment only condition, and 113 in curriculum, food provision, and mealtime environment condition. | Data collected at baseline and follow up 12 weeks later. Outcomes include children's vegetable intake (gram/day) and vegetable provision and waste and initiative fidelity and acceptability. | No statistically significant two-way or three-way interactions between initiatives suggest the effect of one did not depend on the other. Main effects of initiatives found no statistically significant differences in vegetable intake when compared to control for children receiving the mealtime environment, curriculum, and the food provision initiatives. No statistically significant differences in any of 7 intervention groups in vegetable intake. Vegetable intake was 3.29x higher in curriculum with mealtime environment initiative than control (26.69g per day). children receiving all three initiatives consumed 1.34x more vegetables than control (equivalent to 4g). |
| (Belot et al., 2016) | Emotional moderation/Self-regulation/mindfulness | 638 Children | Background and Anthropometric data.  School meal observation, including consumption of vegetables recorded as none, some, more than half of that fruit or vegetable.  Food knowledge test identifying if a food was healthy or not.  Choice of fruit and vegetable after incentives were removed. | The competitive incentive scheme was found to increase the amount of fruit or vegetable tried (consumed) by 11.2 percentage points. There were no positive effects for the individual incentive scheme. The effects of consumption were found in both age groups and therefore are relevant to the 6-7 year olds. Although there is little evidence for persistence in the competitive group except for the girls in year 2. |
| (Berlic et al., 2023) | Food modification | 57 children from six kindergartens 36 boys, 21 girls | Dietary intake measured by weight during kindergarten time and at home by parents. | For the prototype group, proportion of vegetables consumed ranged from 7-49%, followed by vegetable soup (13-45%). The control group proportion of vegetables was 12-70%. The meals in the prototype group align more closely with the daily recommendations than the control group. Significantly more vegetables, whole grains, and nuts, were offered in the prototype group than control. More refined foods and potatoes, and fruits were offered in control group. |
| (Boyer et al., 2012) | Food modification | 28 children recruited 21 completed. | Weighted of snack consumed was measured for each snack in each condition, either normal or shaped.  Energy intake was calculated from weighted consumption of snack. | There was no significant difference in average snack consumption between the shaped and normal form snacks. |
| (Braga-Pontes, Simões-Dias et al. 2022) | Education Programme | 180 recruited, 162 completed | Consumption of study vegetables, tomato, purple cabbage, cucumber, carrot and lettuce, at Baseline, Post-test and Follow-up. | Children allocated to the control group at more portions of carrots and lettuce at baseline compared to other groups. Children in the control group also had higher consumption of the purple cabbage and cucumber than children in the DG and SB groups at baseline. And also, higher consumption of tomatoes compared to the SB groups.   At post-test children who were in the control group ate significantly more portions of carrot than children in the other groups, and there were no differences for other vegetables.  At the follow-up children in the SB group at significantly more portions of carrot than children in the SBS group. Children in the control group also had significantly higher consumption of purple cabbage than children in the SBS and SB groups.   A significant time x group interaction was found for carrot consumption only. For all vegetable there was a significant increase in consumption from Baseline to Post-intervention and follow-up.   In the SBS group there was a significant increase in the consumption at post-test and follow up of Carrot, cucumber, and tomato. |
| (Brennan et al., 2021) | Education Programme | 1100 6–7-year-old students and 1018 10-11 year old students were recruited | KIDSCREEN-10 questionnaire at baseline and endpoint.  Strengths and Difficulties Questionnaire (SDQ) – Teacher completed at baseline and endpoint.  Food Frequency Questionnaire (FFQ) completed by the 10-11 year old at home. 6-7 year olds completed a condensed version administered at baseline and endpoint.  Any food knowledge and Food identification and food Neophilia completed at Baseline and endpoint for all age groups,   Perceived cooking competence, this was not completed for the younger group. | For the nourish intervention, 86% of children responded with ever when answering about vegetable consumption, compared to only 74% of those who had not received the intervention. Those who completed the nourish were less likely to ever consume beef. |
| (Bucher Della Torre et al., 2015) | Parent-Child Course | 21 children from 19 families registered. The final sample included 18 children from 15 families. | Changes in child knowledge about fruit and vegetables.  Changes in child interest in fruit and vegetables.  Willingness to taste. | Average fruit and vegetables known increased from 10 an median of 10 (IR = 9.5), to 11 (IR= 9.0) after the programme. It was not statistically significant.  The median number of fruits and vegetables tasted did not change before and after the programme. |
| (Bucher Della Torre et al., 2023) | Repeat Exposure | 45 children aged 5-6 years. 19 boys and 26 girls | Preferences of 10 food items (8 of which were fruit and vegetables). Each child asked to indicate preference for presented food item on 5-point likert scale. Willingness to taste measured by children invited to taste 5 vegetables from game (carrot, tomato, lettuce, cucumber, and red cabbage). Number of different vegetables tasted were counted. | 15% more children were willing to try red cabbage after intervention than before, 7% for lettuce and carrots, 2% for cucumber and 1% for tomatoes. But none of these were significant. |
| (Capaldi-Phillips & Wadhera, 2014) | Repeat Exposure | 29 children. | Vegetable consumption – weighted.  Cream Cheese consumption – weighted.  Liking for Brussels Sprouts.  Liking for Cauliflower.  Brussels Sprouts Consumption – Weighted.  Cauliflower Consumption. | There were no significant differences in the consumption of each vegetable either brussels sprouts or cauliflower and no interaction by group.   There was no significant interaction between vegetable and cream cheese flavour on the amount of cream cheese consumed.   The consumption of brussels sprouts did not vary as a function of training but did vary significantly as a function of liking. Children who liked brussels sprouts more after training ate more of them compared to children who reported disliking them. A similar pattern was found with Cauliflower. |
| (Carney et al., 2018) | Food modification | 44 parent child dyads completed both test meal visits. | Liking and preference were measured for the vegetables consumed.  PROP taste tests categorised as taster vs non taster.  Food neophobia, temperament, clinical nutritional risk, typical eating behaviours, infant feeding practices, and caregiver feeding style was measured as well as Anthropometrics.  Carrot consumption was calculated as the difference in grams between pre-weight of food and post weight. This was then converted to the proportion of children’s total carrot intake. | There were no significant differences in the meal items consumed between the variety and no variety conditions. During the variety meal children’s average intake of the three types of seasoned carrots did not significantly differ between seasoning type. In the no variety condition the intake of cinnamon carrots did not differ between the three containers.  Children consumed an average of 36.54 g of carrot in the variety condition and 35.85 in the no variety condition which was not significantly different. |
| (Carstairs et al., 2018) | Food modification | 43 children (50 recruited, 7 excluded) 23 girls and 20 boys. | Parental feeding practices.  Intake of High energy Density (HED) sandwich (g) Intake of Low energy Density (LED) sandwich (g) Intake of dessert (g) Total energy intake (kcal) Food consumed measured as the difference between pre and post meal weights | Significant effect of portion size upon HED sandwich consumption. 40% downsizing led to 21% lower average intake There was no effect of vegetable condition upon HED sandwich consumption There was no interaction between portion size and vegetable condition.  There was a significant effect of portion size on total energy intake.  9-12% reduction in the 60% portion size compared to the 100%.  No significant effect of vegetable condition.  HED portion size had no significant effect of intake on vegetable Significant effect of vegetable condition – the mixed vegetable variety group consumed on average 77g more.  There was equal intake of all vegetables (carrot cucumber and cherry tomatoes) in the mixed group. |
| (Chen et al., 2014) | Multiple methods | Intervention 604, control 600 students. | Student familiarity with the featured vegetable was measured. Students preference for the featured vegetable.  Parent outcome variables before and after the intervention were included. | Based on parent surveys, Students showed significant increase in consumption of all seven vegetables pre- to post intervention. When compared to control groups this liking was significantly more for Jicama, bell pepper and asparagus, after adjusting for ethnicity and grade. |
| (Choi et al., 2018) | Education Programme | 35 participants | Intakes of fruits and vegetable in subjects.  The child behaviour checklist scores, DSM-5  Nutrition education. | The intake of vegetables increased significantly from 36.15gs pre intervention to 48.01 g post intervention. The number of picky eaters decreased from 5 to 4, although this was not statistically different. |
| (Correia et al., 2014) | Food modification | 57 children participated. | Willingness to taste described as consumption of 3g or more of a given food item.  Amount of food consumed as pre-and post-weight in grams was recorded to calculate amount consumed of a given meal. | In the pairing intervention, the consumption of the target vegetable, Broccoli did not differ significantly between the experimental condition and the control condition. Consumption of other meal components also did not change as a result of the intervention, except for total pizza intake.   Children ate 14.3g less pizza when it was topped with broccoli compared with when it was plated on the side.   More children tried broccoli when it was served on top of pizza compared to when it was served on its own, 95.3% and 79.1% respectively.   In the visual appeal intervention, there was a non-significant increase in the amount of cucumber consumed in the visual appeal group 34.3g compared to the control group 29.0g. There was also a non-significant difference in the willingness to try from 78.6% in the control condition to 83.3% in the intervention group. |
| (Cosco et al., 2022) | Education Programme | 285 children aged 3 - 5 years. 61 in intervention group, 119 in waitlist and 70 in control. | Fruit and vegetable identification measured by whether the child reported to know the fruit / vegetable. Fruit and vegetable liking measured by on likert scale between 1 - 5 (1 = super yucky, 5 = super yummy). Fruit and vegetable consumption measured by weight in grams from 6 pre-portioned 50g servings of different fruit and vegetables. | FV consumption increased in the intervention group, but not in the non-intervention groups. Children in intervention group consumed 25g more fruit and 14 more vegetables than control groups. |
| (Coulthard & Ahmed, 2017) | Education Programme | In total there were 102 children that took part, 30 in the real FV game, 24 in the picture FV game, 24 in the real FV sorting game and 24 in the picture FV sorting game. | Consumption of Novel Fruit (Pomegranate),  Consumption of Novel Vegetable (Soya bean) Changes in picture preference ratings of fruits and vegetables. | In the tasting task the mean consumption of pomegranates was 9.00gs and the mean consumption of soya bean was 4.91g. Children ate significantly more of the fruit compared to the vegetable in the tasting task.   Consumption of pomegranate varied as an effect of the stimuli used whether it was real fruit and vegetables or photographic fruit and vegetable. With significantly higher consumption 10.9g in the real fruit condition compared to the photo stimuli 6.89g. There was no significant change in consumption based on task.   Soya bean condition also varied as a result of the stimuli used with significantly more consumed using real fruits and vegetables 6.52gcompared to photographs 3.10g. There was also a significant difference in the amount consumed as an effect of task type with more being consumed in the Game task 6.07g, compared the categorisation task 3.60g. |
| (Coulthard & Sealy, 2017) | Emotional moderation/Self-regulation/mindfulness | 62 children in total, 21 in the fruit and vegetable sensory play, 21 in the non-fruit and vegetable play, 21 in the visual exposure. | Child Neophobia food scale Tactile sensitivity,  Fruit and vegetable portions consumed for parents and children.  Baseline liking for each of the experimental foods.,  Experimental foods tasted. The food was given a score of 1 if the child placed it in their mouths, whether they swallowed or not. The number of foods tasted ranged from 0-8. A score for foods featured in the conditions range 0-5, and foods not featured in any condition 0-3. | There were statistically significant negative associations between fruit and vegetable consumption and child neophobia.  After controlling for covariates there was as significant difference between the groups for the number of food s tried. Children in the FV sensory play group tasted more foods than either the non-food sensory play group or the visual FV exposure group. There was no difference between the sensory play non-food group and the visual FV exposure group.   For foods that were featured in the experimental task there was significantly more tasted in the sensory play FV group compared to the sensory play non-food group, and the visual FV group. Again, for the foods that were no featured there was a similar pattern with the sensory FV group tasting more than the other groups. |
| (Crespo et al., 2012) | Parent-Child Course | 808 children | Parent and child BMI Children’s Physical Activity Levels Children’s sport Participation Active Transportation to and from School Availability and use of Active Toys Parental Support for Child Physical Activity.  TV Viewing Children’s Dietary intake measured using a 49 item food frequency questionnaire (FFQ).  Parenting Style for Diet and activity Behavioural Strategies for Fat and Fibre | The family only intervention significantly increased the daily consumption of fruits and vegetables from Month 1 to month 4. This is from 1.89 fruits and vegetables per day to 2.31 in month 4. For the community only study there were no significant changes. |
| (De Bock et al., 2012) | Modelling TV Books Toys Characters | 377 children | Anthropometric and body composition measurements were taken.  Children’s eating behaviour and physical activity was completed by the child’s parents.  Parental reports of specific foods and beverages, including fruits and vegetables.  Consumption of Water using an FFQ item. | At baseline 60.0% of children achieved the recommended daily intake of fruit (200g), and 34.6% for vegetables. From pre- to post- intervention there was a significant change from baseline in fruit and vegetable intake, with a mean intake of 0.17 points for fruit and 0.22 points for vegetable consumption, when adjusting for the other characteristics. A change of 1 point is approximately 1 portion.  Participating in the intervention was associated with increased vegetable consumption by 0.15 points. |
| (De Coen et al., 2012) | Education Programme | At the beginning 1280 parents completed the questionnaire, at the 2 year follow up only 694 questionnaires were received covering 1102 children, this meant a drop out of 586 children. Children with a low ses were much more likely to drop out. | BMI and parental questionnaire  Demographics,  24 item food frequency questionnaire were collected. Diet records of 3 days were taken.  Physical activity was assessed through parental questionnaire asking about sports club membership of their child. | There were no significant intervention effects for consumption of fruits, vegetables, water, milk, soft drinks and savoury snacks. |
| (de Droog et al., 2014) | Modelling TV Books Toys Characters | 160 recruited in total 104 children in the experimental group and 56 children for the baseline group. | Cognitive response to carrots.  Automatic affective response to carrots.  Elaborate affective response to carrots.  Product consumption. | Cognitive response positively influenced children’s carrot consumption through elaborate effective response. The Structural equation model accounted for 31% of the variance in carrot consumption. Elaborate effective response positively influenced carrot consumption.  Interactive shared reading positively influenced consumption via the automatic and elaborate affective responses mediating pathway.   Final point is that the impact of interactive shared reading upon carrot consumption is fully mediated by automatic and elaborate affective responses toward carrots. |
| (DeJesus & Venkatesh, 2020) | Education Programme | 71 children. | Food intake was measured as post-test food weight subtracted from the pre-test food weight in grams.  BMI,  Two subscales of the Children’s Eating Behaviour Questionnaire, Food fussiness and Food Responsiveness. | There was no significant effect of task order, modality of modelling, or interaction upon the amount of food consumed.  Only the CEBQ Food Fussiness subscale was associated with Children’s food intake, the pickier the child the less the children ate in this study. |
| (DeJesus et al., 2019) | Experimental | In total there were 7 studies, each study recruited 32 children (bar study 6 that recruited 33) – therefore a total of 22 4 children were recruited. | Food consumption was the primary measure of interest for these studies and was measured as the number of bites of food children took.  Food was also weighed before and after consumption and recorded in grams.  Food liking was recorded, this ranged from “not yummy at all” to “really, really, yummy”. | Study 1, Children ate more food described as healthy with the number of bites showing a mean average 6.28 compared to a mean of 2.03 for unhealthy. There was no effect of gender observed.  Study 2. Children ate significantly more of the food described as healthy but not popular compared to the food described as popular but not healthy. Again, no significant effect of gender.  Study 3. Children ate more of the neutral food than the unhealthy food (M = 7.62 bites compared to 3.72). No significant effects of gender.  Study 4. No significant effects of food type, the children at similar amounts of the neutral and healthy types of food.  Study 5. Children ate significantly more food described as unpopular compared to the food described as unhealthy (mean 5.25 bites vs 2.75). No effect of gender. |
| (Dial et al., 2020) | Emotional moderation/Self-regulation/mindfulness | 52 pre-schoolers, 30 parents, 27 children in intervention group and 25 in control. | Dimensions measured Number of senses used to explore foods Number of senses used to explore toys Number of descriptive words used for food Number of descriptive words used for toys Number of foods tasted during exploration Number of foods tasted during neophobia phase The average rating of foods that were tasted. A facial scale was used to rate the food tastes from very sad to very happy. | Pre test- Post test (Intervention Children: IC). Intervention children not significant for number of foods tasted during exploration. Intervention children not significant for number of foods tasted neophobia. Intervention children significant for rating of foods tasted more favourably pre-test. Pre test- Post test (Control Group: CG). Not significant for number of foods tasted during explore Not significant for number of foods neophobia or for rating. Group comparison Intervention children not more likely to try foods in either explore or neophobia. Intervention children also not more likely to try food Post-Test. During intervention the majority of children tried foods novel and familiar in the intervention group. Exposure only children most declined radishes. |
| (Diktas et al., 2021) | Food modification | 67 children. | Weighted meal intake, for both food and beverages in grams.  Energy intake was calculated from weight of the meal in kcal.  Energy density was also calculated in kcal/g Food liking and preferences.  Body weight and height. | Intake of vegetables at the meal was significantly affected by serving a larger portion, but not by enhancing the flavour. Vegetable intake increased by 21g (SD= 3g), when the portion of vegetables was doubled.   Children consumed similar amounts of plain and enhanced vegetables. Flavour enhancement did not modify the effect of larger portions.   When removing the 9 children who ate all the vegetables served at three or more meals the effects remained significant.   Doubling portion sizes of both broccoli and corn increased consumption significantly. Children ate similar amounts of plain and enhanced broccoli however there was a significant decrease in the amount of corn consumed when it was enhanced. |
| (DiSantis et al., 2013) | Food modification | 41 children participated. | Self-serve portion sizes were measured pre and post consumption, which also measured energy intake. Children’s body weight and height was calculated. A Food-liking assessment was also carried out. | There was a main effect of Dishware size upon the total self-served energy. On average children served 90.1 kcal more when they were given an adult sized dish, compared to a child-size dish.  This effect was seen in about 80% of children. The type of entrée offered also influenced the amount that was served, with self-servings being higher in energy with unit entrée compared to amorphous entrees.  When liking was higher the serving sizes were also significantly higher by an average of 104.2 kcal. Children also served themselves 15.7kcal more of fruit when they had an adult sized dish. They served themselves 62.9 kcal more fruit when they liked the fruit. For the vegetable side, only the liking influenced the portion size with a 42.3kcal increase when the children liked the size. |
| (Domínguez et al., 2013) | Experimental | 150 children | Child’s preference for vegetables was assessed for 6 different vegetables including, chard, spinach, zucchini, green beans, cauliflower, and peas.  Vegetable consumption, of either zucchini and/or green beans was recorded depending on which experimental condition the children were in. ~Vegetables were boiled and dressed with salt and olive oil and presented with the vegetable only as a novel methd as they are used to seeing these its meat and potatoes. The pre-and post-consumption weight was recorded in grams. | A significant effect of condition x children’s total vegetable intake, without any additional interaction effects (condition x school x age x gender) was found. Specifically, vegetable intake differed significantly between the NCC and the DCC as well as between the NCC and the CDCP. Statistical confirmation of these results was obtained from the one-factor ANOVA and post hoc analyses. There were no differences between the DCC and the CDCP groups. |
| (Edwards et al., 2022) | Modelling TV Books Toys Characters | 117 parents and children participated. The final sample included 111 participants. | Childrens Eating Behaviour Questionnaire (CEBQ)  Child Food Neophobia Scale Randomisation checks.  Child willingness to consume broccoli measured as frequency of tastes during a video observation. | Sixty-seven % of children swallowed at least one bite of raw broccoli, in the subset where 5 participants were excluded due to poor recordings. There was no significant main effect of condition on the willingness to try broccoli.  In the subset whereby 13 participants were excluded from the video analysis due to inadequate recording (could not determine the number of oral exposures), the frequency of tastes revealed a significant effect of condition, whereby number of tastes were significantly higher in the positive facial expressions group compared to the no food condition, but not the neutral condition. Neutral and No food also did not differ significantly.  ANCOVA results controlling for food fussiness, showed that there was a significant main effect of condition, that positive showed higher intake compared to the no food condition, but not the neutral condition and that neutral and no food conditions did not differ significantly |
| (Elrakaiby et al., 2022) | Modelling TV Books Toys Characters | 69 children with 16 teachers. | Pre and post programme differences in: Proportional consumption, and total consumption of broccoli.  Liking of Broccoli. | The average total consumption of broccoli increased significantly by 35% or 0.14 ounces after the programme.  The average proportional consumption of broccoli increased by 18.8%, however this was not significant for all children. The mean proportional consumption of broccoli increased by 28% for those children who has received 5 or more readings of the book.  The number of readings of the book was a significant predictor of post-intervention proportional consumption of broccoli. |
| (Farrow et al., 2019) | Multiple methods | 74 children | Change in liking ratings of food Change in vegetable consumption Child hunger was measured using the Teddy Bear Hunger rating Scale.  Parental measures of the children’s food fussiness using the 6 item scale from Children’s Eating Behaviour Questionnaire (CEBQ).  Previous exposure to the 4 experimental vegetables was measured prior to the intervention and recorded on a 5 point Likert ranging scale from 1(never offered) to 5 (offered more than 10 times). | There was a significant main effect of which app group children were assigned to upon the amount of vegetables consumed. Children in the Vegetable maths masters consumed significantly more, 4.9gs, compared to the control app 3.8g. There was no main effect of exposure, whether vegetables were exposed in the app or not, upon consumption. |
| (Fisher et al., 2012) | Food modification | 152 | Dressing and Vegetable Familiarity and liking pre-and post-exposure.  Broccoli Intake during the repeated exposure.  Post-exposure vegetable medley intake assessment.  Bitter Taste Sensitivity.  Body Mass Index Child Neophobia.  Parent Report of Child Familiarity with Broccoli and Dip. | During the exposure 75% of children reported having eaten Broccoli previously. Half rated Broccoli as being ‘Yummy” 17% said it was “just okay” and 31% said it was “yucky”. The dressing was liked by most children with 66% saying that they had tried either the full fat or reduced energy version before.  A main effect of week on Broccoli was found, where children’s mean intake of Broccoli changed from the first week 13.4g to the last exposure 9.3g, the highest intake occurred at week 2, exposure 3 and 4. Of those who ate nothing during a given week 67% - 78% were bitter sensitive.  Children with higher neophobia were associated with the lowest broccoli intake across the 7 weeks.  The main effect of dip condition on broccoli consumption was not significant. However, the effects of condition interacted with genetic sensitivity to bitterness, those who were bitter sensitive ate about 50% less broccoli in the plain group compared to other conditions with dip.  Broccoli liking increased post-exposure, showing 18% increase in those finding it ‘yummy’. |
| (Garcia, Brown et al. 2020) | Parent-Child Course | 142 parent child dyads – 121 after attrition, 64 intervention group: 57 control group. | Food fussiness score measured with a questionnaire. Willingness to try vegetables at tasting party. Food fussiness was measured using 6 questions from the CEBQ – modified. Measured before week 1 and after week 4 of the intervention. Tasking Party scores were calculated for each vegetable both raw and code – Scored 1-3. 1 = did not try, 2= tried it/ate some, 3 = at all. Wide variety of vegetables used. | Intervention group showed significant increase in willingness to try at tasting party for both raw and cooked foods. Control group showed significantly lower willingness to try cooked foods compared to raw. The intervention group showed significant increases in willingness to try for all vegetables. For both groups children were significantly less willing to try cooked spinach compared to raw spinach. There was no significant difference in willingness to try between raw and cooked food for any other vegetables. |
| (Gomes, Barros et al. 2018) | Parent-Child Course | 743 invited. 353 returned the evaluation protocol. 138 assigned to the complete intervention group and 120 assigned to the minimal intervention group. 91 assigned to the control group. | Parents perception about the child’s current weight.  Parent’s four item question was developed to evaluate parent’s perception of their efficacy in promoting their children’s healthy food intake.  Parent’s nutritional knowledge questionnaire.  Children’s eating habits questionnaire CEHQ  Caregivers feeding styles questionnaire. | For the Complete intervention group there were significant differences between baseline and post intervention healthy food consumption, with a significant increase in frequency of healthy food intake.  For the minimal intervention group there was a significant decrease in frequency of healthy food at 6 months compared to the post intervention.  The complete intervention group differed significantly in healthy food intake with significantly higher food intakes post intervention, 6-month assessment and at the 1 year assessment. |
| (Gripshover & Markman, 2013) | Education Programme | 59 children | The number of pieces of each food that the children ate was recorded by an observer, who was blind to the condition that the child was assigned to.  Three indices of child food acceptance were also recorded,  1. Total Unique foods- the number of combined typical and new foods (0-8) of which children ate at least one piece.  2. Unique new foods – new foods (0-4) of which children at one piece.  3. Total vegetables – Total pieces of vegetables (0+) that children at during snack time. | There were no differences found between conditions for the total unique foods eaten or the unique new food that children selected at snack time. There was an increase in the intake of vegetables pre to post intervention for the intervention group only. This increased from a mean average of 3.8 pieces to 9.07 pieces. The control group did not show an increase, although they ate more at pre-test, 6.9 pieces, and maintained the same consumption rate of 6.8 post-test. |
| (Halbeisen & Walther, 2021) | Repeat Exposure | 40 in Experiment 1, and 42 in Experiment 2. | Experiment 1- Food choice was measured by how many heart shaped pieces of foam a food received. Additionally which foods were chosen in a grocery scenario.  Experiment 2 – Food consumption was measured for a conditioned vegetable – e.g. carrot. | In line with the study hypotheses, children awarded more hearts to the unconditioned liked stimuli rather than the neutral stimuli. When investigating the effect of different conditioned stimuli, it was found that this was non-significant for both unconditioned liked and unconditioned neutral, therefore the liking did not differ between different conditioned fruits or vegetables.  In the grocery task the conditioned stimuli fruits and vegetables were significantly more likely to be chosen when paired with unconditioned liked (75% of the time).  In experiment 2 again more hearts were awarded to the happy stick figure stimuli pairings, compared to the neutral.  During the consumption task, it was found that children consumed significantly more carrots whilst listening to the audio play when Carrots were paired with the happy stick figures compared to the neutral stick figures. |
| (Harms et al., 2023) | Multiple methods | 169 completed at least one dietary assessment so were included in analysis. 41 completed full intervention, 47 partially completed intervention, and 81 were in control group. | Dietary intake assessment - 24 hour dietary recall method (parents via telephone). Covariates included gender, age, country of birth, educational level, employment status, and weight and height. | Sample size too small to identify statistically significant differences within groups over time. Full intervention showed highest average intake of fruit at timepoint 1 and 2. Partial intervention and control groups consumed near recommended daily average of 150 grams. Within group effect was small. Both intervention groups showed large increases in vegetable intake at timepoint 1. Within group effect sizes again small. Full intervention saw steady increases in fruit consumption, partial intervention saw decrease at timepoint 1, but return to baseline at timepoint 2. For vegetables, increasing proportions seen for both intervention groups at timepoint 1, but decreased by follow up. 22.6% of partial intervention group managed to meet fruit recommendations, but 46.2% of full intervention group met recommendations by follow up. This was 48.4% for partial intervention and 38.5% for full intervention for vegetable consumption recommendations at follow up. |
| (Harnack et al., 2012) | Experimental | 53 children | Fruit and vegetable intake in serving sizes  Energy intake in kcal. | Significant differences were found in food and nutrient intake when comparing the fruit and vegetable first and control conditions. The fruit and vegetable first condition showed an average of 0.4 servings per meal compared to 0.32 servings.  During the provider portioned and control conditions there were a number of differences in food and nutrient intake. There were significantly higher intakes of grains, meat and milk in the provider portioned group compared to the traditional family style.  Fruits and vegetable consumption was significantly lower in the provider portioned group.  Energy intake was significantly higher in the provider portioned meal, 284 vs 223 kcals. |
| (Hoppu et al., 2015) | Emotional moderation/Self-regulation/mindfulness | A total of 68 children participated, 44 intervention and 24 control. | Willingness to eat. This was recorded at baseline and after the intervention for each vegetable or berry sample, and noted down as, not tasted, tasted a bit, ate half, or at the whole sample.  A baseline parental questionnaire was completed to record demographic information such as year of birth, education, employment situation, and smoking status.  The food neophobia scale (Finnish version) was completes,  Parents were also asked to evaluate how much their children liked the vegetables and berries used in the experiment. | Looking at total samples tried, there was a significant difference between baseline and post intervention for the intervention group.  There was no significant change within the control group.  Specifically, in the intervention group, the number of samples completely eaten post intervention significantly increased.  Of the vegetables and fruits, the samples that increased the most were carrots, swede, and bilberries in the intervention group. There was also a significant difference in the median intake of carrots, cabbage, swede, rucola, bilberries and lingonberries.  In the control group, only the willingness to eat the romaine lettuce significantly increased between baseline and the post 5-week measure. |
| (Hughes et al., 2012) | Other | A total of 3296 children were recruited. This was decreased to 2306, when all outliers and geocoded data was taken into consideration. | Frequency of fruit and vegetable consumption was measured and related to deprivation and geographic location | 61.6% of the children ate at least one fruit or vegetable from the SFVS, 59.2% of the children ate fruit and vegetables 5 or more times per day.  Mean daily Fruit and vegetable consumption was 5.4.  When SFVS fruit and vegetables were excluded 48.8% of the children at 5 or more per day and the average consumed was 4.7. |
| (Johnson et al., 2019) | Repeat Exposure | 250 families, retention of 70% | Researcher observed assessment of willingness to try new foods and consumption of new foods at 4 time points, baseline, post intervention, Year 1 (Y1) and Year 2 (Y2) follow ups. | Consumption of target food. Significant differences between groups in Jicima consumption pattern. Intervention group ate significantly more Jicima post intervention. The control group at significantly more at the Year 2 follow up. There was no significant change in intake between groups at Year 2 post intervention. No difference in amount consumed between groups for edamame. |
| (Jones et al., 2015) | Modelling TV Books Toys Characters | Total number is 122, 62 in the intervention group and 60 in the control. | Service Characteristics.  Healthy eating and physical activity policy and practice implementation Child dietary intake – Amount served less the amount remaining +- amount wasted.  Child Physical activity Acceptability of the intervention . | There were no significant differences between groups at follow up in the mean number of serves consumed by children for each food group. |
| (Joseph et al., 2015) | Education Programme | A total of 49 children were recruited. | Children’s ability to identify certain snack foods.  Children’s knowledge and preference for snack was assessed using the Preschool Snack Selection tool (PSS).  Children were weight and measured using digital scale. BMI was calculated to establish BMI z scores.  Snack choice and the amount consumed was recorded by one of two observers. The snack options were either healthy or unhealthy | There was no significant improvement in snack choices, between the grapes or cookies.  Children who were younger were more likely to choose the healthier of the snack choices after the intervention. |
| (Karagiannaki et al., 2021) | Repeat Exposure | 159 children. | Comprehensive Feeding Practices Questionnaire.  The Children’s Eating Behaviour Questionnaire.  Food Neophobia 6 item Questionnaire.  Familiarity and liking of stimuli. | The control group showed an initial intake of 13 g at baseline rising to 35g at post-test, increasing again to 54g at 3- months follow-up and then 85g at 6-months follow-up. All significant increases.  For the exposure group the twice weekly showed a pattern of increase from baseline to post intervention from 30g to 61g, a slight decrease at 3-months, 54g, and a peak at 6-months of 110g. Baseline-to follow-ups are all significant.  The once weekly group showed a pattern of 9g baseline intake which is the lowest baseline, rising to 64g at post-intervention, dropping to 40g at 3-months and rising at 82g 6-months after the intervention. Overall change was found to be significant.  The once every other week group showed a large increase from baseline to post intervention 15g to 75g, rising to 82g at 3-months and then 90g at 6-months, all significantly higher than baseline. |
| (Karagiannaki, Ritz et al. 2021) | Repeat Exposure | 185 children. | Consumption of the Novel vegetable.  Comprehensive Feeding Practices Questionnaire.  The Children’s Eating Behaviour Questionnaire.  Food Neophobia 6 item Questionnaire.  Familiarity and liking of stimuli. | The children in the control group had the lowest initial intake of daikon, at 13g which increased to 35g post-test which was significant. This amount increased to 54g at 3-month follow-up and 85g at 6 months.  For the exposure groups, those who received the grated daikon intake increased from 30g at baseline to 61g at post intervention. The intake decreased at 3 months and then achieved the highest level at 110g at 6-months post follow-up.  The children who received triangle daikon had a statistically significant increase from 26g to 69g from baseline to post intervention. This stayed elevated at 73g at 3 months and then 110g at 6 months.  The children who received daikon sticks showed a very large increase from baseline to post-intervention from 17g to 111g, there was a slight decrease at month 3 to 89g and then reached a peak at 6 months of 118g. All increases from baseline to follow-up measures were significant. |
| (Kashef et al., 2023) | Food modification | 224 children from 8 long-day care centres | Dietary intake for 5 food groups (vegetables and legumes, fruit, cereals and breads, dairy and alternatives, and meat and alternatives; all weighted). | At follow up, daily vegetable provision within intervention centres was 0.9 servings/ day (equivalent to 8.4g more per day) compared to 0.8 in the comparison group. No statistically significant difference in consumption of any particular food. |
| (Kaufman-Shriqui et al., 2016) | Parent-Child Course | 240 participants. | Nutritional Habits,  Packed Lunch Score Physical Activity and Sedentary Behaviours Evaluation of Children’s Nutritional Knowledge Anthropometric Measurements | Significantly greater increases were shown in the intervention for fruit, vegetables, habitual water drinking and decreased consumption of sugar sweetened beverages compared to the control group. |
| (Kennedy, Whiting et al. 2014) | Emotional moderation/Self-regulation/mindfulness | 6 children. | The primary dependent variable was the percentage of foods tasted each session. This was determined by dividing the number of foods that were tasted in each category by the total number of foods presented in that category and multiplying by 100.  Approach of foods was also measured to capture changes in response to foods that did not include eating but were indicative of changes in acceptance of that good. This was calculated each session by dividing the number of foods approached in each category by the total number of foods presented in that category and multiplying this amount by 100. | During ACT, there was a mean increase in the percentage of foods that were tasted for Lizzie, Eva, Petra and Monroe, by 17.8%. Ariel showed a 30.6% reduction and there was no change for Joey.  In ACT PLUS, there as an overall increase for all participants M = 47.8%, except for Monroe who showed a 19.07% reduction. Similar results are seen in the foods approached. |
| (Kong, Buscemi et al. 2016) | Education Programme | 618 children were recruited. 552 remained at the follow up. 290 children were allocated to the intervention and 263 to the control groups. | Anthropometric measures of height and weight at baseline, post intervention and 1 year follow up.  Diet was measured as a 24-hour recall per parent report at each time point. All meals and snacks consumed outside of school was recorded on a food record form.  During the school period study staff observed the children during a meal time.  Based on the 24-hour food recall, diet quality was measured with the Healthy Eating Index 2005.   Screen time was recorded by parents, | Significant HEI score changes were seen when comparing between group differences.  Components of total fruit, whole fruit, whole grain and SoFAAS.  The total HEI score remained stable among the intervention group. In the control group this HEI score decreased significantly, particularly for total fruit and whole fruit.  Scores did not change for the intervention group. There was a marginally significant increase in the intervention SoFAAS scores. And a marginal improvement in dark green/ orange vegetables/legumes. |
| (Kornilaki et al., 2022) | Education Programme | There was a total of 184 children in the intervention group and 145 in the control group. | Children were assessed using a sorting activity, practice activity and connection activity. Dietary intake was measured based on a 24-hour food and beverage intake that relates to the previous day. | At baseline the intervention group consumed significantly more healthy foods than the control group. In particular they ate significantly more fruits. This difference was sustained at time 2 but not at time 3. At time 2 there were significantly differences between group for both fruit and vegetables, with the intervention group eating more of both. |
| (Kristiansen, Bjelland et al. 2019) | Education Programme | 320 Children Allocated to the control group, 313 children were allocated to the intervention group | Vegetable frequency and Variety Vegetable amount  Direct observation of vegetable intake for two meals. | There was an increase in vegetable intake in the intervention group from 50g to 82g per day at follow-up in the direct observation. In the control group there was also an increase from 41g per day to 59 g. There was no significant effect of intervention at follow up 1 for the daily frequency of vegetable intake. In the parent reported data there was no significant effect of intervention. |
| (Kristiansen, Himberg-Sundet et al. 2021) | Education Programme | 320 Children Allocated to the control group, 313 children were allocated to the intervention group | Childrens vegetable uptake in grams/day and served vegetables in grams per day.  Perceived Usefulness was also recorded. | Where posters were perceived to be useful by teachers there was a significant increase in the amount of vegetables intake by children. Those who thought it was useful to a large degree had an additional 38g/day vegetable intake on top of this. Those who perceived the posters to be useful to some degree had a significantly smaller increase than those who did not find the posters useful. There was no impact of perceived usefulness of the 1 day course upon vegetable intake. There were no further effects of the amount of vegetables served in the kindergarten setting. |
| (Kristiansen, Medin et al. 2020) | Education Programme | Baseline n=633, Follow-up n- 596, follow-up 2 n= 567 | Measures of frequency, variety and factors potentially influencing the child’s vegetable consumption. Directions of observation of children’s fruit, berry and vegetable intakes at two meals in one day in the kindergarten, and a parental 24 h recall of the child’s intake of fruits, berries and vegetables. | No significant long-term effects in child vegetable intake were found. There was a mean difference of -0.1 times per day for the daily frequency of vegetable intake. There was also a mean difference of -0.2 different kinds of vegetables eaten over a month. There was a mean average difference of -15.0g vegetables. For measures of Daily amount of vegetables consumed there was a significant increase from baseline to follow up 1 in both the intervention and control group, this was also similar in weight at 17g. From baseline to follow up 2 there was a significant decrease of 23 g in the intervention group and a non-significant decreased of 8g of vegetables observed for the control group. |
| (Lanigan et al., 2019) | Repeat Exposure | 87 child participants | Parental nutritional knowledge Food liking-Measured using Facial display of liking by placing bowl next to face. Ranked 1-5 lowest being least liked. 0 scored for any of the 4 foods not tried Willingness to try – Scored 0-1 where 0 is not tried, 1 is licked, 2 is tried the food for each of the 4 foods at T1 and T2.   Consumption was measured using a plate waste assessment method. The amount of each of the 4 foods was measured in grams to determine how much was consumed.  Child BMI was measured at T1 and T2. | Interaction of Time of measurement and CCNP+RE was significant. The CCNP+RE food group showed significant increase in food intake compared to the RE only group.  The amount of food did not differ between T1 & T2, But children consumed twice as much CCNP+RE food at T2 compared with RE food. |
| (Larsen, Liao et al. 2017) | Education Programme | 604 students in the intervention group and 103 students in the control group. | Student survey – Child nutrition knowledge using 5 questions to classify foods into groups. 2 measuring breakfast knowledge, 2 measuring healthy snacks, and also measuring child sex and age. All food questions were picture based.  Parent survey, Children’s usual dietary intake over the previous month was recorded, and parenting feeding practices, parenting policies regarding use of food labels and parenting awareness of and satisfaction with the BHM program.  Parents completed the National Cancer Institute’s Dietary Screener Questionnaire (DSQ), which consists of 27 items to assess dietary intake.  Teachers reported the amount of engagement that each child had with the intervention. And whether they considered the intervention to be age appropriate. | Parents reported significantly increased intake of leafy or lettuce salad, beans and brown rice or other whole grains, and lower consumption of fried potatoes, and candy in the post survey compared to the pre-survey. No differences were observed for the DSQ |
| (Lattanzi, et al., 2023) | Education Programme | 115 children included in study. | Two Food frequency questionnaires administered at baseline and follow up measured children's dietary habits and physical activity. | No statistically significant variations in nuts, milk, dairy products, salami, and fruit, or legumes. Vegetable portion size was only one that significantly decreased. 76% of children at follow up increased their consumption of vegetables, from the small portions to medium or large portions (possibly due to emphasis on importance of vegetables). The reduction in children consuming medium to large portions confirms increase in consumption of right portion size. |
| (Lee et al., 2017) | Education Programme | 20 children. | Child Physical Activity, Fruit and vegetable consumption and eating in the absence of hunger was measured.  Parent practices and knowledge was measured.  And Fruit and vegetable availability in the home was measured. | At baseline children ate an average of 0.72 cups of vegetables per day, which rose to 0.77 cups of vegetables after the intervention. This increase was not significant. This was also not significant when the childcare centre was controlled for.  At baseline children ate an average of 0.99 cups of fruit per day which rose to 1.15 cups of vegetables per day after participating, again this was not significant, even when controlling for childcare centre. |
| (Leis, Ward et al. 2020) | Education Programme | 433 Control children, 462 Intervention. | Physical activity was assessed using an accelerometer.  Fundamental Movement Skills was measured using the Test of Gross Motor Development (TGMD-II) Food intake and food served was measured as servings for fruits and vegetables, grams for Fibre and mg for sodium intake. This was established through weight plate waste measures. | The intervention was not associated with significant differences in children’s food intake. There was some marginal difference in the food served following the intervention, such as there were larger portions of vegetables and fruits served in the intervention group versus the control group. |
| (Lim et al., 2016) | Education Programme | 104 in total, 52 boys, 52 girls. | Nutritional intake – based on complete food records using photos 15 nutrients and total energy measured Child eating behaviour measured using Nutrition Quotient developed by Kang et al 2012. | There were no significant differences in energy intake observed between pre and post-test for the whole scale |
| (Lumeng et al., 2017) | Emotional moderation/Self-regulation/mindfulness | 218 in HS arm, 224 in HS + POPS, 255 in HS+POPS+IYS. Total = 697 | Primary outcomes included BMI, and BMIz scores,  Age and sex,  Self-regulation- teacher assessed using Social Competence, and Behavior Evaluation (Cronbach’s alpha = .96 for preintervention and .96 for postintervention).  Three unannounced 24-hour dietary recalls were collected by phone by trained dietitians from parents regarding their child’s intake. This was conducted for 2 weekdays and 1 weekend day.  Intake at the pre-school was recorded by trained observers, members of the research team. This was conducted for each snack and meal. | There was no effect of any of the interventions on food intake. There was however a significant effect upon sugar sweetened beverage intake in the HS+POPS+IYS group, who showed a significant decline from 0.54 servings per day pre intervention to 0.47 post intervention. |
| (Maimaran & Fishbach, 2014) | Modelling TV Books Toys Characters | Study 1: 66 children in total.  Study 2: 49 children in total.  Study 3: 57 children in total. Study 4: 46 children in total.  Study 5: 52 children in total. | Experiment 1: The number of Wheat thins Crackers eaten (Maximum 15).  Experiment 2: Cracker liking using smiley scale from Unhappy to Neutral and then Happy.  Cracker similarity between Ice cream and Onion in terms of liking using a VAS scale.  Hand opening scale to describe how much the children liked the crackers. The distance between hands were measured in cm.  Experiment 3: Measured how many carrots the children plan to eat. There are a maximum of 15 wooden carrots and the children were asked to pretend they were real. If they were real how many they would eat.  Experiment 4: Grams of Carrots consumed during eating session.  Experiment 5: Grams of Wheat Thin Crackers consumed. | Experiment 1: Children in the healthy condition planned to consume the Wheat thins less than those in the yummy condition. Only 32% would choose these instead of Ritz crackers compared to 65% in the yummy condition.  Experiment 2: Children in the healthy condition ate fewer crackers than children in the control condition.  Experiment 3: Children in the read condition said they wanted to eat fewer carrots than children in the yummy and control conditions. There were no significant differences between yummy and control.  Experiment 4: Children in the count condition ate fewer carrots than children in the control condition.  Experiment 5: Those who read that crackers helped learning how to count ate less than those in the control condition. |
| (Maimaran & Salant, 2019) | Experimental | Study 1. 51 children Study 2. 51 Study 3. 56 | Consumption of favourable snack of child’s choice and less favourable healthy snack such as carrots, grapes, | Results of study 1, Children ate more carrots 33.76g on average when they were told that there was a limited supply of carrots available, compared to 21.84 g.  Results of study 2. Like study 1, this study found that in the limited condition there was larger ratio of carrot consumption to total consumption.  Children in the limited condition were also more likely to begin with eating a carrot.  Study 3. Children showed a significant liking of crackers over grapes with 74% of children choosing Crackers. In the grape limited condition, the proportion of children choosing grapes doubled from 26% to 52%, indicating that limited availability triggers a child to choose this option. |
| (Marshall, Markham et al. 2020) | Parent-Child Course | 407 parent- child dyads intervention. | Child dietary intake was measured using the Block Kids Food Screener.  Parental fruit and vegetable intake was measured using the Fruit and Vegetable National Institutes of Health self-report. Home Nutrition environment was measured to assess parental rules for limiting portion sizes, screen time, fried foods, fast food, and sugary beverages. Additionally, family dinners and rules for finishing all food on plates were measured. | Compared to baseline, child consumption of fibre at the two-year post-intervention follow-up was also increased significantly by +1.06g/1000kcal. Child consumption of fruits was significantly increased by +0.18 cups/1000kcal, and vegetables was increased by +0.14cups/1000kcal at the two-year post intervention measure.  Consumption of total fat decreased by -1.55g/1000kcal, consumption of added sugar decreased significantly, and there was a decrease of -0.52% of calories from sugary beverages. The average number of calories consumed increased significantly from baseline to follow-up by +110.72kcal, this was accounted for by child growth. Consumption of potatoes and French fries increased significantly by +0.03 cups /1000kcal. |
| (Mathias et al., 2012) | Food modification | 30 children. | Children’s Fruit and vegetable liking and preference.  Weighted Food Intake.  Demographics.  Weight Status. | Children consumed 41g more fruit in the large portion conditions compared to the reference conditions, which is about 2/5 of a serving increase.  Children consumed more of the vegetable side dish in the large portion condition by 12 grams on average.  After removing 13 of the children that ate negligible amounts of vegetables, it was found that doubling the vegetable portion resulted in a 20g on average increase in vegetable consumption.  There was no effect of increasing fruit portion size upon vegetable consumption and vice versa. |
| (Melnick et al., 2020) | Education Programme | 308 children in intervention classroom, and 215 in control. | Consumption of vegetables, edamame, cauliflower, and red peppers, as well as fruits oranges and strawberries, was measured in grams. The focus was on the vegetable consumption for this study. | Change in consumption over time varied significantly by group status, either intervention or control. Greater change in consumption was seen in the intervention group when the effect of classroom was controlled for. Significant increases in consumption of edamame, cauliflower and red peppers was observer, but not for the fruit samples strawberries and oranges. |
| (Morris et al., 2016) | Education Programme | 213 children recruited into the intervention and 180 into the control. | Children’s knowledge of healthy eating, active play and environmental sustainability of their food selection and toy selections.  Children’s food preferences, digital media viewing and physical activity habits.  Children’s anthropometry | At the follow up there was a significant difference between vegetable intake between intervention and control group. And also for the sugary drinks servings. The intervention group slightly increased their vegetable consumption from baseline to follow up and the control group decreased theirs. The intervention group reduced sugary drinks from baseline to follow up and the control group remained constant. |
| (Morris et al., 2018) | Education Programme | 168 parent child dyads in the intervention group, 132 dyads in the control group | Healthy Eating and Sustainability assessment – testing the sorting, practising knowledge, and demonstrating healthy eating and sustainability connections.  Eating and physical Activity Questionnaire. Sedentary and physical activity frequency.  Beverage consumption including water sugary drinks and flavoured milk. Nutrient poor and nutrient rich foods, measured in serves.  Fruit and vegetable servings to crease healthy food serving.  Packaged snacks, confectionary and cakes, for total unhealthy food serving. | At time point 1 the intervention group consumed significantly more healthy foods fruits and vegetables. At time point 2 the intervention group consumed slightly less unhealthy foods than the control group. At time point 3 the intervention group consumed significantly more vegetable serves that the control group, the control group showed a significant increase in unhealthy food serves between timepoint 1 and timepoint 2. |
| (Naderer, Matthes et al. 2017) | Modelling TV Books Toys Characters | 175 children | Food choice of either a piece of fruit gum or. Slice of pealed mandaring was measured.  Moderators were Child BMI, and parents’ food related mediation strategies. | In the control group where no edible product was present the children chose mandarins and fruit gums equally often.  In the candy condition, there was a significant difference between mandarin and fruit gum choice with fruit gums being chose significantly more often.  In the fruit condition, significantly more children still ate the fruit gums, which indicates that presentation of a food stimulus cannot direct towards a healthy snack.  Children’s chance of choosing the fruit gum over the mandarin in the fruit condition increased by 5.36 times compared to the control, this figure was 4.53 times in the canty condition. |
| (Natale et al., 2014) | Multiple methods | 307 Children were recruited. | National Health and Nutrition Examination Survey NHANES were adapted to measure both child food intake and physical activity levels.  Food Frequency questionnaires were reduced to 16 items to evaluate effectiveness of menu changes. | During School time the children in the intervention group decreased their junk food intake from weekly to no consumption. Fresh fruit and vegetable consumption increased 60%, Juice consumption decreased 75% and 1% milk consumption increased 90%.  For the control group the junk food increased 75% which in fact was doubled. And the water consumption decreased 70%. |
| (Natale et al., 2017) | Multiple methods | Total = 1121, Intervention = 754, Control group = 457 | Child Body Mass Index Percentile (PBMI), and parent reports of fruits/vegetables and unhealthy food. | At baseline there was a significant difference in fruit and vegetable consumption between the intervention and control group.  Consumption of fruits and vegetables was not significantly impacted by the intervention.  Stratifying for obesity status at baseline moderated the results of the effect of the intervention upon consumption of fruits and vegetables. Children who were considered obese at the baseline measures had a significantly higher increase in consumption of fruits and vegetables in the intervention group compared to the control group. In those who were not obese there was no significant impact. |
| (Nederkoorn et al., 2018) | Emotional moderation/Self-regulation/mindfulness | 66 children | Number of spoonsful between 1 and 3 that the child tastes of each dessert. | There was a main effect of the dessert type showing that overall children ate more of the smooth yoghurt with a mean of 2.18 compared to the yoghurt with pieces mean = 1.67 and jelly mean = 1.76.  There were significant interactions between condition and yoghurt type. There was a significant effect between conditions for the consumption of Jelly. Children in the exposure condition ate significantly more jelly than in the control condition. No effect of condition was found for smooth yoghurt or yoghurt with pieces. |
| (Nekitsing, Blundell-Birtill, Cockroft, & Hetherington, 2019) | Repeat Exposure | 140 Children | Weighted intake of mooli (daikon radish) at 4 time points.  Anthropometric Characteristics including height, weight and BMIz scores.  Easting status – either eater or non-eater was used as a proxy for fussiness.  Staff completed feedback surveys for intervention evaluation. | Due to many children not eating the mooli at baseline the post intervention and follow up 1 and 2 were analysed.  At the post intervention there was an interaction between the TE and NE conditions that showed that control children were significantly less likely to be eaters.  Children in the NE condition had higher odds of being mooli eaters.  There was no main effect of time on eater status.  Intake significantly increased the amount consumed during the TE condition. There was no significant effect of NE or the interaction. There was a significant effect of time showing that children ate more at follow up 2 compared to post intervention. |
| (Nekitsing, Blundell-Birtill, Cockroft, Fildes, et al., 2019) | Emotional moderation/Self-regulation/mindfulness | 267 children. | Primary outcome was celeriac intake, assessed through likelihood of children eating any celeriac, and change in weight consumed from pre-port intervention.   Secondary outcome, children’s ability to recognise the target vegetable. | The distribution of children by eating category was similar across all conditions.  In the two incongruent storybook conditions the percentage of children who ate the celeriac was consistent from baseline to post intervention at 70%.  The percentage of eaters increased from baseline to post intervention in the conditions where the storybook was congruent, with a significant increase from 69% to 83%.  Post intervention analyses indicated that the storybook conditions were slightly more likely to be eaters than non-eaters.  Sensory play had no effect upon the number of children who were eaters.  There was an interaction between celeriac and sensory play, with those who received sensory play and congruent storybooks being the most likely to eat celeriac out of all conditions. |
| (Nicklas et al., 2017) | Modelling TV Books Toys Characters | 253 preschool children in total, 128 intervention and 125 control. | Vegetable consumption As well as program feedback from the parents.  A qualitative analysis was carried out prior to the Intervention part of the study. | Children in the intervention group significantly increased consumption of vegetable dishes from baseline to follow up compared to the control group. At follow up the intervention group continued to have a higher intake compared to the control group. |
| (Nor et al., 2021) | Repeat Exposure | 172 children | DNA swab DNA swab  PROP taster status Fungiform papillae counts Food Familiarity and Liking Questionnaire. Liking of Turnip on 3 point Likert Scale.  Consumption of the Turnip in grams.  PROP taster status Fungiform papillae counts | Results revealed that overall intake significantly increased post-intervention from 14.8 ± 24.0 g to 29.8 ± 34.9 g (t (133) = − 6.17, p < 0.001). Overall liking increased significantly from 2.3 ± 0.9 to 2.5 ± 0.8 post-intervention (t(133) = − 2.35, p = 0.02). |
| (Nyberg et al., 2015) | Parent-Child Course | Total 241 Children, 129 Intervention, 112 Control children. | Primary outcomes are physical activity measured using accelerometery for 7 days. Health behaviours by parent report, including indicator foods, physical activity, sedentary behaviour, and sleep.  The eating and physical activity questionnaire (EPAQ) used to measure dietary recall in 2–5-year-olds through parents.  Self-efficacy by self-report.  Anthropometry. | At time 2 there were significantly higher servings of vegetables usually eaten in the intervention group compared to the control group. At time 3 however there were no differences between the groups. At time 3 there was a significant interaction effect for sex, boys in the intervention group showed a higher mean average serving of vegetables compared to boys in the control group. The significant interaction remained at time 2 and time 3. There were no significant findings for fruit and energy dense products. |
| (O'Connell et al., 2012) | Repeat Exposure | 96 children | The child feeding questionnaire, as well as child liking for each of a list of 8 vegetables. This was recorded by the parents as either “likes it’, “thinks it is okay’, “dislikes it”, ‘has not been offered it yet’ or ‘refuses to try it’ Vegetable consumption was observed unobtrusively and recorded from pre and post consumption weigh in sessions to the nearest gram. | There was a significant interaction of time and condition upon consumption of the three vegetables. There was an increase in preschool B which was the control school and a decrease in preschool A which was the experimental school. At baseline there was an average vegetable consumption of 10.7g at preschool A and 6.2g at preschool B. Following the intervention there was an average consumption of 8.5 g of the vegetables at school A consuming and an average of 7.5g at school B. For snow peas there was no significant change. For peppers there was no significant change for the intervention group but a significant increase in the control group. |
| (Olsen, Sick et al. 2019) | Experimental | 86 children in total | Total vegetable intake was compared for the free choice condition and the no choice condition for single stimuli and three choice alternatives.  Familiarity of vegetables and likin were also measured. | For the snack carrots there was a significantly higher intake in the no choice condition compared to the free choice. For sugar snaps and baby corn the intake was comparable in both conditions with no significant differences.  The mean total intake of vegetable was significantly higher in the free choice condition 57.5g compared to the no choice condition with single stimuli and three choice alternatives 45.6g. The no choice condition did not differ significantly from the free choice condition with a mix of two stimuli and three choice alternatives.  When comparing two free choice condition, children consumed more vegetables when eating their choice of vegetables as a single stimuli 57.5g. compared to the option of mixing two stimuli 48.2g, but the difference was not significant. |
| (Pathirana et al., 2018) | Education Programme | 640 pre intervention surveys completed, 312 post intervention surveys completed. | Physical activity levels in the previous week.  Dietary intake and screen time was measured using the Eating and Physical Activity Questionnaire (EPAQ). | Following the intervention there was an increase in the consumption of vegetables, fruits and plain milk.  There was an increase of 0.17 servings of Vegetables,  0.07 servings of fruits, and a reduction of 0.02 servings of packaged snacks, and reduction of 0.11 servings of packaged sweets.  There was also a reduction in fruit juice, cordial, soft drink, flavoured water, flavoured milk, and packaged snacks. |
| (Pinket, De Craemer et al. 2017) | Education Programme | 4968 769 Belgium 644 Bulgaria 882 Germany 825 Greece 1021 Poland 827 Spain | Food and Beverage Intake was measured using a semi-quantitative food frequency questionnaire for preschool children, which focused on the previous 12-month period. This looked at each of the food types including yoghurts and cheese, fruits and vegetables, chocolate and desserts, cookies and pastry, cereals, bread, salty snacks, meat and fish, potatoes, rice and pasta, sugar, jam and other spreads, and legumes, also focused on beverages, including plain water, tea, milk, sugared milk, fruit juices, soft drinks, light soft drinks.   Diet Quality Index – the items from the FFQ were used to compute total diet quality index and the four subcomponents. This diet quality index was developed from the Flemish active food triangle. | No significant intervention effect was found for total diet quality. For two of the subcomponents, - dietary quality and dietary equilibrium, a significant intervention effect was found. In both the intervention and control group the diet quality increased with a larger increase in the intervention group (mean difference +3.4%), compared to the control group (+1.5%). For dietary equilibrium a small increase was found in both the intervention (+0.9%) and control group (+0.2%).  When stratifying by country it was found that diet quality significantly improved in German and Greek pre schoolers. In the German intervention group there was a mean increase of 3.5% and a decrease seen in the control group of -0.2%. In the Greek sample the intervention group showed an increase of 5.6% compared to the increase in the control group of 2.5%. |
| (Piziak, 2021) | Education Programme | 148 children were recruited to take part in the BINGO game. | The goal of the study was for children to be able to recognise and unhealthful food choices, particularly sugar-sweetened beverages. | There was an increase in fruit and vegetable consumption from 17 to 20 piece, which was significant although the analyses used are not given. |
| (Ray, Figuereido et al. 2020) | Multiple methods | Total 801 participants, 441 in the control and 360 in the intervention. | Total screen time,  Total Physical activity.  Sugar consumption, sugary everyday foods and beverages, and sugary treates as times/week.  Total fruit and vegetable consumption as times per weeks. | Fruit and vegetable consumption at baseline was highest in the High PEL groups compared to other groups. There were no significant differences between intervention and control group at the follow up time point for measures of sugary everyday foods and beverages, sugary treats and fruit and vegetable. For both intervention and control groups there was a pre to post intervention increase in sugary treat consumption frequency. For the intervention group there was an increase in fruit and vegetable consumption pre to post intervention although this was not significant. |
| (Rioux, Lafraire et al. 2018) | Emotional moderation/Self-regulation/mindfulness | 70 Children | Willingness to try Vegetables.  Cognitive performance on a Categorisation task and Category based induction task.  Child food Rejection Scale. | There was a significant effect of time, Children were more willing to taste atypically coloured vegetables at time 1 compared to time 0. There was no effect of status upon the number of vegetables tried.  There was a significant main effect of condition, and a significant interaction between condition and time. Post hoc analyses indicated that at Time 0 there was no difference in the number of atypically coloured vegetables eaten in each condition, however in the simple exposure condition the number of atypical vegetables tasted at time 1 compared to t0.  At time 1, children in the simple exposure condition ate significantly more atypical vegetables compared to the diverse exposure condition. |
| (Roberts, Cross et al. 2022) | Multiple methods | 110 children | Child willingness to try was recorded on a scale of 0 to 6, scoring 1 point for each vegetable that touched their lips or tongue.  Intake was scored from 0 – 12 representing the number of pieces of the vegetable that the child consumed from a tray. | There was a significant positive trend for intake with increased number of senses explored.  Again, there were significant group differences in intake, with smell visual and smell touch visual showing greater intake of the exposed vegetables compared to the control condition.  There were no differences in intake between the visual only and the control condition. |
| (Roe et al., 2013) | Food modification | 61 children. | The number of pieces of vegetables or fruit selected by each child in the study was recorded by 2 observers seated near each table. The number of dropped pieces as well as the number of uneaten pieces were also recorded. Weight before and after the meal was recorded.  Liking of the fruit and vegetables was assessed 1 week after completion of the snack sessions, using rating scale of “yummy”, “Just okay’ and “yucky”.  Body weight and height of the child was also recorded. | Offering a variety of types of food either vegetables or fruit increased the likelihood that the children would select some pieces of their snack.  Children were more likely to select some vegetables or fruit in the snacks with variety than with a single type.  Children were significantly more likely to select some pieces of fruit rather than vegetables.  Serving a variety of fruits and vegetables also increased the number of pieces and therefore the amount consumed, which was not significantly different for fruits and vegetables. The increase in consumption from the single item to the variety of snacks condition was about 31gs, which was about 3.1 pieces extra. On average children ate more fruits (8.4 pieces) than vegetables (2.2 pieces). |
| (Roe et al., 2022) | Food modification | 53 children were included in the study. | Food consumed was weighed before and after consumption.  Energy intake was calculated from the weight consumed using information from food labels.  Assessment of Physical activity,  Height and Weight.  Assessment of food liking, assessed for 1 lunch and 1 dinner.  Children’s Eating Behaviour Questionnaire,  Caregivers Feeding styles Questionnaire,  Child Feeding Questionnaire. | Serving extra amounts of fruit and vegetables buy either addition or substitution led to increased consumption. For the vegetables the addition led to significantly increased daily intake by 12.0 g compared to the control. Vegetable intake was greater for substitution than addition with an increase of 22.0 grams compared to the control.  For fruits there was increased intake for the addition group of 60g per day compared and 69g per day for the substitution group compared to the control. Unlike the vegetable intake the fruit intake was not significantly higher in the substitution group compared to the addition group. |
| (Rollins, Stein et al. 2021) | Food modification | In total 68, 36 in the Fruit only smoothie group and 32 in the Fruit and DGV smoothie group. | Willingness to try, liking, and preference.  Child’s previous experience with the study fruits and DGVs  Smoothie Consumption measured to 0.1g using pre and post consumption weighing sessions.  Anthropometrics.  Children’s Behaviour Questionnaire  Children’s Eating Behaviour Questionnaire.  Child’s intake of DGVs and Fruit using a food frequency questionnaire.  Child Feeding Questionnaire. | Prior to the study, children on average ate the Dark Green Vegetables, Spinach, collards, Kale, less than once a month on average.  Only 21.9% of parents reported that their child consumed spinach once or more in the past week.  Only 18.6% of the children in the Fruit and DGV condition reported that they had previously tried a smoothie that was called a Green Smoothie.  During the ad libitum intake session children consumed 1.6 portions of the Fruit and DGV smoothies on average. When the children’s preferred Fruit + DGV smoothie was served during the ad libitum snack session they consumed an average of 225.7g, this ranged from 0 g to 607.0g. Within the Fruit and DGV condition the children consumed an average of 18.3g of DGV, which is 0.7 of a cup.  Children’s eating responses did not differ by smoothie condition. The willingness to try was similar to those in the Fruit only condition. |
| (Savage et al., 2013) | Food modification | 46 children 12 children were excluded due to various reasons | Vegetable Familiarity,  Vegetable Liking – measured as “yummy”, “just ok”, or “yucky” Vegetable willingness to taste Vegetable Consumption.  Dip liking using same procedure as vegetables. | In experiment 1, children were more than twice as likely to reject the vegetable when it was alone compared to with a plain dip (OR = 2.21, p < .001).  They were also three times more likely to refuse the vegetable alone compared to with a herb dip (OR = 3.43, p< .001). Rejection did not differ between dip types.  In experiment 2, children ate more of both celery (p <.05) and squash (p <.05) with the dip than without. The amount of celery that was consumed increased by 62% when paired with the herb dip, and squash increased more than twice as much. Dip type had no effect on amount eaten. |
| (Serebrennikov et al., 2020) | Education Programme | 135 students recruited, 98 remained following exclusion criteria.  62 were in the intervention group and 36 participants in the control. | Amount of fruit and vegetables selected on their lunch plate.  Amount of fruit and vegetables wasted by the students. | Food selected and food wasted was higher in the control group compared to the intervention group during the intervention period. The difference is not statistically significant. The classroom nutrition programme had no effect on the food selected and the food wasted by students in the treatment group in school lunchtimes.  Treatment students selected more fruits and vegetables compared to controls; however, they also wasted more fruits and vegetables. Indicating that they are not actually consuming the fruits and vegetables on their plates. Unintended effect of dietary interventions is the greater selection and waste of fruits and vegetables. |
| (Sharma et al., 2016) | Parent-Child Course | In total 717 children participated in the study. 407 in the intervention group and 310 in the control. | Child anthropometrics, BMI, height.  Child dietary intake – parent reported block kids food validated Food frequency questionnaire – measured as number of servings.  Parent intake of fruits and vegetables – 10 item screener.  Parental food practices rules and mealtime environments – Self report questionnaire.  Parent and child demographics. | Those in the brighter bites intervention group had a significant increase in cups of fruits and vegetables compared to the control group post intervention. There was also a significant decrease in added sugar consumption in the intervention group pre-to post intervention. There was an increase in fibre intake from baseline to midpoint but not to post intervention in the intervention group but not in the control group. There was a nonsignificant decrease in caloric intake in the intervention group. |
| (Smethers, Roe et al. 2019) | Food modification | 46 children in total. | Food consumed was weighed before and after consumption.  Energy intake was calculated from the weight consumed using information from food labels.  Assessment of Physical activity,  Height and Weight.  Assessment of food liking, assessed for 1 lunch and 1 dinner.  Children’s Eating Behaviour Questionnaire,  Caregivers Feeding styles Questionnaire,  Child Feeding Questionnaire. | Increasing the portion size of all foods and milks served over 5 days led to significant increase in the mean daily weight consumed. Serving larger portions increased the mean intake by 143 g per day. The difference between the 2 conditions was 154 g per day. And the effect of the portion size upon intake remained significant.  There was also a significant increase in energy intake in line with larger portion sizes of all foods and milk over 5 days, of 167 kcal per days, which was also significant between the two conditions at 186 kcal more.  When larger portions were served there was a 3.5% increase in the energy density of the foods that was consumed.  Portion size increases also showed increased amount of fruit and vegetables consumed as snacks and also fruit as part of the meal but not for vegetables. |
| (Smith et al., 2013) | Parent-Child Course | 440 Children and families | Primary outcome is BMI z score Secondary outcomes are:  • BMI • Waist Circumference  • Waist Circumference z score • SDQ outcomes • Parenting self-efficacy • Physical activity & sedentary behaviour  • Proportion of Parents and Children eating greater than or equal to 5 portions of fruit and vegetables daily. | There was a significant increase in both parents and children who ate 5 or more portions of fruit and vegetables per day. There were no gender differences in this result. |
| (Staiano, Marker et al. 2016) | Modelling TV Books Toys Characters | 42 participants | Pre and post consumption was measured using weighed modelled food and a comparison food, these were green bell pepper and dry cereal respectively.  Pre and post intervention dietary habits were recorded from the parents. This included a 12-item inventory of the fruits and vegetables that the children consumed. The children themselves reported their enjoyment of the modelled food and what they thought of eating it in the future. | There was a significant difference in the amount of bell pepper consumed at day 7, after controlling for the amount of bell pepper consumed on Day 1. Children in the F&V group ate on average 15.5g more bell pepper than those in the no DVD condition. At baseline children in the F&V DVD group initially consumed significantly less of both dry cereal and green bell pepper during the day 1 visit. There were no significant effects of parental observation. |
| (Steenbock, Buck et al. 2019) | Education Programme | Intervention group was 440 at baseline and 335 at follow – up .  Control group was 391 at baseline and 306 at follow up. | Anthropometric data, height, weight, body composition.  Motor Skills, Screen time and physical activity was measured,  Healthy eating was assessed as the consumption of unsweetened beverages, fruits and vegetable snacks and the number of meals per day, during the last week in a self-developed FFQ.  Family health climate was also measured. | Only 12% of the children in the intervention group and 9% of the controls in the day care facilities consumed the recommended number of fruits and vegetables per day (5) at baseline compared to 13% and 11% at follow up. 50% of the children consumed at least four glasses of unsweetened beverages per day at intervention and 49% at control. This proportion changed to 47% intervention and 51% control following the intervention. There was no change in the proportion of children that were compliant with the recommendation for snacking. Children at the intervention daycare facilities had a 66% significantly lower chance to be compliant with the recommendation for unsweetened beverages compared to controls. |
| (Suarez-Balcazar et al., 2014) | Education Programme | The target school had a total of 90 children in the kindergarten and first grade. The comparison school had 120. `There were a total of 75 children pre-survey and 82 post survey participating in the target school. The comparison school had 116 participants pre and 108 post | Researchers collected 30 days’ worth of observation during lunch time.  Food choice was measured as those who used the salad bar to either pick a starter or a side dish. Total food choice/selections available was catalogued for each lunch time. | There were significant differences in choice of salad items pre and post education in the Target school. Choosing salad significantly increased for both boys and girls following education in the target school. At the comparison school there was no change in choosing of salad. Furthermore, there were significant differences between the target school and comparison schools Fresh fruit consumption significantly increased at target school but decreased at comparison school. In the comparison school the choice of salad as an entrée increased but as a side dish it decreased. |
| (Tani et al., 2021) | Other | Total 7970 children 2307 in 2015,  2898 in 2016 2765 in 2017 | Children’s eating behaviours Body Height  Body Weight  Frequency of eating vegetable dishes | School level promotion significantly increased better eating behaviours.  Schools who had a higher proportion of children who ate vegetables as their first bite also ate significantly more vegetable dishes.  They were also more willing to try more dishes.  They also had significantly higher variety of vegetables that they ate.  Odds of trying vegetables on own was 1.5 times higher in girls compared to boys.  Odds of consuming a wider variety of vegetables was 1.3 times more likely in the 5-year-old class compared to the 3-year-old. |
| (Toossi, 2017) | Experimental | 29 participants and their parents were recruited. 23 Participated in the field experiment and were included in the study | Changes in dessert choice.  Proportion of fruit cups chosen.  Child behaviour of fruit preferences. | 95% of the time the entire dessert cup was consumed at every time point by all children. Fruit cup was chosen for 50.85% of the time. Cookie cup for 46.65% and neither desert was chosen 2.33% of the time.  There was an increase from 32% to 81% in week 2 choosing fruit cups. This then decreased to 64% in week three and further to 29% in week 4. Pre to post intervention choices showed no change, as there is relatively little difference between the numbers choosing fruit cups in week 1 and 4. |
| (Toussaint et al., 2021) | Education Programme | 115 teachers, 249 children | Teacher and Child Characteristics.  Teacher’s knowledge, attitude, practices, and level of confidence.  Teacher’s and children’s BMI and body composition.  Teacher’s and children’s dietary intake and physical activity level. | No effects of the intervention upon the dietary intake of either children or teachers were observed |
| (Van Stokkom et al., 2018) | Food modification | 70 parent-child dyads. | Consumption frequency of vegetables,  Consumption frequency of target vegetables.  Child Food Neophobia Scale.  Child liking of the purees included, using a hedonic facial scale. | There were no overt measured of fruit or vegetable consumption in this study relating to the vegetable purees. |
| (Vandeweghe et al., 2016) | Modelling TV Books Toys Characters | 204 Children | Behavioural Inhibition System and Behavioural Approach Scales.  Vegetable Liking List Child Food Neophobia scales, 6-item questionnaire.  Hunger Rating Scale | The reward group were significantly more likely to taste immediately and after hesitation compared to the control group.  Modelling also showed an almost significant result of willingness to try foods when compared with the control group. |
| (Vandeweghe et al., 2018) | Experimental | 77 in each location. 154 in total. | Behavioural inhibition and behavioural activation.  Vegetable liking of 10 vegetables including fennel, chicory, zucchini, mushrooms, peas, leek, Brussels sprouts, beetroot, spinach, and cauliflower.   Consumption and tasting of chicory.  Reward sensitivity. | There were no significant differences in consumption between the four conditions at baseline.  There was a significant difference in consumption when examining the interaction between time and condition. At both post-test and follow up the control group showed significantly lower consumption compared to all three strategies used. There are no significant differences between the strategies. |
| (Vaughn, Hennink-Kaminski et al. 2021) | Modelling TV Books Toys Characters | 853 children in total | Children’s diet quality, Dietary intake on two weekdays and one weekend day using observation and parent completed food diaries.  Children’s physical activity using accelerometers.  Children’s anthropometrics.  Parent practices through surveys.  Childcare centre practices. | There were no significant differences in change from baseline to post intervention between intervention and control arms in the HEI scores. There was a significant increase in the sodium component score in favour of the intervention arm of this study. |
| (Vitale & Coccia, 2022) | Education Programme | 51 participants (22 in intervention and 29 in control group). | Fruit and vegetable intake measured via food frequency questionnaire. Nutrition knowledge measured with modified food knowledge questionnaire. Fruit and vegetable liking measured measured on hedonic scale at baseline and post-intervention. | Increase in vegetable intake was statistically significant in the intervention group but was statistically significant between intervention and control group. Consumption of fruits was significantly increased in both groups. |
| (von Nordheim et al., 2022) | Modelling TV Books Toys Characters | 172 children. 103 were included in the intervention and 69 were in the control. | Monitoring sheets were used to record child’s food intake from the buffet, food intake was recorded and later converted to caloric value using product information provided by the manufacturer. | The mixed ANOVA for the overall model showed that the main effect of time and the main effect of condition were not significant, however, the interaction between condition and time was significant.   Paired t-tests showed that the healthy food advertisement group significantly increased item consumption from 6.2 items at T1 to 9.5 items at time 2 and increased caloric consumption from 90.3 at T1 to 119.0 at T2.  The control group significantly decreased item consumption from T1 (7.6) to T2 (5.9). the change in caloric intake was not significant for the control group.  Independent t-tests showed that T1 intake did not significantly differ between groups. |
| (Whiteside-Mansell et al., 2021) | Education Programme | 267 parents were interviewed at enrolment and 165 in follow up home visits. 161 parents were interviewed at both timepoints. | Carotenoid level scores.  Fruit and Vegetable consumption was measured using Food Frequency Questionnaires targeting the WISE foods. The WISE foods including tomatoes, sweet potatoes, carrots, bell peppers, spinach, greens, green beans, apples, strawberries and blueberries. | Parent report of child consumption showed that there was a significant increase in consumption of the WISE foods from baseline to follow up. Preschool children increased from more than 2-3 times a month to once a week, and elementary school children increased in a similar way. When examining the fruits and vegetables separately, it was seen that there was a similar increase in both from baseline to post intervention. |
| (Williams et al., 2014) | Education Programme | 1143 respondents, 552 in the intervention group and 591 in the control group | At-home consumption of fruits, vegetables, and milk through survey.  Questions were adapted from the National Health and Nutrition Examination Survey 2005-2006, the California Cooperative Extension Food Behavior Checklist. | The program did not have a significant effect on the daily consumption of fruit. For vegetables the mean number of cups eaten per day at home increased from baseline to follow up by a mean of 0.12 cups per day.  There was no significant difference in the mean average of combined fruits and vegetable consumed, likely due to the non-significant effect on fruit consumption.  There was a significant increase in the number of days that children-initiated vegetable snacking by 0.3 days, which is an additionally 1 day in a 5-week period. |
| (Willis et al., 2014) | Parent-Child Course | 77 participants were recruited, 71 agreed to complete the survey,  60 participants completed the pre and post course questionnaires. | Eating behaviours,  dietary intake,  parentally self-efficacy. | In children the frequency of cooked vegetables, fresh fruit, and baked beans/lentils/chickpeas all increased following the course. The increase in baked beans and fruits persisted to the 1 year follow up. The increase in salads/raw vegetables did not reach significance (p=0.011), although this is only marginally. There was a marginal reduction in cakes/biscuits p=0.028. No significant changed in beverages. When excluding children at weaning age, the significant result for increased fresh fruit consumption was no longer significant at the follow up. |
| (Witt & Dunn, 2012) | Emotional moderation/Self-regulation/mindfulness | 263 children | Process and outcome evaluation. Consumption of fruit and vegetable snacks | Children who received CMH significantly increased their consumption of fruit snacks by 20.8%, and vegetable snacks by 33.1% between baseline and 3 months following completion of the program. The hierarchical lineal modelling determined that it was only condition (control vs CMH) that accounted for fruit and vegetable consumption. |
| (Yoong et al., 2020) | Education Programme | At baseline 522 children were recruited to have height and weight measured. 500 consented to dietary observations and 460 consented to complete a survey. These children were allocated across 35 childcare centres that consented to take part. | 1. Number of servings of the 5 core and discretionary food groups 2. Childcare educator-reported child diet quality 3. Child BMI z scores  4. Child HRQoL  5. Child diet outside of care | At the 12 month follow up children attending intervention childcare centres consumed significantly more fruit and dairy portions and significantly fewer discretionary items, servings of bread and cereals compared to the control group.  There was no significant difference between control and intervention for the consumption of vegetables and meat/meat alternatives. |
| (Youssef et al., 2022) | Emotional moderation/Self-regulation/mindfulness | 98 children took part. 46 in the experimental condition, and 44 in the control condition. | Questionnaires pertaining to child's involvement in cooking at home, eating habits, and food neophobia scale. Subjective hunger was measured before and after the session using teddy the bear scale. Portions of selected foods were weighed out before and after the session to determine how much the child had consumed. Liking of food was determined on a 5-point hedonic scale ranging from awful (1) to brilliant (5). | No differences reported between groups with total consumption, consumption of familiar item, or unfamiliar items, with neophobia as covariate. When looking at individual food items, differences were observed for spaghetti (less consumption in experimental group with medium effect size), omelette (experimental group consumed more pepper omelette than control with medium effect size). |
| (Zeinstra et al., 2017) | Modelling TV Books Toys Characters | 99 children aged 4-6. | Vegetable intake and vegetable choice. Intake was calculated by subtracting the leftover weight from the weight before consumption.  The Child Eating Behaviour Questionnaire was completed as well as the 6-item Food neophobia Scale.  Parents also completed questionnaires about their child’s liking of carrots in boiled and raw form as well as their preference of raw vegetables and liking of 17 Vegetables (9 raw and 8 boiled vegetables). | There were no significant differences between conditions for the intake of carrot during session. There was wide variation in the amount of carrot consumed by children during the first session ranging from 19.8g of carrot to 30.7.  For all three conditions the amount there was no significant difference in the amount of carrot eaten between S1 and S8. This indicates that there is no increase in carrot intake during the intervention period.  At the 9 month follow up period there was a significant difference between the carrot intake from S1 to follow-up. Those in both the Convivial eating and the Convivial eating with positive restriction groups, who both were in the TV idol intervention group, differed significantly from the control condition, showing there was a significant increase in carrot intake from S1 to follow up in both groups. There was, however, no significant difference between the two TV Idol groups. The control condition did not have a significant change in the amount of carrots eaten from S1 to follow up. |
