# Supplementary material for: Improving Children’s Diets by Introducing Fruits and Vegetables in Group-Based Settings: A Scoping Review
Source: Nutr Rev. 2025 Jul 3;84(5):1039–50. doi: 10.1093/nutrit/nuaf092 (PMC13075485; doi:10.1093/nutrit/nuaf092)
Supplement: nuaf092_Supplementary_Data [file nuaf092_supplementary_data.zip › Supplementary Table IV. Additional Study Characteristics.docx]

Supplementary Table IV. Additional Study Characteristics.

| **Study** | **Journal** | **Country of origin** | **Local setting** | **Aim** | **Study/Intervention duration** | **Study Population** |
| --- | --- | --- | --- | --- | --- | --- |
| (Adab et al., 2018) | The British Medical Journal | UK | West Midlands | To report the results of the West Midlands ActiVe lifestyle and healthy Eating in Schools study (WAVES). | 12-month intervention with a daily additional 30-minute physical activity opportunity. Six-week interactive skill-based programme | Children aged 5 to 9 included in study. 52.7% males, generally less deprived households. More likely to consume 5 portions of fruits and vegetables and more likely to achieve at least 60 minutes of moderate to vigorous physical activity. |
| (Ahern et al., 2019) | Appetite | UK | West and South Yorkshire | To test the hypothesis that offering pre-school children repeated exposure to moderately familiar vegetables in the form of variety would produce greater overall vegetable consumption. | 3-week exposure, 10 weeks in total. | Children aged between 24 and 60 months old. Mean age 43.44 months (SD = 0.87) 53% male, with average BMI z score of 0.85 (Sd = 0.15) |
| (Anzman-Frasca et al., 2012) | Appetite | USA | Pennsylvania | To assess two approaches to increasing pre-school children’s liking and intake of vegetables. | 12 weeks | Children aged 3 to 6 years old. Experiment 1: 49% Male, 83% White, 4.7 years old (SD = 0.8), 24.4% overweight.  Experiment 2: 61% Male, 80% While, 4.4 years old (SD = 0.8), 13.9% overweight. |
| (Bai et al., 2018) | Nutrition Research and Practice | South Korea | Cheogju | To explore potential determinants of vegetable consumption in Korean children that can be used as target variables for intervention programs. A second aim was to assess whether the Veggication program increases vegetable consumption. | 4 weeks | 36 boys and 35 girls, 62% with working mothers, 81.7% came from homes where the mothers would prepare the meals. Demographics were similar between intervention and control groups. |
| (Belot et al., 2016) | Journal of Health Economics | UK | England Wide | To investigate how to incentivize school age children to consume healthier food. | 6 months. | Children in school year 2 (aged 6-7 years old) and Children in school year 5 (aged 9-10). |
| (Boyer et al., 2012) | International Journal of Behavioural Nutrition and Physical Activity | USA | West Lafayette | To evaluate the effect of serving healthy high-fibre snack foods in normal or shaped form on preschooler’s snack consumption. | 9 weeks. | Children aged between 2-5 years old were recruited, in total 11 boys and 10 girls completed the study. The sample was 62% Caucasian and 38% Asian. |
| (Braga-Pontes et al., 2022) | Public Health Nutrition. | Portugal | Leiria Districs | To test the efficacy of three nutrition education strategies on the intake of five different vegetables in preschool children compared to the Portuguese Food Wheel Guide | 5 weeks | Across all groups there were similar numbers of males and females. 51.2% of the total were Male. Child mean age was 4.63 years (SD = 0.977) |
| (Brennan et al., 2021) | International Journal of Behavioural Nutrition and Physical Activity | Ireland |  | This paper reported on the trial outcomes for Project Daire, that aimed to improve primary school children’s knowledge of and interest in food to improve health related quality of life and wellbeing. | 6 months – two intervention. | Approximately equal numbers of males and females were recruited across 4 conditions. The Nourish-No Engage group had equal numbers of rural and urban schools. The Engage -No nourish was significantly higher in urban schools |
| (Bucher Della Torre et al., 2015) | Sage Open Medicine | Switzerland | Geneva | To conceptualise and pilot test a programme of three workshops aiming to prevent the development of overweight in susceptible preschool children | Three workshops lasting 2 hours 30 minutes each. 4-6 month follow up. | Children aged 3-6 years old, mean 3.4 years SD = 0.6. 11 girls and 7 boys participated. Six children had both parents within BMI normal range, 6 children had one parent suffering with overweight or obesity, and 5 children had both parents suffering with overweight or obesity. |
| (Capaldi-Phillips & Wadhera, 2014) | Research and Practice Innovations | USA | Arizona State | To compare the effectiveness of associative conditioning to exposure on changing liking for a bitter and non-bitter vegetable in children. | Two weeks. | Pre-school aged children aged 3 to 5 years were recruited 13 boys and 16 girls Mean age is not reported. |
| (Carney et al., 2018) | Physiology & Behaviour | USA | Pennsylvania | To test the hypothesis that adding multiple herb and spice blends to vegetables to increase flavour variety within a meal would increase vegetable intake in 3–5-year-old children. | Two laboratory visits | Children aged 3-5 years, mean average 54.2 months (SD = 8.2). 56.85 male. 93.2% White, 79.5% not overweight. |
| (Carstairs et al., 2018) | Nutrients | UK | Fife and Tayside | To investigate the effects of downsizing combined with variety of food intake in pre-school children. And exploration of the influence of child eating behavioural traits on consumption. | 8 weeks | 3-5 year old pre-school children  Mean age 3.9 years (girls) and 4.0 years (boys)  Mean BMI 16.9 (girls) and 16.0 (boys) % overweight 47.8% girls and 0% boys |
| (Chen et al., 2014) | Appetite | USA | Northern California | To evaluate the short-term impact of the pilot intervention on participants’ knowledge, attitude, and behaviours related to the featured produce. | 3 months | Intervention group consisted of 604. 50% Latino, the remainder were primarily white. 35% kindergarteners, 37% first graders, 28% second graders.  Control group demographic information not available. |
| (Choi et al., 2018) | Nutrition Research and Practice | South Korea | Seoul | To investigate the association between the provision of nutritional and emotional and behavioural outcomes in preschoolers. | 8 weeks | The mean age of the children was 4.97 +- 0.67 years, 53.4% were boys. Most parents were college graduates. About 2/3 of the children’s fathers were workers or owner-operators and about 1/3 of the mothers were unemployed. |
| (Correia et al., 2014) | Childhood Obesity | USA | Connecticut | To test the feasibility of two strategies to increase pre-schoolers’ vegetable consumption and willingness to try vegetables. | 1 day | There were more boys in this sample by over double compared to girls 29:14. The mean age was 4.4 years (SD = 0.6). BMI percentile was 57.3 (SD=28.1). 16.4% of the children were overweight or obese. 41.1% were non-hispanic black, 37.5% were non-hispanic white, 14.3% were Hispanic, and 7.1% were Asian. |
| (Coulthard & Ahmed, 2017) | Food Quality and Preference | UK | Leicester | To compare the efficacy of difference sensory tasks in encouraging novel food acceptance in school children. | The study was conducted within on day at the school | Children aged between 4 and 8 years old (mean age 5.35 years)  53 males and 59 females. |
| (Coulthard & Sealy, 2017) | Appetite | UK |  | To examine whether a creative multi-sensory play game using fruits and vegetables carried out prior to a tasting session, can influence tasting behaviour in young children. | A single half-day session | There were 35 female children and 27 male children, aged mean 3.36 years (SD = 0.52 years). Mean daily fruit and vegetable portions consumed at baseline =3.43 (SD = 1.78) 98% white British. 24% consuming 5 portions of fruit and vegetables per day. |
| (Crespo et al., 2012) | Annals of Behavioral Medicine | USA |  | To evaluate the impact of a multi-level promotora-based (Community Health Advisor) intervention to promote healthy eating and physical activity and prevent excess weight gain among Latino children. | Some elements were 7 months, others up to 3 years. | Predominantly Latino study sample, children with a mean age of 5.9 years (SD = 0.9) 50% girls, 17% overweight 29.5% obese. |
| (De Bock et al., 2012) | Public Health Nutrition | Germany | Baden-Württemberg. | To assess the short-term impact of a nutritional intervention aimed at reducing childhood overweight in German pre-school children. | 6 months | Children aged between 3 – 6 years old. 53.2% males, with a mean age of 4.26 years (SD = 0.78). |
| (De Coen et al., 2012) | Public Health Nutrition | Belgium | Flanders | To examine the effects of a 2-year multi-component intervention in local communities with different socio-economic characteristics on the prevention of overweight among 3-6 year olds. | 2 years | Children aged 3-6 years. |
| (de Droog et al., 2014) | Appetite | The Netherlands | Nijmegen | To investigate whether picture books can stimulate young children’s vegetable consumption. | 5 days. | Aged 4-6 years. Most children were classified as normal weight (81%). 51% were boys. Only schools without formal fruit and vegetable programmes were selected. Varying socioeconomic status and cultural backgrounds were selected. |
| (DeJesus & Venkatesh, 2020) | Pediatric Obesity | USA | North Carolina | To examine the relative effectiveness of showing vs telling children about food to promote healthy eating with a focus on unfamiliar foods and vegetables. | One day | Children aged 3-6 years old, with a mean age of 59.77 months (SD = 12.30), 54.9% female, 70.4% Non-Hispanic White, 12.7% food insecure. |
| (DeJesus et al., 2019) | Journal of Experimental Psychology: General | USA | Midwestern region. | To better understand the early psychological impact of brief health messages on Children’s eating behaviour | 1 day | Children with a mean age ranging from 5.93 in study 1 to 8.98 in study 6 were recruited. Although predominantly children aged 5-6 years were recruited, study 6 focused on 8-9 year olds.  There were equal numbers of males and females in most groups. Children were predominantly White. |
| (Dial et al., 2020) | Early Childhood Education Journal | USA | Rural Town – Northwest Ohio | This study aimed to incorporate mindfulness into an approach including taste exposure modelling and sensory awareness to reduce neophobia | 10 sessions over 5 weeks | Pre-schoolers aged 3-5 years, 60 % male, 96.9% Caucasian, $80000 or higher income. |
| (Diktas et al., 2021) | Appetite | USA | Pennsylvania, | To test the effect on vegetable intake of either serving a larger portion of vegetables, enhancing their flavour with small amounts of salt and butter, or combining these strategies at a meal. | 4 weeks. | 3- to 5-year-old preschool children were recruited. The mean age of the children was 4.2 years (SD = 0.6) 16% of the children were classified as having overweight. 77.3% of the sample were white and 75% had a household income over $50,000 |
| (DiSantis et al., 2013) | Pediatrics | USA |  | To experimentally evaluate the effects of dishware size on young children self-served portion sizes and energy intakes at school lunch. Secondly, to identify characteristics of children who responded to larger dishware by serving more food. | 9 weeks | There was a mean average of 2.2 children in participating households, 20% experienced food insecurity.  88% were African American non-Hispanic, 61% female, 45% overweight or Obese. |
| (Edwards et al., 2022) | Appetite | UK | Birmingham | To examine the effect of adults’ Facial expressions whilst eating raw broccoli on children’s acceptance and intake of a typically less preferred vegetable. | One experimental session. | Parents mean age was 37.1 years, they were 93.7% white. Parent highest education level was predominantly undergraduate degree 40.5%) and post graduate degree (44.1%) Children had a mean age of 5.5 years, with 64 males and 47 females taking part. Child sex did not differ between conditions. |
| (Elrakaiby et al., 2022) | Public Health Nutrition | USA | Lincoln Nebraska | To determine whether the ‘Read for Nutrition’ programme would increase liking and consumption of broccoli (a target vegetable) in preschool children and test acceptability and practicality of the programme. | Two weeks teacher training, 3 weeks children reading programme. | Children with average age 4.26 (SD = 0.68) years. 56.5% female, 49.3% Non-Hispanic White, 31.9% Non-Hispanic Black. |
| (Farrow et al., 2019) | Appetite | UK | West Midlands, preschools and primary schools | To explore the impact of playing on the app on children’s liking and intake of vegetables in comparison to a control. | 1 day | Children aged 3-6 years Mean = 4.38 (SD = 1.06) 50% male/female |
| (Fisher et al., 2012) | Journal of the Academy of Nutrition and Dietetics. | USA | Houston Texas | To determine whether repeatedly offering a moderately-liked raw vegetable with a familiar dip influenced liking and intake of that vegetable among bitter-sensitive and bitter-insensitive preschool-aged children. | 7 Weeks | The children were 52% boys from predominantly Hispanic families, 88%, with a mean age of 4 years. 21% had a BMI above the 95^th^ percentile. Mean food neophobia among children was 37. 70% were bitter sensitive. |
| (Garcia et al., 2020) | Nutrients | UK | North Lanarkshire Scotland | To evaluate the effect of a cooking skills programme (The Big Chef Little Chef (BCLC) with tandem participation of parents/carers and their 3-5 year old children in child food fussiness and willingness to try green vegetables. | 4 weeks 1.5 hour weekly sessions | Parent/carer – child dyads 69% attended all sessions – 31% attended 3 sessions. 89.1% female intervention group 93% female control group. Child sex 56.3% female in the intervention group 57.9% female in the control group |
| (Gomes et al., 2018) | Public Health Nutrition | Portugal | Lisbon | To conduct a pilot test of the effectiveness of the Red Apple programme a brief parental school-based intervention to promote healthy eating behaviours in their young children and to assess the impact of the programme of behavioural measures and parental cognitive dimensions previously associated with parent’s motivation to engage in positive changes related to the child’s eating patterns. | 1 year in total. | Children aged 3-6 years of age. Parents were predominantly aged between 25-44 years. With college education, and both parents present in the household. |
| (Gripshover & Markman, 2013) | Sage Publications | USA | Preschool affiliated with Stanford university | a) Analyse the incipient knowledge that guides young children’s reasoning about the food-body relationship b) Identify conceptual prerequisites for understanding food as a source of nutrition c) To teach new body of theory of food | Up to 12 weeks up to twice a week | Children aged between 4- 5-year olds, Mean age = 4.7 years (SD = 0.36) 31 female |
| (Halbeisen & Walther, 2021) | Appetite | Germany | Three German Cities | To investigate how healthy eating in preschool children could be promoted by means of an associative conditioning procedure with non-food stimuli | Intervention - associative conditioning | Children aged 3-6 years for both experiments.  Experiment 1 consisted of 40 children (24 boys) with a mean age of 4.9 years ranging between 39-80 months.  Experiment 2 consisted of 42 children (17 boys) with a mean age of 4.5 years ranging between 38-75 months. |
| (Harnack et al., 2012) | International Journal of Behavioural Nutrition and Physical Activity. | USA | Minneapolis | To evaluate the independent effects of the following meal service strategies on intake of fruits and vegetables of preschool children, 1) Serving fruits and vegetables in advance of other menu items as part of traditional family style meal service, and 2) Serving meals portioned and plated by providers. | 6 weeks | Children aged 2-5 years, 75.5% African American, 24.5% obese, 11.5% overweight. Parents or guardians 49.1 had some college or associate degree, 41.5% were high school graduates. |
| (Hoppu et al., 2015) | Food & Nutrition Research | Finland | Hanko | To evaluate the effect of sensory-based food education activities on children’s willingness to eat samples of selected vegetables and berries. | 5 weeks, one 20–30-minute session per week. | Children aged 3-6 years participated from two kindergartens. The intervention group was 50% girls, the control was 58%. Mean age of children was 5.1 years in the intervention and 4.7 in the controls. Maternal age of the intervention children was 34.1 years and 33.0 years in the control. 74% of mothers worked in the intervention and 77% in the control.  23% were university or polytechnic, educated in the intervention and 32% in the control. |
| (Hughes et al., 2012) | British Journal of Nutrition | UK | Across England | To explore geographic and demographic variations in the uptake of School Fruit and Vegetables Scheme and the amount of fruit and vegetables that children consume on a daily basis. | The free piece of fruit and vegetables runs every day for the first three years of school. | School aged children across the UK aged between 6-7 years old. The geographic location was across the whole of the UK, and the deprivation was analysed across 10 levels. |
| (Johnson et al., 2019) | International Journal of Behavioural Nutrition and Physical Activity (IJBNPA) | USA | Rural Colorado, 2 mountain, 2 plains | Primary aim to establish whether The Food Friends-Fun with New Foods effectiveness is sustainable over time. Secondary to determine whether improvements in food hedonics would be associated with changes in consumption of target foods during typical eating occasions of the school day | 12- week intervention Total of three years including follow ups. | Children aged 3-5 - English and Spanish speaking, 41% Hispanic, 67% low income, 2 mountain and 2 plains rural communities. Parent confirmed intellectual disabilities and allergies excluded. Intervention and control sites matched on community statistics. 54.4% female, 29% overweight or obese |
| (Jones et al., 2015) | Implementation Science | Australia | New South Wales | To evaluate the effectiveness of an intervention to increase the implementation of healthy eating and physical activity policies and practices by centre-based childcare services. | 12 months | The children were predominantly from the bottom 50% of New South Wales SES. Distributed evenly between urban and rural locations. The children were enrolled in either Pre-School or Long Day-care service. |
| (Joseph et al., 2015) | Childhood Obesity | USA | Conneticut | To determine the impact of a short-term nutrition education pilot intervention on preschool-age children’s snack food choices. | 2- week duration 9 x 30-minute lessons. | Children aged 3-5 years, all from the same child-care setting. 62.2% of the children were female. The mean age in the intervention group was 43.2 months and in the control group it was 51.9 Months. |
| (Karagiannaki, Ritz, Jensen, et al., 2021) | Foods | Denmark | Copenhagen | To examine the effect of different repeated exposure frequencies on fruit and vegetable acceptance using a novel vegetable, daikon, among 3-6 year old children. | 7 exposures either once a week, twice a week, or once every second week with 3- and 6- month follow ups. | Children aged 3-6 years old. Mean age varied for each of the 4 groups, with a mean of 51.8 months for the control, 55.0 for the 2/ week, 53.8 months for 1/week and 53.8 for the every second week groups.   There were also more boys than girls in the control group and 1/week groups, but more girls than boys in the 2/week group. There were even number in the once every 2 week group.   There were also more boys than girls in the control group and 1/week groups, but more girls than boys in the 2/week group. There were even number in the once every 2 week group. |
| (Karagiannaki, Ritz, Andreasen, et al., 2021) | Foods | Denmark | Copenhagen | To examine the impact of serving style on liking and intake of a novel snack vegetable. | 4-week intervention 3- and 6- month follow ups. | Children aged 3-5 years old. Mean age varied for each of the 4 groups, with a mean of 51.75 months for the control, 55.02 for Grated beetroot and daikon, 52.78 months for Triangle shaped and 53.77 for stick.   There were also more boys than girls in the control group and the stick group but more girls than boys in the other groups. |
| (Kaufman-Shriqui et al., 2016) | Nutrients | Israel, Europe and former USSR, Ethiopia and East African, and thers. |  | To enhance children’s nutritional knowledge and to promote their mother’s adoption of healthy feeding habits, while considering food affordability constraints. | 15 weeks with 45-minute sessions | New immigrants from Ethiopia and the former Societ Union were recruited. Age group is 4–7-year-olds. Mean age was 63.4 months. 113 males 125 females. |
| (Kennedy et al., 2014) | Journal of Contextual Behavioural Science. | USA |  | To examine the effect of mindfulness as conceptualised within ACT as an alternative to modelling within a treatment package on children’s consumption of previously avoided healthy foods. | 30 weeks | Typically developing 3-5-year-olds who attend full day pre-kindergarten classroom. |
| (Kong et al., 2016) | American Journal of Preventative Medicine | USA | Chicago | To investigate the changes in BMI z score as a result of the Hip-Hop to Health Jr intervention program | 14 – weeks | Children aged 2-5 years of age, targeting African American children from low-income families. 33% of children were above the 85^th^ percentile for BMI |
| (Kornilaki et al., 2022) | Early Child Development and Care | Greece | Crete | The primary aim was to examine changes in young children’s healthy living or sustainability knowledge over time. The secondary aim was to see if any changes in eating and physical activity habits occurred. Tertiary aim was to examine changes in child body mass index (BMI) over time. | 4-6 weeks of curriculum. | Children in the control had a mean age of 5.05, and the intervention had 5.03. Similar numbers were born in Greece 94.8/94.6%. Roughly 11-13% children measured overweight and 4-9.5% of the children measured obese, this was slightly less in the intervention group. All children were Caucasian and fluent in Greek. |
| (Kristiansen et al., 2019) | BMC Public Health | Norway | Vestfold and Buskerud | To develop, implement and evaluate the effect of a multi-component intervention study conducted in the kindergarten and home setting. | 6 months – one year follow up. | 3–5-year-olds.  The study population was approximately 51% girls with 52% born in 2010. 67-70% of mothers had university/college education. |
| (Kristiansen et al., 2021) | BMC Research Notes | Norway | Helsinki, Vestfold and Buskerud. | To explore the association between kindergarten staffs’ perceived usefulness of the intervention components and changes in children’s vegetable intake and vegetables served in the kindergarten from baseline to follow up 1. | 6 months – one year follow up. | 3–5-year old’s.  The study population was approximately 51% girls with 52% born in 2010. 67-70% of mothers had university/college education. |
| (Kristiansen et al., 2020) | BMC Research Notes | Norway | Oslo | To report on the long-term effects of a cluster randomized controlled kindergarten-based intervention trial on vegetable intake among Norwegian preschool Children. | 6 months | Children were recruited from 3-5 years at baseline. At baseline the characteristics showed 50.8% girls, predominantly completed by the mother. 70.6% showed maternal education at university level. |
| (Lanigan et al., 2019) | Journal of Nutrition, Education and Behaviour | USA | Washington State | To determine whether the use of child-centred nutrition phrases (CCNP) with repeated exposure (RE), improved willingness to try, liking, and consumption of healthful foods compared with RE alone. | 6 weeks | Children aged between 3-6 years. 67% white middle income homes, parents with some higher education. |
| (Larsen et al., 2017) | Journal of School Health | USA | California | To evaluate the public health impact of Building a Healthy Me (BHM) in California Kindergarten Classrooms using the RE-AIM framework. | 6 months intervention implementation. | The intervention group had a mean age of 5.4 years old (SD – 0.5) and was 49% female. The control group was on average 5.5 years old (SD = 0.50), AND 47% female, the was also a statistically higher proportion of Hispanic or Latino students compared to the intervention group. |
| (Lee et al., 2017) | BMC Public Health | USA | Phoenix | To evaluate the efficacy of SAGE on improving Physical Activity and fruit and vegetable consumption, hunger and fullness cues among children. | 12 one-hour sessions twice a week. | Children aged between 3 and 5 years were recruited. 55% were male, children were on average 3.2 years of age (SD = 0.72). The majority of the participants were Hispanic or Latino at 65%. |
| (Leis et al., 2020) | BMC Public Health | Canada | Saskatchewan and New Brunswick | To assess the effectiveness of the HSDS intervention in increasing physical activity levels and healthy eating as well as improving fundamental movement skills In preschoolers attending ECC | 6-8 months | Children with a mean age of 4.1 years. 54% boys in control group and 51% boys in intervention group. Bilingual English and French. Between 58-65% Urban population. Median household income is approximately 54,700 Canadian dollars. before taxes. |
| (Lim et al., 2016) | Nutrition, Research, and Practice | South Korea |  | To evaluate implementation of the adapted MX project for young children in South Korea. Investigating how effective the project is for improving eating behaviours and nutrition knowledge. | 4 weeks fitness, 2 weeks nutrition | Equal numbers of males and females 16.45 were overweight  19.2% were underweight  60% picky eaters  50% take regular supplements. |
| (Lumeng et al., 2017) | Pediatrics | USA | Michigan both urban and rural | To determine the effect of an intervention to improve emotional and behavioural self-regulation in combination with an obesity-prevention program on the prevalence of obesity and obesity-related behaviours in preschoolers. | 4 academic years | Children recruited from 3 classrooms in three separate head start facilities, beginning in the autumn term of 2011 and finishing in the spring of 2015. Child mean age in each arm is 4.12 (SD = 0.53), 4.10 (SD = 0.52), 4.12 (SD = 0.52). In each branch the percentage males is 46.3, 50.9 and 48.6 respectively. |
| (Maimaran & Fishbach, 2014) | Journal of Consumer Research | USA | Illinois | To test whether pre-schoolers infer that if food is instrumental to achieve a goal, it is less tasty, and therefore they consume less of it. | 1 day | Study 1: Children aged between 4.5 and 5.5 years of age (63% female)  Study 2: children aged between 3-4 years (41% female)  Study 3. Children aged between 4-5 years (46% female) Study 4: Children aged between 3.5-4.5 years (50% female) Study 5: Children aged between 3.5-4.5 years (45% female) |
| (Maimaran & Salant, 2019) | Judgement and Decision Making | USA | Northwest, pre-school setting | To examine the effect of limited availability on the engagement, consumption, and choice behaviour of four- to five-year old children. | 1 day | 3 studies:  Study 1. M age = 61.98 months SD = 5.68, 53% female Study 2. M age = 61.27 months SD = 4.00, 51% female Study 3. M age = 56.9 months SD = 3.99, 48% female |
| (Marshall et al., 2020) | Preventive Medicine Reports. | USA |  | To present the findings of a two year follow up study to determine the program maintenance effects of a quasi-experimental non-randomised controlled study assessing the impact of Brighter Bites among 1^st^ grade children. | 16 weeks | Baseline: 55.9% of households were bilingual, 52.8% of child participants were girls, 44.0% of children were overweight or obese. Child participants were aged 5-7 years old (mean = 6.12, SD = 0.34). 75.5% of parents were Hispanic, 21.3% were African American. 19 fathers and 1 grandmother also participated in. The average household size was 5.28 (SD=5.73). |
| (Mathias et al., 2012) | Journal of the Academy of Nutrition and Dietetics | USA | Philadelphia Metropolitan area | To test whether vegetable portion size would be affected by the amount of fruit served, and to test whether portion size effects would be influenced by degree of liking a particular food. | Once weekly for 5 months. | Children aged between 4-6 years, mean age 5.6 years (SD = 0.2), with half of the children classifying as overweight or obese. Half of the primary caregivers had either part-time or full-time employment. 18 of the children were girls, 14 were non-Hispanic black/ African American and 9 were non-Hispanic white. |
| (Melnick et al., 2020) | Public Health Nutrition | USA | Denver Metro Area | To evaluate the Culture of Wellness in Preschools Nutrition Education (COWP-NE) programme. | 1 year | Children aged between 3-5 years old.  96.0% of the intervention group received a free lunch compared with 71.1% of the control.  22.9% of the intervention were white, 54.1% Hispanic/Latino, 14.4% black and 8.6% other.  36.6% of the intervention were white, 54.3% Hispanic/Latino, 3.7% black and 5.4% other. |
| (Mohd Nor et al., 2021) | Elsevier | UK | Reading and Wokingham, primary schools | To determine the effect of repeated taste exposure on acceptance of an unfamiliar Brassica vegetable among children with varying bitter taste sensitivity. | Pre-intervention test (no time given), intervention and 3 months post intervention | Children aged 3 years 1 month to 5 years 7 months, M = 4 years 9 months Children unfamiliar with turnip 82 males |
| (Morris et al., 2016) | Early Child Development and Care | Australia | Melborne Victoria | To evaluate the effects of the intervention on children’s knowledge about healthy eating, active play and the sustainability consequences of their food and toy selections. | Approximately 6 weeks from Beginning of August to Mid-September. | The intervention was delivered to six kindergarten groups. The two groups did not differ in demographic variables apart from maternal age where the intervention mothers were slightly older. Child age intervention M = 4.57 years, Control M = 4.69 Intervention 80.4% normal weight, Control 83.3% normal weight. |
| (Morris et al., 2018) | Australasian Journal of Early Childhood | Australia |  | To investigate the effect of teacher-designed play-based learning experiences on four-year-old preschool children’s knowledge connections about wellbeing and sustainability. | 8 weeks – 3 months post intervention. | Average child age is 4.76 in the intervention group and 4.68 in the control group. The children were mainly from high income families, approximately 50% of mothers and 35% of fathers had attended university. |
| (Naderer et al., 2017) | Appetite | Austria |  | To examine the influence of food placements upon children’s food choice, food-related parentally mediation strategies and BMI. | Single day | Children aged between 6-11 years of age took part in the study. Mean age was 8.41 years (SD = 1.16), 51.4% female. |
| (Natale et al., 2014) | Obesity Prevention in Children | USA | Miami | The overall aim of the study was to assess the effectiveness of a multifaceted obesity prevention intervention on BMI and dietary and physical activity patterns of inner-city multiethnic preschool children. | 6 months | 51% of the sample were boys, 36% were black, 34% white, 18% other. 35% were primarily Spanish Speaking. 16.7% of the intervention group and 14.9% of the control were overweight, 13.7% of the intervention, and 16.4% of the control were obese |
| (Natale et al., 2017) | American Journal of Health Promotion | USA | Miami-Dade County | To develop, test, and evaluate “Healthy Caregivers- Health Children” a theoretically based, multifaceted obesity prevention intervention conducted in childcare setting, targeting low-income, multiethnic children. | Two-years. | There were similar numbers of males and females recruited (Males = 50.1%). The group was 41.0% Hispanic, with a mean child age of 40.72 months (SD=11.18). 30.31% of the participants spoke Spanish only in the home, 52% had high school education with 12.8% beyond high school and 35.2% less than 12^th^ grade. |
| (Nederkoorn et al., 2018) | Appetite | The Netherlands | Maastricht | To test whether tactile exposure to a non-food texture, by it with the hands could increase acceptance of food with the same texture. | 1 day | Children aged from 3 to 10 years. Mean age 5.8years (SD = 1.8), 36 boys and 30 girls. 9 children had overweight, 4 obese and 4 children underweight. |
| (Nekitsing, Blundell-Birtill, Cockroft, & Hetherington, 2019) | Journal of the Academy of Nutrition and Dietetics | UK | Preschools in West Yorkshire England | To test the relative efficacy of repeated taste exposure, nutrition education, and a combined intervention with a no intervention control on the intake of an unfamiliar vegetable in pre-school aged children. | 10 weeks with 24 and 36 month follow up. | Children aged from 2-5 years of age.  47 children were assigned to the Taste exposure condition, 23 were male with an average age of 38.11 months (SD = 0.83).  38 to the Nutrition education, 23 were male with an average of 43.42 months (SD = 0.54)  39 to the Taste exposure and Nutrition education condition, 14 were male, mean age 40.54 months (SD = 0.65)  16 to the control, 10 were male mean age 41.75 months (SD = 0.87) |
| (Nekitsing, Blundell-Birtill, Cockroft, Fildes, et al., 2019) | Journal of the academy of nutrition and dietetics. | UK |  | To test the effect on intake of learning about an unfamiliar vegetable (celeriac) through storybooks and sensory play. | 2 weeks | Children aged 2 -5, with a mean age of 38.9 months (SD = 0.5). No differences observed for dfferences in baseline characteristics. |
| (Nicklas et al., 2017) | International Journal of Behavioural Nutrition and Physical Activity. | USA |  | To test the feasibility of an innovative approach to increase the consumption of vegetable dishes by preschool children who were predominantly low-income African Americans and Hispanics. | 5 weeks. | Children mean average of 4.4 years old, 49% boys, 66% Hispanic and 34% African American. |
| (Nyberg et al., 2015) | Plos One | Sweden |  | To evluate the effectiveess of the 6-month Healthy School Start Programme on Children’s physical activity and healthy eating habits and on the prevention of overweight and obesity in six-year-old children attending pre-school class. | 6 months | 70% of parents were Swedish, 7% born elsewhere in Europe, and 23% outside of Europe.  33% of parents in intervention group and 40% in control group had a low level of education. Control group were 55 boys and 57 girls. Intervention was 68 boys and 61 girls. |
| (O'Connell et al., 2012) | Journal of the Academy of Nutrition and Dietetics. | USA | Small north-eastern city. | To test whether children in a community preschool would increase consumption of three unfamiliar or disliked vegetables after being offered each of them 10 times during lunch over a 6-week period. | 6 weeks | 54 male children and 42 female children took part, predominantly from highly educated households. Ages ranged from 3 to 6 years old with most between 4 and 5 years. Ethnicity was 69% white, 8% Asian, 5% African American, 6% Hispanic and 12% other. |
| (Olsen et al., 2019) | Food Quality and Preference | Denmark | Copenhagen | To investigate whether offering a choice (in contrast to no choice) increases the intake of snack vegetables in pre-school children. | 4 weeks | Children aged 2-6 years (mean = 49 months SD = 11) 58% male |
| (Pathirana et al., 2018) | Health Promotion Journal of Australia | Australia |  | To assess the short-term changes in dietary, physical activity and sedentary behaviours of children following participation in the HFBH project. | 8 weeks. | Children under 5 years of age, (M = 3.84 years) from low socioeconomic backgrounds. 95.4% English speaking, |
| (Pinket et al., 2017) | Nutrients | Belgium | 6 European Countries including, Belgium, Bulgaria, Germany, Greece, Poland, and Spain. | To examine whether the ToyBox-intervention can affect total diet quality and the four subcomponents of diet quality developed by Huybrechts in European preschoolers. | For one academic year. | Mean overall age of all participants is 4.7 (SD = 0.4)  Overall, the study included 51.4% males 38.5% lower SES  These numbers varied between countries with the lowest SES at 20.7 in Poland and the highest at 51.4 in Germany. The percentagest of males varied from 49.8% in Bulgaria to 54.5% in Spain. |
| (Piziak, 2021) | Sustainability | USA | South and Central Texas | To discuss the development of enjoyable, inexpensive, sustainable games for educating families about basic nutrition and increasing exercise activity in preschool children to help decrease the development of early diabetes and cardiovascular disease. | The intervention for Bingo game lasted a school year, The video game testing during two sessions in a summer session | Children aged 3-5 were recruited into this study. The group in Texas is predominantly Hispanic, The game was designed for a bilingual setting. Children were recruited from the Head Start programme, a funding source for low income families. |
| (Ray et al., 2020) | Nutrients | Finland | Helsinki, Salo, Riihimaki municipalities. | To evaluate the effects of a preschool-based family intervention on children’s EBRB and SR skills and to evaluate whether effects were stronger among children with low PEL background compared to those with high PEL background. | 8 months | 54.4% boys in the control, 52.2% in the intervention.  43.6% parents were in the middle education level in the control group. 46.4% in the control group. |
| (Rioux et al., 2018) | Appetite | France |  | To explore further the potential for visual exposure and to investigate the mechanisms responsible for its impact. | 4-week study. | Children aged between 34-68 months, 40 girls and 30 boys, mean age 51.43 months (SD= 8.62). |
| (Roberts et al., 2022) | Appetite | UK | Vestfold and Buskerud | To compare the effects of unisensory versus multisensory exposure conditions on children’s immediate acceptance of foods. | 1 day | Children aged between 3 to 5 years. 64 males, 46 females, mean age 46.42 months (SD =5.78). |
| (Roe et al., 2013) | American Journal of Clinical Nutrition | USA | University park campus Pennsylvania State University | To explore the effect of variety by offering children in a childcare facility a variety of familiar vegetables or fruit as a snack and comparing this with offering each type individually. | 4 weeks | Children aged between 3-5 years of age, there were 32 girls and 29 boys. The children were 56% white, 29% Asian, 11% black and 4% pacific Islander. |
| (Roe et al., 2022) | The American Journal of Clinical Nutrition. | USA | University Park childcare centres, Pennsylvania, | To determine the effects on energy intake of varying only the proportion of vegetables and fruits, and to examine whether these effects differed for the strategies of substitution and addition. | 5 days. | Children aged 3-5 years. There were 28 boys and 25 girls, with an age range from 3.0 to 5.8 years mean = 4.4 (SD = 0.1). 16% of the children were classified as having overweight or obesity. 58% were white, 13% were Asian, 4% black and 13% mixed. |
| (Rohlfs Domínguez et al., 2013) | Food Quality and Preference | Spain | Granada | To evaluate the effectiveness of providing either choice between two vegetables simultaneously available in the dish, or the possibility to pre-select one of them in increasing vegetable consumption during the regular school meal setting. | Two weeks | Children aged 4 to 6 years old. |
| (Rollins et al., 2021) | Appetite | USA | Pennsylvania | To investigate if children’s food avoidant behaviours and food approach behaviours would predict their responses to the FRUIT+ Dark Green Vegetables (DGV) contained within the smoothies. | Single school day. | Children aged between 3-5 years old, in the fruit only group there were 55.6% males and 44.4% males in the fruit and DGV group. In the fruit only group 12.5% were overweight and 16.7 in the group with added DGVs. Both showed Mothers with higher education at 75% Graduate education in the fruit only and 68.6% in the added DGV. 70.9% were from a white background. |
| (Savage et al., 2013) | The academy of nutrition and dietetics | USA | Childcare centre in central Pennsylvania during 2008-2009 | To compare the effects of dips, with and without familiar herbs and spice combinations, with servingTo compare the effects of dips, (with and without familiar herbs and spice combinations), with serving vegetables alone (without dip) on children’s willingness to taste, liking of, and consumption of vegetables. | 6-weeks | Children aged 3-5  40/46 were white 98% lived in household where parents were married Households where parents were employed, mothers 96%, father 98% Median household income was between 61k-80k Majority of parents had a bachelor degree or above 53% female |
| (Serebrennikov et al., 2020) | Plos One | USA | MidWestern State | To understand the average treatment effect of a classroom-based intervention on students’ food selection and food waste. | The intervention classes were 15-20 minutes long for a period of 6 weeks. | The mean age was 94.8 months, 42% female, 91.4% white. |
| (Sharma et al., 2016) | Preventive Medicine | USA | Texas | To determine the effectiveness of Brighter Bites in improving intake of Fruits and vegetables and parental food practices, rules and mealtime environment among 1^st^ grade children and their parents. | 16 weeks | Average child age was 6.1 years. Average parental age was 34 years in total there were 51.9% females. 71% of participants were Hispanic, 23.6% were black or African American. 17.2% were overweight and 25.5% were obese. |
| (Smethers et al., 2019) | The American Journal of Clinical Nutrition | USA | Pennsylvania. | To test whether the portion size effect is sustained in preschool children across 5 consecutive days, a period thought to be sufficient for regulatory systems to respond to the overconsumption of energy. | 5 days | 30 boys and 16 girls aged mean age 4.4 years (SD= 0.6) 11% classified as having overweight or obesity, 74% white, 9% Asian, 6% mixed. |
| (Smith et al., 2013) | BMJ | UK | 37 UK wide locations | To report outcomes of the UK service level delivery of MEND (Mind, Exercize, Nutrition…Do it!) designed for overweight and obese children aged 5-7 years. | 10 weeks Pre and post intervention (approximately 3 months) | Overweight or obese children aged 5 – 7 years. Mean age = 6.1. 67.2% White ethnicity, BMI score = 2.86, 42% boys. |
| (Staiano et al., 2016) | Journal of Nutrition Education and Behavior | USA | Los Angeles | To determine the influence of screen-based peer modelling on children’s vegetable consumption and preference. |  | 50% females, mean age 4.3, Range 3-5. 73.8% White. |
| (Steenbock et al., 2019) | BMC pediatrics | Germany |  | To evaluate the multi-component health promotion program, jolichenkids – fit and healthy in daycare, designed to promote physical activity, healthy eating and mental wellbeing among 3- to 6-year old preschoolers. | Baseline and 1 year follow up. | Intervention group was 50.5% boys, control was 51.7% boys. Mean age for both groups was 4.3 years. 85% of the intervention group were underweight or normal weight, 89.5% of the controls. 72% of both groups attended the preschool for more than 5 hours a day. Over 70% of the children in both groups had no migration background. |
| (Suarez-Balcazar et al., 2014) | Journal of Prevention and Intervention in the Community | USA | Urban Midwestern City | To examine the impact of nutrition education on children’s eating habits in a target school compared to a school that did not receive the nutrition education programme. | Twice a month (total of 10 times) Sessions of 30-45 minutes | The target school was majority Hispanic, the comparison school is mostly African America, Kindergarten consists of children aged between 4 years 7 months to 6 years, and first grade is 6-7 years old. |
| (Tani et al., 2021) | Nutrients | Japan | Adachi Tokyo | To examine the associations of nursery school-level vegetable-eating promotion with vegetable consumption behaviours and BMI among Japanese children. | Cross-sectional measured in 2015, 2016, 2017 | Children aged between 4-5 years of age,  13% on average of the sample were overweight.  Approximately half of the children were girls.  60% high to middle economic status. |
| (Toossi, 2017) | Appetite | USA | New York | This study aimed to establish the effectiveness of using small reward-based incentives in inducing the choice and consumption of healthier foods among children. | One month | Children aged 5-8 were recruited. 61% male, 74% Black, 9% White, 13% Mixed (Black and White), 4% other. 91% non-Hispanic. 65% single mother households. 48% <10,000 household income. 39% had one sibling. |
| (Toussaint et al., 2021) | PLoS ONE | Netherlands | 41 preschools of deprived areas in Amsterdam | To investigate the effect of an intervention for teachers in promoting healthy eating and physical activity in young children | 9 months | Teachers: 100% female 38% Dutch, 33% Moroccan M age = 42 years  Children: 49% female 35% Moroccan Overweight 13%, obese 3% M age 3.0 |
| (Van Stokkom et al., 2018) | Appetite | The Netherlands | Sydney Australia | To investigate the effect of sweetness and sourness enhancement on acceptance of cucumber and green capsicum purees in 5–6-year-old children. | 30 minutes | Children aged 5-6 years old. The mean age of the children was 5.7 years (SD = 0.5), 54.3% were girls. Both parent and child vegetable consumption were below the recommended intake. |
| (Vandeweghe et al., 2016) | Appetite | Belgium | Ghent | To investigate the effectiveness of different strategies to improve Willingness to Taste disliked vegetables and the moderating role of Reward Sensitivity. | Single day | Children with a mean age of 4.48 year (SD=1.01), 51% males, with parents 86.2% having a Bachelor or higher. |
| (Vandeweghe et al., 2018) | Food Quality and Preference | Belgium | Deinze and Bevere | To investigate the effectiveness of different strategies in preschool children in an ecologically valid context to increase willingness to try and liking of vegetables. | 17 weeks in total. | Children aged 3-6, Mean age was 5.08 years (SD = 0.61). |
| (Vaughn et al., 2021) | Null Results Research | USA | North Carolina | To test the effectiveness of an ECE-based nutrition and physical activity social marketing intervention. | 8 month | Three- to 4-year-old children and their parents from 92 childcare centres in North Carolina. 46% of the children were white, parents were on average 33.5 years old. |
| (von Nordheim et al., 2022) | Appetite | UK | Frankfurt and Offenbach | To investigate children’s actual intake of exclusively healthy foods in response to healthy food advertisement exposure in five nurseries in Germany. | Single day Control group pre-test – post-test experiment. | Children aged 3-7 years were included (mean = 4.72, SD 0.99). There were 50 females in the intervention group and 39 in the control. |
| (Whiteside-Mansell et al., 2021) | Journal of Hunger and Environmental Nutrition | USA | Southern Rural state | To evaluate fruit and vegetable consumption of young children experiencing the Together We Inspire Smart Eating (WISE) program. | 1 -day | Participants were Either English or Spanish Speakers. Most of the participants were reported as white, 42%, 33% were reported as Black. 12 Head Start Classrooms and 12 Public elementary school classrooms with equal numbers of kindergarten classes and first grade classes were recruited. |
| (Williams et al., 2014) | Journal of the Academy of Nutrition and Dietetics. | USA | State of New York | To test the impact of the program | 6- 10 week period | The average age of the child was 4.45 years, 52% of the population was male. Children in the intervention group were statistically older than children in the control group by 0.2 years. Parents were aged between 18 and 24, 40% were Hispanic of Latino, 24% were white non-Hispanic, 27% were black non-Hispanic. |
| (Willis et al., 2014) | Pediatric Obesity | UK | 9 locations across England | To investigate the impact of the intervention by following a cohort of parents completing the course at several locations in England, and comparing data from before and after the course, and at 8 week follow-up to assess if changes are sustained. | 8 weeks | Age of parents mean 30.37, range 18-40.  96.7% females, 52% White British,  83.3% completed further education.  Average number of children living at home = 1.67 Age of children at home mean = 3.32- SD = 4.04. |
| (Witt & Dunn, 2012) | Journal of Nutrition Education and Behaviour. | USA | Boise School District. | To determine whether Color Me Healthy (CMH), an interactive nutrition and physical activity program for preschool children increases fruit and vegetable consumption. | 6 weeks | Preschool children in 17 childcare centres. 28% of the children attend for mornings, 28% for all day and 44% for the afternoon. |
| (Yoong et al., 2020) | The American Journal of Clinical Nutrition | Australia | New South Wales | To examine the impact of an implementation intervention consisting primarily of web-based menu-planning tool, with support targeting cooks to improve the provision of foods in childcare centers in accordance with dietary guidelines. | 12 month implementation intervention | Children aged 3-6 years were measured, The average age of children at baseline was 4.6 years. 49.6% female. Average attendance at the childcare centre was 3.25 days per week. 73 % of the children attend for more than 3 days per week. |
| (Zeinstra et al., 2017) | Food Quality and Preference. | The Netherlands | Arnhem | To investigate the effect of TV idol role modelling in combination with repeated exposure on children’s intake of a familiar vegetable, and to study whether a period of restriction would enhance this effect. | 9 months | Children were recruited through a primary school in the city or Arnhem, Children were an average of 4.8 years old, they were considered ‘normal weight’ and scored in the middle of the neophobia scale. The liking for raw carrots was slightly higher than neutral with a mean score of 3.3 on a 5 point scale. 55% of the mothers were highly educated, 35% were middle and 10% low. 51% were girls. |
