# Supplementary material for: Improving Children’s Diets by Introducing Fruits and Vegetables in Group-Based Settings: A Scoping Review
Source: Nutr Rev. 2025 Jul 3;84(5):1039–50. doi: 10.1093/nutrit/nuaf092 (PMC13075485; doi:10.1093/nutrit/nuaf092)
Supplement: nuaf092_Supplementary_Data [file nuaf092_supplementary_data.zip › Supplementary Table V. Description of Intervention.docx]

Supplementary Table V. Description of Intervention methodology.

| **Study** | **Intervention type and duration, comparison, outcome measures** |
| --- | --- |
| (Adab et al., 2018) | - The WAVES study targeted both the home and school environment in year 2 children (aged 6 or 7) and also involved their families. - Behavioural change strategies were employed to increase physical activity and improve diet quality. - School staff were provided with training and resources for intervention delivery. - The intervention involved 30 minutes of vigorous physical activity on each school day, - 15 minutes of outdoor break time. - Each term there was a cooking workshop during the school day with parents invited to participate with their child. Materials for the workshop as well as take home information was provided. - A six-week program called Villa Vitality was developed to encourage healthy eating and increase physical activity and delivered by staff from an iconic sporting institution. - Information sheets signposting children and their families on ways to be active over the summer (identical for all schools) and physical activity opportunities in their local area (school specific sheets produced by the study team and checked before distribution by the school). |
| Ahern et al., 2019) | - An intervention was developed with three target vegetables. - Vegetables that were deemed familiar to this age group of children were used as identified in a previous study. - The vegetables were baby sweet corn, celery and red pepper. - Two conditions were used   - Single snack (RE condition)   - Mixed Vegetables (V condition) - The mixed vegetables also included radish and green pepper that were deemed familiar but not typically consumed as snacks. - All children were offered a bag containing a single target vegetable and the mixed vegetables on two separate days. This was counterbalanced. - During the exposure phase children received 5 exposures to either the single snack or the mixed vegetables. - Intake was measured after each exposure - Post intervention intake of both the single vegetable and the mixed vegetables was measured 2-5 days after intervention. - Single snack contained 100g of one of the target vegetables. The mixed snack contained 20g of each of the 5 vegetables included. |
| (Anzman-Frasca et al., 2012) | - Food liking for the study vegetables was established before and after the intervention. - The intervention followed a Repeated exposures method to introduce a vegetable to the children. - Pre- and post-test liking, and intake assessments took place, as well as liking assessments during the intermediary tasting trials. - During all liking assessments, children visited the tasting station in the classroom one at a time to taste a small portion of vegetable(s), with dip if applicable. - During intake assessments, children were seated at a table with others. - In the pre and post-test liking assessments, five vegetables were tasted. - The assigned vegetables were tasted and rated without dip in both conditions. - During the tasting trials and intake assessments, only the assigned vegetables were served. - they were served with a liked dip in the AC condition. - The tasting session involved children being asked twice weekly over a period of 4 weeks to take of taste of a very small portion (4 g) of the vegetable in its assigned condition. - Children rated the vegetable in its assigned condition using the same three categories as in the pre-test: yummy, just okay, or yucky. - There was 1 week of make-up sessions to allow children who had missed an exposure session to complete missed sessions - There was also an additional, follow-up tasting trial that took place one month later to determine whether effects were sustained |
| (Bai et al., 2018) | - The intervention consisted of a weekly 40-minute session over the period of 4 weeks. - The sessions consisted of a classroom lecture and hands-on cooking activities. - Two vegetables were introduced in each session. - These vegetables included Bell pepper, Daikon, Zucchini, Tomato, Sweet potato, Spinach, Cucumber, Oyster mushrooms. - For the first 10 minutes a nutrition teacher would introduce the two vegetables, and their health benefits, children were encouraged to try the vegetables that they had prepared. - Eight new recipes were developed to include each of the vegetables, as well as vegetables that they were familiar with. - The title song of the original Veggication was played in the background and poster materials were displayed in the classroom. |
| (Belot et al., 2016) | - The two treatments we designed incentivize choice (rather than consumption) of fruit or vegetables at lunch. - In both schemes, children received a sticker each day if they chose or brought in a fruit or vegetable at lunchtime. - Then, at the end of the week (Friday afternoon after lunch), each pupil had the opportunity to pick a larger prize depending on the incentive scheme in which the pupil was enrolled. - In the individual incentive scheme, if a pupil collected four stickers in the week, she or he was allowed to choose a prize such as an item of stationery or a small toy from a reward box. - If the pupil had three or less stickers, though, the pupil could not pick a prize and the stickers did not count to earning an award the following week. - In the competition, children were assigned to random groups of four, and only the pupil with the most stickers in each group was able to select a prize from the reward box. - In the case of a tie, all children with the highest number of stickers in the group were eligible for a prize. - The groups were revealed at the end of the week after lunch so children would not engage in strategic behaviour, such as making choices based on other group member’s actions or absenteeism. - The groups were changed each week so the children could not anticipate with whom they would be competing and, in this treatment as well, unused stickers did not carry over to the following week. |
| (Boyer et al., 2012) | - On the study days, the regularly scheduled snack foods at the childcare centre were replaced with one of three different high-fibre, low-fat snack foods. - Food choices included banana bread, pancakes, and wrap-type turkey and cheese sandwiches. - These foods were developed by the researchers to provide high fibre density (3 to 6 grams per serving) and to be low in total fat. - To test the snacks for acceptability, all 3 snacks were field tested in another daycare prior to the onset of this study. - Each snack was served to ap- proximately 20 children, who rated the food using an age-appropriate three-level Likert scale as either “dislike very much,” “neither like nor dislike,” and “like very much.” - Recipes were modified until at least 50% of the children liked the food and at least 80% of the children liked the food or had a neutral response. - The snacks were scheduled in a three-week menu rotation in which one of the three snacks was served twice every week and no snack was repeated two days in a row. - The snacks energy ranging between 150 and 200 calories per serving of each snack.. - The questionnaire included questions about the time, type, and amount of food consumed. Previous intake was coded as yes (=1) or no (=0). |
| (Braga-Pontes et al., 2022) | - The intervention consisted of 20-min educational sessions once a week for 5 weeks. - Educational sessions were realised in preschools allocated with one of the nutrition education strategies verified in this study (Digital Game, DG, Story-Book, SB or Storybook and stickers SBS). - All the interventions were conducted simultaneously. - The intervention was carried out by three groups of researchers and the main researcher was present in all groups. - The DG consisted of five mini-games and each of these had a vegetable superhero (tomato, purple cabbage, cucumber, carrot and lettuce) associated with it. - Besides, the DG included tailored audio messages about the characteristics and health benefits of these vegetables. - The SB was made up of five chapters and each one had a vegetable superhero, equal to the DG, and it was clear in the story the characteristics and functions of the vegetables. - In the group of SBS, the educational sessions were based on the SB and children received a reward (sticker)when they ate the vegetables at the end of the session. - The control group realised educational sessions with the PFWG, the gold standard tool in nutrition education in Portugal, and it was used to promote the group of vegetables. - At the end of each session, a play food was distributed to each child and they had to place it in the right group of the PFWG. - Each week a real vegetable was distributed (tomato, purple cabbage, cucumber, carrot or lettuce), according to the vegetable that was in the DG or the SB, allowing each child to explore sensorially the vegetable. - At the end of each educational session, in all groups, the five vegetables cut in similar portions were offered one after the other to each child. - The order to offer the five vegetables was different during the 5 weeks of intervention. - Children could serve themselves from a shared plate twice and eat the number of portions they wanted each time. - The procedure was consistent across the preschools. - Classroom staff were required not to motivate or congratulate children for eating vegetables. |
| (Brennan et al., 2021) | - ‘Nourish’ was informed by pre-intervention observations conducted at schools (n = 11) in the region which captured information on current practices with the school food environment such as canteen protocols and systems, proportion of children who consumed school dinners versus packed lunches, school food provision and equipment and food- related events. - These observations were conducted with input from school senior management and catering personnel. - Specifically, the ‘Nourish’ intervention included provision of healthy snacks e.g. fruit, a rotation of breads (including wheaten bread and high fibre bread with accompanying butter for spreading) and milk. - Resources to improve school food presentation, cookery equipment and recipes which included all food groups; sensory education material; catering for school events; and attendance at Tasting Days. - The ‘Nourish’ intervention also involved holding discussions with relevant school staff to help support the implementation of school food policies. - Schools received a document highlighting relevant Public Health Agency Northern Ireland guidance that would support them to implement their school food policies. - The ‘Engage’ intervention was an age-appropriate, cross-curricular educational intervention on food, agriculture, food and nutrition-related science and related careers. - ‘Engage’ included topics such as the food chain, product development, growing food, animal welfare, sustainability, food labels, portion size and diet and health. - The ‘Engage’ intervention incorporated aspects of the current Northern Ireland Curriculum. - The ‘Engage’ intervention aimed to increase children’s knowledge about all food groups, understanding of how to prepare food and cooking methods. - Importance was placed on healthy diet and dietary intake. |
| (Bucher Della Torre et al., 2015) | - Croque & Bouge, which translates as snack and move is a workshop-based programme run by dieticians. - Parents work together with their children. - The parents and children take part in a cooking task as well as a tasting task at the end of each session. - This intervention is based on the Health Belief model. - The dietician works with parents to discuss perceived barriers to action, aimed to increase self-efficacy improve understanding of the benefit of acting. - Parents also performed blind tasting and sensory description exercised. - Children played games with fruit and vegetable. These were card games specifically designed for this programme, based on dominos, happy families games and such. They also listened to stories about feelings of hunger and satiety and completed tasks to improve fruit and vegetable recognition. |
| (Capaldi-Phillips & Wadhera, 2014) | - Fourteen conditioning trials were conducted at approximately 9 AM in each classroom. - Children received the vegetable snack 5 days per week from Monday to Friday. - One trial per day for each vegetable - Total of seven trials per vegetable. - Children were seated in groups of five or six with a researcher or teacher. - Each trial consisted of serving the vegetable with or without the cream cheese. - Children were encouraged to smear the vegetable with cream cheese using the ice-cream stick provided. - Researchers, teachers, and student workers ate with the children and were instructed not to show any negative or positive facial expressions that could influence children’s intake. - Teachers were instructed to refer to cauliflower as “power flowers” and brussels sprouts as “power sprouts”. - After the snack, any left-over vegetable and cream cheese were weighed using and recorded using a digital scale . - Thirty minutes after the vegetables were consumed, children were provided with crackers. - Testing. On the 15th day, all children were tested for their liking of both vegetables. - Both vegetables were served on a tray without cream cheese. - Children were then asked to taste the vegetables, and they indicated their liking for the vegetables using three pictures of cartoon faces varying in expression. - Each child was individually asked to point to the vegetable and describe whether they liked it by pointing to the happy face, or disliked it by pointing to the sad face, or if it was “okay” by pointing to the neutral face. - Mean consumption of the vegetables was also measured during testing because it is a better indicator of liking in children. |
| (Carney et al., 2018) | - On each of the two visits, children consumed an ad libitum multi item test meal consisting of macaroni and cheese (175g), unsweetened applesauce (115g), 2% milk (240g) , water (465g), and three servings of microwave steamed crinkle cut carrots (40g each). - Small (less than 5g) samples of each of the foods were given and rated for liking before the meal. - Between the liking and the foods from super bad to super good on a Visual Analog Scale. - Children also rated how full they felt prior to the test meal. - In the variety session each serving of carrots was seasoned with either cinnamon-nutmeg-ginger, cardamom-cumin-allspice, or garlic-black pepper-oregano mixed seasonings. - Children were given 30 minutes and told they could eat as much as they wanted whilst a research assistant read a non-food related story as a neutral distraction. - The liking assessment was then completed again as was the perceived fullness. |
| (Carstairs et al., 2018) | - Investigation of the effect of downsizing. - High energy density cheese sandwich - Low energy density vegetable (cucumber, cherry tomato, carrots) - Fruit and yoghurt - 4 weeks with 100% portion size exceeding age specific recommendations - 4 weeks following that showed 40% downsizing to give 60% Portions that matched portion size recommendations for this age group. - Children were presented with the test meal at lunchtimes, advised that “they could eat as much or as little as they liked” - Liking was measured by asking the children whether they thought the food was “yummy” “yucky” or “just ok” |
| (Chen et al., 2014) | Recipe development   - Seven cultural/ethnic food recipes representing Hmong, Latino, and mainstream American cultures were developed. - Each recipe featured a local ethnic produce item along with a graphic tutorial. - Recipe cards also featured “fun facts,” food history, food culture, and nutrition content information. - Food provided in the take home kits were a combination of affordable, locally available fresh vegetables/legumes and cans (e.g. Black beans, chickpeas). - Those recipes involved simple food preparation methods and minimal heating.   Classroom component   - From February to May 2012, one to two cultural/ethnic food recipes were used to conduct in-class monthly tasting activities. - Nutrition educators demonstrated the recipes and taught information provided on the recipe cards. - Teachers facilitated food demonstrations and tasting activities. - It took approximately 20 minutes to complete in-class activities for one recipe.   Family component   - Prior to the food demonstrations and tasting activities, food kits were prepared containing the cultural/ethnic food recipe cards, the necessary ingredients, and a parent feedback form. - Students received the kits after the in-class activities and were encouraged to make the same recipe at home with help from their parents. Back- packs containing cooking equipment, such as measuring cups, aprons, cutting boards, and spices, were distributed to the students before the start of the intervention. - Magnets with pictures of the featured vegetables and recipe books were given to the students if the recipe and a parent feedback survey were completed at home. |
| (Choi et al., 2018) | - Weekly nutrition education was provided to the enrolled preschoolers once a week for 8 weeks - The intervention was based on learning the colours of the rainbow as represented by the colours of fruits and vegetables. - In the first week, the preschoolers were taught about red fruits and vegetables, as well as dietary guidelines, and were prompted to make a healthy sandwich with vegetables. - The second week’s education was about orange fruits and vegetables and guideline along with participation in play activity related to how to prepare breakfast. - In the third week’s intervention, preschoolers were taught the yellow fruits and vegetables as well as dietary guideline and a simple gymnastic activity. - The fourth week’s education was about green fruits and vegetables and guideline. Since guideline 6 was not appropriate for preschoolers, they were taught about how to “eat healthy snacks” by participating in a yogurt-tower cooking class. - The fifth week’s education was about purple fruits and vegetables and a guideline, and a related theme including a play quiz and play activity of how to use chopsticks. - Two of the dietary guidelines were provided via home correspondence for parents. - In the sixth week’s education intervention, preschoolers were taught about black fruits and vegetables as well as dietary guideline and they participated in a play activity related to finding healthy food. - The seventh week’s education was about white fruits and vegetables as well as dietary guideline and the intervention was accompanied by a fairy tale. - The eight week’s education was about all of the colours of foods and guideline (“Eat a variety of foods such as grains, vegetables, fruits, dairy products, meat, fish, eggs, and soybeans”). |
| (Correia et al., 2014) | - For both meals and conditions, researchers weighed and plated the children’s meals in the centre’s cafeteria in accordance with the CACFP-recommended preschool serving sizes for all meal components before delivering them to the classrooms. - All weights were recorded to the nearest 0.1 g on a digital electronic - For the lunch-pairing intervention, children were offered ½ cup of milk, ½ cup of fresh diced pears and apples, 1⁄4 small (80–90 g) pizza with marinara sauce and cheese, and 1⁄4 cup(19.1–20.1 g) steamed broccoli either on the side (control)or on top of the pizza (intervention). - Broccoli was chosen for the intervention because it was not typically served in this context in the centre but was not a completely unfamiliar food. - For the snack visual appeal intervention, children were offered ½ cup of milk and ½ cup (49.7–52.7 g) raw cucumbers either as semicircular half-slices with chive and an olive on the side (control) or arranged as a caterpillar with chive antennae and an olive eye (intervention). - Cucumbers were chosen for the snack intervention because they lend themselves to attractive appearance modifications and can be served raw, with limited preparation time, so they may be considered a reasonable addition to snack menus. - Children were free to request additional servings of any meal component. - Teachers were instructed to interact as usual with the children during meals, and helping them with additional servings. - Teachers did not engage in conversation about the intervention. - Researchers remained in the classroom to make observations during mealtime and to weigh additional servings if requested by the children. - After the meal was completed, researchers weighed the plate waste of meal components in the cafeteria. - Researchers recorded the weight and height of participating children using a digital scale and a stadiometer. |
| (Coulthard & Ahmed, 2017) | - The interventions took place in the children’s schools, either after lunch or in afterschool clubs, after snack time, to control for variations in hunger. - Groups of six children were randomly allocated to take part in a condition. - Each group was tested separately, one group at a time. - They entered the room and were asked to sit around a table together. - They were first asked to complete the picture preference measure with support from the researcher. - Next the group of six participants at a table all took part in the same intervention at one time. - A bingo game was used for the game factor based on FV. - The game had six bingo boards with four squares printed in colour and laminate. - Each board had a different combination of FVs. - The same game card was used for conditions with photographic and real FV stimuli. - The bingo game proceeded with instructions for the game. - Two small white plastic bowls were used for each child taking part in a sorting task to sort real FV or photographs of FV into each bowl. - The next stage was the free access stage where children from every condition were served a portion of fruit (20 g) and a portion of vegetables (20 g) to which they were exposed in the conditions. - Instructions given were ‘You are now going to get a fruit to try; you can eat as much as you want, and leave the rest in the bag on the table’. - The remaining FV were collected to weigh at the end of the study and measure the amount of FV eaten. - Participants were then asked to complete a picture questionnaire again. - At the end of the study each child received a FV themed sticker and certificate to congratulate them on exploring FV. - Each group of children were not limited in time however each condition lasted approximately 15 minutes. |
| (Coulthard & Sealy, 2017) | - The duration of the task and researcher carrying out the task was consistent across all three conditions. - The content of the tasks differed slightly between the three conditions. - In two of the tasks (FV play and non-food play) which involved children creating a sensory picture, the format of the task was identical. - Children were asked to sit in a group of 5 children with the researcher at an activity table. - In order to give the children some ideas for their creation there were printed images from the Very Hungry Caterpillar (Carle, 1969). - The researcher named the materials (FV or non-food), and then talked about making some pictures on the plates provided. - Children were encouraged to feel comfortable and engage in conversation during the task if they wished to. - Each child was given a paper plate on which to create their picture. - Each child created their pictures for alongside the researcher who modelled a picture of the Very Hungry Caterpillar on their own plate. - They had no implements to pick up the substances, so in order to create a picture they had to pick up the items with their fingers. - Children were encouraged to squash or reshape the foods if they wanted to. - They were not encouraged to taste the foods in the FV sensory play task, as the primary object of the task was non taste exposure. - They were given the opportunity to wash their hands after the experimental task. - In the Visual Exposure to FV condition, the groups of children watched the researcher take a picture of the Very Hungry Cater-pillar with the fruits and vegetables on a paper plate. - The format of the task was similar except the researcher modelled the creation of the picture, and the children were not given an opportunity to complete the task themselves. |
| (Crespo et al., 2012) | - This study comprised of components in either a Community intervention or a Family intervention. - For the family intervention, elements included Promotora home visits, Newsletters, recipe cards, and goal setting with the aim of educating the whole family. - There were also booster phone calls and monitoring of progress. - This focused on overcoming barriers to healthy living and ways to increase physical activity. - In the community intervention this took part within 6 schools, for a total of 3 years, and consisted of school playground improvements and salad bars to encourage healthy eating and physical activity. - Teacher’s discipline and classroom practices, encouraging water bottles in class, to use the Take 10 method and to also encourage Home fun whilst learning. - Physical education equipment improvements, and children’s menus that were created by 112 restaurants to create and modify a healthy menu. |
| (De Bock et al., 2012) | - The intervention was delivered by external nutrition experts who participated in intensive 4 day training sessions covering specific nutritional guidelines for children and basic communication skills. - The experts delivered the intervention during fifteen standardized 2 h nutrition sessions, mostly during pre-school hours. - These were conducted once weekly over a 6-month period. - Five of these sessions actively involving parents by targeting them alone or together with their children. - Intervention activities consisted of familiarizing with different food types and preparation methods as well as cooking and eating meals together in groups of children, teachers and parents. - One session additionally focused on healthy drinking behaviours. - Pre-school group teachers assisted the external nutrition expert during each session to enable them to sustain intervention-related activities after the study end. - Models for healthy eating within the intervention included:   - use of nutrition experts;   - play acting with ‘pirate dolls’ used as props enjoying fruit and vegetables   - active parental involvement; and (iv) involvement of other pre-school peers. - The exposure effect was taken into account by repeatedly offering healthy snacks like fruit and vegetables and water to the children every week. |
| (De Coen et al., 2012) | - The intervention was based on the socio-ecological model in health promotion programmes with the child as the centre of focus situated within several layers (family, friends, pre-primary or primary schools, community stake-holders, local policy and media). - The intervention was implemented over two school years (2008–2009 and 2009–2010) on different levels. - The community intervention consisted of two meetings held at the start in all communities. - Posters and information brochures were distributed in the community, through general practitioners, pharmacists, social services, and relevant community events. - The schools Focusing on in classroom child education newly developed and existing materials. |
| (de Droog et al., 2014) | - Children were read the picture book in a quiet room near their class on 5 consecutive days. - Five exposures are needed for entertainment education to take effect. - The composition of each group remained identical on each of the days. - Children were invited to eat a snack in a more comfortable eating area. - Children were permitted to eat from 4 bowls for a maximum of 5 minutes. - There were different snacks in each of the bowls and the snack size was kept consistent. - After the 5 minutes the experimenter counted how many pieces of each snack had been eaten. - The carrot was chosen, and the cartoon of a rabbit was chosen to be the food congruent character, and the tortoise was picked to be the food incongruent character. |
| (DeJesus & Venkatesh, 2020) | - Before the start of the study, a researcher prepared two foods (identical to the foods children would see in the videos). - Both foods were apple-broccoli puree. - This food was selected to be unfamiliar (to provide an opportunity to measure children's learning) and non-perishable. - The researcher first placed a 200 g calibration weight on the scale and recorded the weight. - Then, they placed a disposable bowl on the scale and squeeze puree from the pouch until the scale registered 58 g (± 1 g). - Then, they added three drops of red food colouring to one of the bowls and stirred it to blend the colour with a plastic spoon. - Food colouring was added so that the same food could be used (to control for the actual flavour of the food) while allowing children to track which food was presented. - Finally, the researcher weighed each bowl with the plastic spoon and recorded the measurement - The experimenter brought children into a small, quiet testing room and introduced the child to two actors on an iPad. - The experimenter then talked through the task describing the actors and the food that they had in front of them. - Each child was asked about their experience of that particular food and whether or not they had eaten it previously. If they had eaten it they were asked how often. - Following this the children were shown two videos. - In one video, the actor took five bites of the food and smiled (but did not speak). - In the other video, the actor said, “Oo there is some food in this bowl. I like to eat this food. I have eaten this food before, and I think this is a good food to eat” (but did not eat). - The children were then offered food whilst the experimenter did another task. - The children were allowed to eat until they rang a bell, or said they were done or didn’t want to eat any more. - The maximum length of eating was 5 minutes. - The children were then asked to rate the food on a 5-point Likert scale using “Is it yummy, yucky, or in the middle?” and if it was yummy or yucky a follow up of is it “really yummy” or “really yucky” creating the 5 point scale. - Children were then asked to compare which foods were yummier. |
| (DeJesus et al., 2019) | - For study 1, an experimenter brought the child into the testing room and introduced the child to a “teacher.” - Children sat facing the teacher at a rectangular table containing one set of bowls and foods. - The experimenter then left the room. - The teacher introduced herself to the child by saying “I’m a teacher at a school right near here. I know a lot about the foods at my school. I’m going to tell you about some foods today.” - The teacher then described each food to the child. - For the healthy food, the teacher said “This food is very healthy. It has a lot of healthy ingredients. It will make your bones and muscles get strong.” - For the unhealthy food, the teacher said “This food is not very healthy. It does not have healthy ingredients. It won’t make your bones and muscles get strong.” - After the teacher described each food, the first experimenter returned to the testing room and said that the teacher was needed elsewhere. - The teacher told the child “You can eat whatever you want,” pushed the foods toward the child, and left the room. - The experimenter remained in the room and appeared to read a magazine in the corner of the testing room while timing for 60 s, during which children could freely eat or not eat the provided foods. - This parameter was set so there would be a clear end to each trial and so that all children would have an equal amount of time with the foods (whether they ate or not). - A research assistant sat behind a screen in the testing room and triggered a light placed outside the testing room to alert the experimenter to enter the room after the teacher completed the messages so that the experimenter would be unaware of the message content paired with each food. - The research assistant triggered the light again to alert the teacher to enter the room for the second trial so that the teacher would be unaware of children’s eating behaviour in the first trial. - For study 2 the procedure was the same as study 1 except the messages provided changed. For the healthy/unpopular food, children heard “This food is very healthy but it is not very popular. It has a lot of healthy ingredients. It will make your bones and muscles get strong. But kids don’t think this food is a cool food to eat. No one eats it at school with their friends.” - Study 3 Procedure. One food was described as unhealthy and one food was described neutrally. For the unhealthy food, the teacher again said “This food is not very healthy. It does not have healthy ingredients. It won’t make your bones and muscles get strong.” For the neutral food, the teacher said “This food is right here. It has a lot of ingredients. You can buy this food at the store.” Other than the change in message content, the method was identical to Study 1. - Study 4 – was the same as study 3 except one food was described as healthy and the other as neutral. - Study 5 - Foods were described as either unhealthy or unpopular. For the unhealthy food, the teacher said “This food is not very healthy. It does not have healthy ingredients. It won’t make your bones and muscles get strong.” For the unpopular food, the teacher said “This food is not very popular. Kids don’t think this food is a cool food to eat. No one eats it at school with their friends.” The procedure and design were otherwise identical to Study 1. |
| (Dial et al., 2020) | - Assignment of the groups was conducted through order of receipt of the consent for both the intervention and the control. - Pre-intervention assessments were carried out individually. - Intervention activities consisted of 5 weeks with 10 activities. - Mindfulness focused on five senses and exploring foods both novel and familiar e.g., raisins, banana chips, bamboo shoots, dried figs. - The control group received exposure only – novel food plus fun activity. - The exposure food = radish. - Children told “ this is a radish; you can try it if you want but you don’t have to if you don’t want to”. - Fun activity – Freeze dance of Simon says. |
| (Diktas et al., 2021) | - Children enrolled in the study were served the experimental lunch in the childcare centres at the regularly scheduled time. - Children ate at tables with the same group of four to eight children and one teacher, which is standard practice at the childcare centres. - Just before the children were seated, individual portions of foods and milk were weighed and set at each child’s place at the table. - The foods were served on a 10-inch-diameter divided plate that provided separate compartments for each vegetable, and the milk was served in a 300-ml transparent cup with a lid and straw. - The children were instructed that they could eat as much or as little as they wanted but could not request more of any food or milk. - The teachers at the tables were instructed to redirect any conversation about food-related topics and to prevent children from sharing their foods and beverages with each other. - After all children had finished eating, researchers returned any dropped or spilled food to the correct plate prior to weighing. - Food and beverage items were weighed before and after meals in order to determine the amount of each item consumed by each child to the nearest 0.1 g. |
| (DiSantis et al., 2013) | - Lunch Procedures Children served themselves in a buffet-type line outside their classrooms during their normal scheduled lunch time. - This represented a departure from the typical school lunch procedures in which children typically select reportioned servings, reflecting NSLP guidelines for reimbursement. - To minimize any effects of the novelty of the self-serving procedure, an introductory session was performed to familiarize children with the procedure. - The buffet table held 3 serving bowls containing the entrée, vegetable, and fruit; children served the food in this order in each condition. - Serving bowls were filled for each child to the following weights: 825 g of penne pasta with meat sauce or ∼324 g of chicken nuggets (18 pieces), 300 g of mixed vegetables with butter, and 300 g of applesauce. Available serving portions were approximately 3 to 5 times larger than those normally offered so as to minimize potential “ceiling” effects on children self-served portion size. - To measure self-served portion size, each plate and bowl was discretely placed on a digital platform scale while children served themselves. - A remote readout was used to minimize children’s awareness that self-served portion sizes were being weighed. - Each plate/bowl was removed from the platform scale after a stabilized weight to the nearest 0.1 g had been recorded. - Children were told that they could make 1 trip through the buffet line, that they could serve themselves and eat as much or as little as they wanted, and they were not allowed to share food with other children. - Children ate at their desks in their classrooms during a 15-minute timed meal. - Research assistants were present to ensure that foods were not shared and to note any spilled or dropped foods. - Following the meal, post weights were recorded for each food using the same digital platform scales. - Self-served portion sizes were calculated as the difference in grams between pre- and post-lunch weights. - Energy intake was calculated using nutritional information provided by the school’s food service vendor. |
|  |  |
| (Edwards et al., 2022) | - Parents completed an online questionnaire about their own and their child’s characteristics. - Parents were then contacted via email to arrange an online video session. - For the session, parents were asked to prepare a bowl of raw broccoli (roughly 30 g, 5 florets) and to record the weight. - Sessions took place between 10am and 7pm, on any day of the week suitable for participants, using the online platform Zoom. - Screen share was used to show children the study materials. - First, parents reported the time since their child had last eaten. - Children gave verbal consent and rated their hunger using the Teddy Picture Rating Scale (from 1 ‘very hungry’ to 5 ‘not hungry at all/very full’; Bennett & Blissett, 2014). - Children then watched the randomly assigned video (positive, neutral or control). - After they were asked to report how they thought the models felt about eating broccoli or putting pens away, using a 3-point smiley face scale (positive, neutral, or negative). - Children were then informed that they would be given a snack to try if they would like to and that the researcher would turn off their camera and microphone whilst they were given the snack. - When ready to move on from the snack, children were told to put their thumb up, and then the researcher would return. - Parents then gave their child the raw broccoli snack, which was consumed ad libitum. - Parents were told not to pressure or encourage their child to eat the snack. - Children’s interaction with the broccoli was video recorded through Zoom. - Parents reweighed the broccoli and told the researcher the pre- and post-broccoli weights |
| (Elrakaiby et al., 2022) | - ‘Read for Nutrition’ is based on the ‘Theory of Mere Exposure’, where the development of the target vegetable’s preference and consequent consumption occurs when the child is exposed repeatedly to a positive stimulus. - The teachers receive a lesson and coaching as well as multiple exposures to book reading activity for the children. - The lesson is composed of seven topics including introduction and advantages of using evidence-based practices for the children. - After two weeks of training the teachers were asked to read the same book ‘Monsters Don’t eat Broccoli’ multiple times, at least 3 times per child per week, which is a total of 9 times over the three-week period. - Following the 3 week period the preferences and consumption of Broccoli was measured. - The Broccoli was cut into small bitesize pieces and served raw without any seasoning. - Two plates of 0.5 cup Broccoli and 0.5 cup of cereal were placed in front of the child. - The child was instructed to eat as much or as little as they would like for 10 minutes. - The amount consumed was determined by weighing what was left on the plate. - Proportional weight was calculated as the amount of food eaten/total amount of that food presented. |
| (Farrow et al., 2019) | - First, all children provided data via a short, child-friendly questionnaire which the researcher completed with them individually in a quiet area near to, or in, their usual classroom or play area. - Next, children had the opportunity to view and taste vegetables. - Four vegetables were used in this study: sweetcorn, yellow pepper, carrot and tomato. - In the Vegetable Maths Masters game children were later exposed to 2 of these vegetables (sweetcorn and carrot) and they were not exposed to two (yellow pepper and tomato). - These foods were chosen because they are similar in colour and because they can all be eaten raw. - All children were shown picture card images of the 4 vegetables and asked “Do you know what this food is called?” and “Would you like to eat some of the food?“. - If children tasted the foods they were then asked to indicate whether they liked them or not. - All foods were presented in pre-cut standardised bite sized pieces in small bowls and the researcher recorded how many pieces children had eaten. - Children were offered eight pieces of each food with the following approximate weights per bowl: 3.2 g sweetcorn; 76 g carrot; 120 g yellow pepper; and, 44 g cherry tomato. - Where children tasted the food they were asked to indicate, using an age-appropriate smiley face rating scale, whether the food was yucky (1), just ok (2), or yummy (3). - Children then played their game individually on a tablet in a quiet area near to, or in, their usual classroom or play area for 10 min. - Afterwards the procedure was repeated and children were again shown picture cards of the vegetables and asked if they could name them, whether they wanted to taste them, and if so, whether they liked them. - Children had as long as they wanted to taste and consume the foods and the researcher stayed with children throughout the procedure. |
| (Fisher et al., 2012) | - Children’s familiarity with and liking of six common raw vegetables, including two from the Brassica family, were used to identify a moderately-liked raw target vegetable and establish baseline liking. - Children were then randomly assigned by classroom to different exposure conditions that differed in if and how dressing was provided with the target vegetable during 13 exposures across a 7-week period. - Because children were assigned to conditions by classroom and not all parents provided consent for their child’s participation, the number of children assigned to each condition varied from 36 to 39. - In the two experimental dip conditions, raw broccoli was served with 2.5 oz of a regular (regular; n39) or a reduced-energy/fat(light; n36) ranch-flavoured salad dressing. - Two other conditions served as controls: one condition in which broccoli was served without dressing (plain; n39) was used to control for the effects of exposure alone - Another condition in which 2.5 oz of the regular dressing was mixed together with broccoli as a sauce (sauce; n38) was used to control for potential effects due to the act of dipping. - Children’s liking of the six common raw vegetables, including the target vegetable, was assessed following the exposure trials. - Children’s intake of a vegetable medley in the dip condition to which they were assigned was assessed following the exposure trials to determine whether effects of dip generalized beyond the target vegetable. - Inherited sensitivity to bitter tastes was assessed using a forced-choice psychophysical test designed for children that took place in an individual interview after the 7-week period of exposure to the raw vegetable. - Children’s familiarity with and liking of the dressings used in the experiment were assessed before the exposure trials. |
| (Garcia et al., 2020) | - Intervention completed over 4 weeks - Prior to week 1 the CEBQ 6 questions were completed. - BCLC Intervention programme 4 times 1.5-hour sessions - Parent and children work together to prepare a meal in fun environment - Children encouraged to play with cooking utensils - Repeated exposure sensory learning - Encouraged to try the veggies particularly less common ones. |
| (Gomes et al., 2018) | - The ‘Red Apple’ programme included four sessions, each focusing on one major theme related to the young child’s growth process, nutritional guidelines and parental strategies to promote children’s healthy eating behaviours. - The approach was behaviourally focused, promoting the participation of the parents and supported by everyday examples. - At the end of each session, a theme related homework assignment was proposed, suggesting adult–child activities between sessions to apply the strategies discussed in the session and inviting caregivers to reflect on the benefits of applying the suggested changes. - A week after each session, parents received a newsletter, reinforcing the key messages of the previous session. - The intervention was delivered to fourteen groups, each one with six to eight parents. - Sessions were conducted in the schools in the presence of the class teacher and were scheduled to better fit parents’ preferences. - Each session lasted 90 min and occurred every two weeks. - The first author was responsible for the delivery of the intervention, with the participation of a dietitian in the second session to direct the nutritional education component. - Only those parents who participated in the first two sessions and in at least one of the sessions dedicated to parental feeding strategies were considered to have completed the programme and were retained for statistical analysis. - Minimal intervention and control groups Parents assigned to the MIG attended only a nutritional counselling session with the dietitian (similar to the second session in the CIG) and received the homework assignment and a newsletter related to the theme. - Parents included in the CG did not receive any intervention and completed the evaluation protocol |
| (Gripshover & Markman, 2013) | - In the intervention condition, books were read to children zero to two times per week for approximately 10 to 12 weeks. - Book sessions were integrated into the school’s routine of reading to small groups of children during snack time. - After children had heard each of the five books at least once, an experimenter conducted a 15-min structured interview to assess children’s grasp of each component of the intervention. - This interview included questions about food variety, nutrients, the dependence of biological processes on nutrients, and digestion. ach book emphasized one of five key concepts:   - dietary variety   - digestion   - food categories   - microscopic nutrients   - nutrients and biological functions - children were observed at snack time before and after intervention - children were served fruit cheese and crackers by teachers and invited to select as much as they want. |
| (Halbeisen & Walther, 2021) | - Experiment 1. This study was conducted with individual children. - Initially children were asked to name a set of 5 fruits (orange, banana, pear, kiwi, plum) and 5 vegetables (cucumber, carrot, corn, pepper, tomato). - For each child one fruit and one vegetable were selected as the two controlled stimuli. - Next was the conditioning procedure, which consisted of 16 randomly ordered trials. - The trials started with a blank screen, the experimenter then prompts an animation of rustling bushes, which lasts between 1.5 and 6s. - After these one of two stimuli pairings were presented for 3s. - The pairings were either the conditioned fruit or vegetable and an unconditioned stimulus. - There were two different types of unconditioned stimuli – ones that are likes such as a puppy and ones that are neutral. - It is thought that the conditioned foods would be liked more when paired with the unconditioned liked stimuli. - The pairings were randomly counterbalanced. - Food liking was measured using 10 heart shaped pieces of foam. - If the stimulus was liked more it received more hearts. Following this food choice was assessed using a toy grocery store. - Children were instructed to fill 5 slots with any item from the store that they would like to choose. - There were 10 possible foods to choose from. - Experiment 2. This study was similar to the first experiment, the unconditioned stimuli were changed here from liked pictures such as a puppy to stick figures that were either happy or neutral stick figures of a boy and girl. - The three main differences were instead of food choice- food consumption as measured. - Children were given 100g of carrots and informed that they were free to consume as much as they would like whilst listening to an audio play 3.5 min. - Difference between weight given and weight remaining was measured. |
| (Harnack et al., 2012) | - In the six weeks following the run-in period, each of the experimental conditions were implemented at lunches served over two randomly assigned one-week periods. - The centre’s 13-week cycle menu was running its course over the six-week experimental period, hence meals items served differed from day to day during the experiment. - During each day of the control weeks, the usual traditional family style meal service approach to serving lunch meals at the centre was followed. - During usual lunch meals at the centre children are seated around tables, and each food item on the menu is passed around the table from child to child in serving bowls for self-service. - Centre policy dictates that children must take some of each food item on the menu. - The amount taken, however, is the child’s choice. - Also, the amount eaten is up to the child (leftovers are allowed). - Self-served additional servings are allowed for all menu items with the stipulation that an additional serving of an item may be taken only if the first portion of that item has been completely eaten. - The meal period ends when a child indicates he/shines through eating. - There is no predetermined meal end time. - At the centre teachers are encouraged but not required to sit and eat with the children. - During the fruit and vegetable first experimental weeks all fruits and non-starchy vegetables on the lunch menu were served traditional family style five minutes in advance of other menu items. - Children were allowed to begin eating the fruit and vegetable items served first, with the remaining menu items (e.g., milk, entrée, side dishes) placed on the tables for traditional family style meal service five minutes following distribution of the first course. - All other usual meal service practices remained the same during the fruit and vegetable first experimental condition. - During the provider portioned experimental condition, a plate was prepared for each child that contained a specific quantity of each menu item. - The amounts served were generally consistent with guidelines for children 3–5 years of age:3/4 cup milk; 1 cup juice, fruit and/or vegetable; 1/2slice bread, 1/4 cup pasta or rice; and 1.5 oz meat or meat alternative. - After the plates of food were distributed, children were told to raise their hand if they had finished an item and wanted more of it. - The classroom teacher served a full additional serving of items for which a request for more was made. Like during usual meal service, the amount of food eaten was up to the child (leftovers were allowed). - During each meal service condition classroom teachers held primary responsibility for providing meal-related instructions to the children, distributing/serving food, and supervising the children during the meal. - During the provider portioned experimental condition a study staff member assisted the teachers in preparing the plated meals. - Assistance was provided to accommodate the implementation of this more labour-intensive meal service approach. |
| (Hoppu et al., 2015) | - In the intervention 5 different food education sessions were planned. - These were carried out once per week for 5 weeks and lasted approximately 20-30 minutes. - These activities activated all 5 senses, for example smell was activated by odour bottles. Pure taste was administered in water solutions. Touch was activated by having different foods in opaque fabric bags. - The concentration was on vegetables and berries, this included,  Carrots, cabbage, swede, rucola, romaine lettuce, and bilberry, sea buckthorns, and lingonberries. - Foods were also investigated using a magnifying glass. - In addition to the activity sessions the children were also asked to attend a session separately to measure tasting at baseline, and after the intervention. |
| (Hughes et al., 2012) | - The intervention is part of an assessment of the government scheme the School Fruit and Vegetables Scheme. - The scheme is set up in the first 3 years of primary school with children receiving a free piece of fruit and vegetable each day during school. - The aim of which is to promote part of the 5 a day goal of the government. |
| (Johnson et al., 2019) | - The Food Friends – Fun with new foods pre-school program designed to address obesity by promoting healthful eating – novel foods in particular. - Teacher delivered – 24 sessoins in total 15-20 minute sessions twice a week for 12 weeks. - Offered 1 food per week – repeatedly offered target food – Jicama, - Intervention group receives bilingual home materials and the control receives normal curriculum. - Eight food friends characters representing different food groups, activities include a puppet show, a tasting party, and puzzles, Low intensity booster program delivered during follow up years. - Tasting game: Taste 9 foods (Jicama, garbanzon beans, grapefruit, gouda cheese, couscous, spinach, salmon, beets and pineapple. - Food attributes considered during tasing. Children asked to rate each food at first taste as either “Yummy”, “Just OK”, or “Yucky”. |
| (Jones et al., 2015) | - Development of written nutrition and physical activity policies. - Staff monitoring of children's lunchboxes every day against written nutritional guidelines and provision of feedback to parents when a non-compliant food was packed. - Provision of water or reduced fat milk (for children over the age of 2 years) only. - Staff role modelling of physically active play and healthy eating every day. - Staff provision of prompts and positive comments to children to encourage physical activity and healthy eating every day. - Provision of adult-guided fundamental movement skill development activities every day for at least 75 % of children. - Restriction of sedentary screen time to less than weekly |
| (Joseph et al., 2015) | - The study intervention included one module entitled “Eat Smart”. - This taken from a larger interdisciplinary childhood obesity prevention programme that has previously been tested. - The focus was teaching children and empowering them to distinguish between a healthy and an unhealthy snack. - Healthy snacks were labelled ‘Go’ and they were shown as foods that were healthy because they help your heart, muscles, and bones to stay strong. - Unhealthy foods were labelled ‘Whoa’ foods and were shown to have the opposite effect. - A registered dietitian came to the school 4-5 days a week over a two-week period to give a 30-minute lesson. - Classroom extension materials were given to the teacher. - Newsletters with supporting activities were also sent home to the parents. - Snack choice was assessed through direct observation during snack trials scheduled immediately before and after the full nutrition education intervention. - Snack options were a cup of grapes cut into half or two small chocolate chip cookies in a similar cup. |
| (Karagiannaki, Ritz, Jensen, et al., 2021) | - During this visit, the children were served 100 g of round sliced daikon in a pre- weighed plastic beaker as an afternoon snack, sitting all together. - The subjects were instructed to eat as much as they liked and ask for a second serving of 100 g if they wished (maximum intake 200 g), while they were not allowed to share their beaker or comment on the food. - In addition, it was possible to accompany this snack with water or milk. - After the children finished eating the vegetable, the amount consumed was calculated by weighing the beakers on a scale with 0.1 g precision. - The conduction of the exposure visit followed the very same procedure as the intake visit, with the beakers this time containing 100 g of grated daikon and without offering a second serving (maximum intake 100 g). - The children were provided with a child-sized fork in order to eat the grated vegetable, and the headspace within the serving beakers was refreshed prior to serving to minimise smell. - After each exposure visit, the beakers were weighed again on a calibrated precision scale to calculate the amount of the vegetable eaten by the children. |
| (Karagiannaki, Ritz, Andreasen, et al., 2021) | - All vegetables were delivered prepared. - The different shapes were already processed and they were checked for brown colouring. - The pre- and the post intervention test were operated in the morning—between 9.00 and 11.00—thus some samples were prepared the previous day. - The exposure visits took place in the afternoon and the samples were prepared the same morning. - A quiet area of the kindergarten was chosen in order to provide a familiar and comfortable environment for the children during the tasting sessions. - Test sessions were separated into two parts: individual testing and assessment of intake. - The individual testing took place on a one-to-one basis with one child per assistant. - Children were instructed on how to use the scale as well as to just taste the vegetable and not eat it all during the tasting procedure. - Familiarity was tested by asking the children if they had tasted the vegetable in question before. - When liking was measured, the assistants encouraged the children to taste a piece of the vegetable and to indicate their liking by pointing to a smiley face on the scale. - The same procedure was repeated for all seven vegetables, with the use of water as a palate cleanser in between of each sample. - The dummy vegetables (cucumber and celery) were presented first, while beetroot and daikon were always served last. - The children were served a slice of each of the seven vegetables towards evaluating liking, and then their preference was measured for the three different shapes of beetroot and daikon. - The three different shapes (triangle, grated or sticks) of beetroot or daikon, according to the random order of serving, were presented to the child on a plate, requesting to taste all three styles and select the one he/she liked the best. - After the selected one was removed by the assistant from the plate, the child was asked to rate the remaining two styles without allowing any ties. - In case of refusal of a child to taste a vegetable, there was tried to rank the three styles only by appearance, without forcing the child to try. - Intake was measured in the control sessions by weighting the individual amount of vegetable left for every child using a precision scale. - Children were invited to eat as much, or as little, as they wanted, and they could also be served with another 100 g of the vegetable if they wanted. - The six intervention kindergarten teams were visited seven times during the intervention. - Children received a small plastic beaker with daikon in sticks, triangles or grated, according to each intervention group. - The grated daikon was served along with a child-sized fork, and headspace in serving beakers were refreshed to minimize smell. - During the exposure days, children received 100 g of the vegetable but not the possibility of a second serving. |
| (Kaufman-Shriqui et al., 2016) | - The IArm received the full program, which included nutrition-related intervention and PA - The CArm received PA lessons only. - All the lessons were delivered by professional personnel. - Structured lessons to the parents were delivered by clinical dietitians, and economists specializing in family budget planning. - Physical activity sessions were delivered by physical activity teachers. - The program emphasizes healthy affordable nutrition using traditional and ethnically accepted diverse nutritional recommendations. - Briefly, the main themes were increasing the consumption of fruits, vegetables, and legumes; decreasing the consumption of high fat and high sugar foods; decreasing sweet - It promoted choosing healthy snacks, preparing healthy packed lunches, choosing a healthy breakfast, preparing nutritious low-budget meals, and increasing leisure time physical activity. - Mothers and children in the intervention group received structured lessons in those themes. - All children in the intervention group participated in 10 weekly nutrition sessions delivered by a clinical dietitian. Each lesson was 45 min long, using short lectures, stories, games, and songs to cover each topic. - Parents received a weekly newsletter that paralleled the information offered to the children the same week. |
| (Kennedy et al., 2014) | - Two packages of ACT were delivered to the children. - One consisted of 4 ACT-based mindfulness activities. - The second which is referred to as ACT PLUS, also gives a reward based on consumption of the targeted food. - The sessions took place two to four afternoons each week between 3:00 and 3:30 which was 15-45 minutes after the student’s afternoon snack. - All the students that were present in the class that afternoon took part in the activity. - Fruits, vegetables, and beans were used in this study. - ACT included 4 activities each time, Silent game; look feel smell; Picture time; and the name game. - These were all designed to introduce mindfulness, and to increase mindful attention towards the food. |
| (Kong et al., 2016) | - The intervention curriculum aimed to improve both diet and physical activity, and decrease TV viewing. - The intervention materials were culturally specific to the target population and considered the practices and beliefs of this audience. - Teachers delivered this curriculum two times/week for 14 weeks. - Weekly sessions were theme-based and included:   - a 20-minute lesson on healthy eating or physical activity   - a 20-minute interactive physical activity component. - Study staff supplied teachers with colourful puppets representing food groups and a CD containing two fully scripted exercise routines to aid them in curriculum delivery. - In many of the food-themed lessons, children prepared simple classroom recipes and participated in food tastings. - The interactive 20-minute physical activity component was intended to be led by teachers through live demonstration. However, the level of comfort that teachers had with the physical activity component was variable, so a CD was developed, enhancing the ability of the teachers to deliver the physical activity component of the curriculum with sufficient structure and dose. |
| (Kornilaki et al., 2022) | - Training, researcher support, and other learning resources were provided to the educators, and the control group educators were given the training upon the conclusion of the intervention period. - For the Cretan trial, two, 2-hour professional learning sessions were delivered by trained researchers to the nursery school educators. - The sessions aimed to build the educator’s capacity to develop age-appropriate educational curriculum activities that increased young children’s healthy eating habits, raised children’s environmental consciousness and provided physical activity opportunities, while engaging their popular culture interests. - During the training sessions, the educators discussed ideas of combining these knowledge areas and designed a series of educational interventions that they could implement in their classrooms. - Educators were invited to follow the four best practice principles when they delivered their curriculum:   - implementing the curriculum experiences in the morning when most cognitively alert   - use a combination of three identified play types –open-ended play; modelled play; and purposefully framed play   - conduct the curriculum activities 2–3 times per week for between 4 and 6 weeks   - use a range of real-life props as resources for the curriculum experiences. - At the conclusion of the second professional development session, the educators were asked to do their curriculum activities in their classes. - These curriculum activities comprise the intervention and were different across each intervention group in order to flexibly respond to the interests of the children in each class. - Researchers relied on the practice expertise of each educator as they delivered the activities to their students, however during the implementation period, researcher support via phone and email was provided. |
| (Kristiansen et al., 2019) | - The intervention was implemented from September 2015 to February 2016 - One immediate evaluation (follow-up 1) - one follow-up evaluation one year after (follow-up 2). - Multiple intervention components, presented in Additional file 1, were provided to improve children’s vegetable consumption both at home and in the kindergarten. - The kindergartens were encouraged to implement and change their food practices in accordance with their own plans. - Each kindergarten in the intervention group (n 37) was invited to a one-day inspirational course in September 2015 where the intervention components were delivered. - This course consisted of a brief introduction of the rationale for the study, a practical training in the kitchen making vegetable soup and vegetables with a dip in small groups under the instruction of a cook. |
| (Kristiansen et al., 2021) | - The intervention was implemented from September 2015 to February 2016 - One immediate evaluation (follow-up 1) - one follow-up evaluation one year after (follow-up 2). - Multiple intervention components, presented in Additional file 1, were provided to improve children’s vegetable consumption both at home and in the kindergarten. - The kindergartens were encouraged to implement and change their food practices in accordance with their own plans. - Each kindergarten in the intervention group (n 37) was invited to a one-day inspirational course in September 2015 where the intervention components were delivered. - This course consisted of a brief introduction of the rationale for the study, a practical training in the kitchen making vegetable soup and vegetables with a dip in small groups under the instruction of a cook. |
| (Kristiansen et al., 2020) | - During the direct observations of the children’s intake of fruits, berries, and vegetables, the children were offered sliced fruits and vegetables rather than whole pieces, - In addition to this a coloured picture sheet accompanied, the observational form to assist researchers in deciding the portion size. - Children were sent home with a card describing the fruits and vegetables that they had eaten. |
| (Lanigan et al., 2019) | - A 2 x 2 x 4 fractionated within subjects design was used for this intervention study - Two conditions RE and CCNP + RE - Three time points – Pre intervention, Post intervention and a one month follow up - 4 types of food tomatoes, bell peppers, lentils and quinoa - Children completed food liking protocol which was used to determine intervention foods. - Each child was assigned two intervention foods. - The intervention food was assigned to RE or CCNP + RE groups by coin toss. - Two days per week during the six-week intervention trained Ras operated tasting stations. - Intervention children visited the tasting station and were offered 1 food to taste. - Half of the participants received RE food at first session and the second session CCNP +RE. This was reversed for the remaining participants. - On the CCNP + RE day the RA integrated food specific phrases into the conversation 2 times as the tasting was conducted. - ON the RE day the RA engaged in general non-food conversation only.  Liking data was collected at the food station. |
| (Larsen et al., 2017) | - The goal of the program was to encourage children to eat healthy foods, rather than focusing on foods that should be avoided. - The BHM program included 8 units implemented by classroom teachers between October and March of the 2013-2014 school year.   - Unit 1 provided an overview of the 5 food groups   - Units 2-6 focused on each food group   - Unit 7 focused on healthy snacks   - Unit 8 focused on healthy breakfast. - Each unit included 4 core learning activities, and up to 4 supplemental activities. - Teachers were encouraged to complete at least 3 core activities for each unit. - The BHM program also included a teacher’s guide with instructions and lesson plans, a poster of nutrition information, a box of food pictures, a student workbook, and family homework to build on concepts taught during the units. - The curriculum was based on an integration of the Health Belief Model and Social Cognitive Theory. |
| (Lee et al., 2017) | - The SAGE curriculum was implemented in participating enters twice a week in 1-hour sessions. - Children participated in 12 one-hour sessions that included songs, games, and interactive learning activities involving gar-den maintenance and taste tests. - Research assistants were paired and trained to deliver the intervention and were taught class-room management skills. - All research assistants attended weekly meetings with the project director and principal investigator in order to give a report on pro-gram delivery and to discuss any challenges and potential barriers. - Weekly newsletters developed in consort with our CAB were sent home with the children and used to engage parents in the program. - Newsletters were available in English and Spanish and included information about what was going on in the classroom that week, to keep parents engaged and knowledgeable about SAGE. - Newsletters also offered easy ideas for culturally tailored home activities and recipes, along with highlighting community resources that promoted PA and F&V consumption such as farmer’s markets or local gardening activities. |
| (Leis et al., 2020) | - The HSDS intervention was delivered over the course of 6 to 8 months - It included a 3-h on-site training, re-sources (i.e. an implementation manual, physical activity and healthy eating manuals, an active play equipment kit), and on-going on-line and telephone support and monitoring - centres were also offered a tailored 90-min booster session at the midway point of the intervention period. - ECC randomly allocated to the control group continued their usual practice and were not provided with any training, resources or support. - However, once the study was completed, all childcare centres from the control group were offered the HSDS intervention. |
| (Lim et al., 2016) | - 6 week programme adapted from the NASA MX for 5 year old children - Original intervention for 8-12 year olds - Modified for younger age group. - 6 modules   - four physical activities   - 2 nutrition sessions - Close room and home plan - Weekly Class session 40-60 minutes - Teacher and nutrition specialist lead - Focus on how to maintain healthy balanced diet and also explained what astronauts eat whist on the international space station. - Movie clips, food balance wheel, wearable fat vest. - Healthy vs unhealthy snack board. - Second session food arranging activities and educational games - Children wore fat vest to experience weight gain. |
| (Lumeng et al., 2017) | - Interventions included complementary but separate activities for and children (during school). - Transportation and childcare were provided as needed. - Both parent and child components were delivered from October to January by a master’s-level nutrition educator, who received 1.5 days of training, in collaboration with the classroom teacher, who received 2 hours of training. - The preschool classroom component consisted of 6 lessons - The parent component implemented by the nutrition educator consisted of eight 75-minute lessons that incorporated a cooking activity and focused on building knowledge and self-efficacy, as well as developing and practicing skills and strategies. - The IYS is an evidence-based program that emphasizes positive behavioural management techniques and enhances self-regulation in young, low-income children. - The IYS uses observational learning and reinforcement techniques and emphasizes behaviour-change strategies such as descriptive commenting about child behaviour, role-plays, and coaching to encourage and model positive behaviour. - The preschool classroom component consisted of 60 lessons followed by smaller group activities that addressed self-regulation skills, problem-solving strategies, and prosocial behaviour. - The parent component consisted of lessons delivered by using video vignettes in 14 group sessions or 10 home visits that were reinforced with homework and follow-up phone calls. |
| (Maimaran & Fishbach, 2014) | - In these studies, an experimenter read preschoolers a picture story in individual sessions. - The story featured a girl who had some food for a snack (crackers or carrots). - Depending on the experimental condition, the story either stated or did not state the benefits of the food.   - The first two studies test whether preschoolers consume less (studies 1 and 2), rate as less tasty (study 2), and are less likely to choose for future consumption (study 1) crackers that were presented as instrumental to being healthy, compared to tasty and neutral frames.   - Studies 3–5 test the prediction that using non-health messages.   - Study 3 tests whether presenting eating baby carrots as instrumental to knowing how to read reduces planned consumption of the carrots, compared to when the carrots are presented as tasty or with no message.   - Study 4 tests whether presenting eating the baby carrots as instrumental to knowing how to count reduces actual consumption compared to a neutral frame.   - Study 5 tests whether the effect of instrumentality framing on reduced consumption generalizes to stories in which the main character engages in instrumental activities after eating (e.g., go to school). |
| (Maimaran & Salant, 2019) | - Study 1. Children were offered to play with Legos, and it was manipulated whether they were told they had limited time to play or not. - The time children played served as our measure of engagement. - The children were then offered a snack to eat (carrots), and it was manipulated whether or not they were told there is a limited number of carrots. - Two experimenters, blind to the research hypotheses, collected the data. - Study 2. To test whether children indeed prefer crackers to carrots, a pretest with a separate group of 30 four-to five-year-old children from the same preschool was conducted. - These children were offered a choice between two snacks to eat:   - 12 petite baby carrots   - 12 crackers. - Study 3. To create limited availability in the context of choice, children were offered a choice between containers of grapes and containers of crackers - The frequency of containters in the choice set was manipulated. |
| (Marshall et al., 2020) | - For this study, participating families opted into the program at the start of the school year. - The food bank delivered weekly pallets of produce to the schools. - Since the program uses a co-op model, participating parents were invited to participate in produce bagging and distribution. - Produce pick-up was conducted at the end of the school day so that participating parents could pick up their child, the produce bags and taste the healthy recipe at the same time. - A Brighter Bites coordinator had a roster of the participating families to document attendance. - Distributions were conducted for 8 weeks each in the fall and spring semesters in each school. - All intervention schools implemented Brighter Bites and all comparison schools implemented CATCH only. - All participating schools were trained in CATCH prior to the baseline assessment. - A CATCH-only comparison school model was implemented to reflect a ‘real-life’ scenario since most schools in Texas implement health education as part of their curriculum. |
| (Mathias et al., 2012) | - Children and a primary caregiver visited an observational laboratory on Temple University’s Health Sciences Campus in Philadelphia, PA, once a week for 5 weeks. - The first visit was used to familiarize the children to the research setting, staff members, and the procedures. - Each child’s liking and preference of the foods on the experimental menu, anthropometric measurements, and caregiver self-reports were also assessed during this visit. - At the beginning of each visit, parents were asked to report any sleeping problems, illnesses, or medication taken by their child during the previous 24 hours. - Parents were asked to refrain from giving any food or beverages to their child 2 hours before arrival and to report any deviations from these instructions. - A trained staff member sat at the table during the meal to ensure that procedures were followed, including preventing children from sharing foods, noting dropped foods, and re-directing food-related conversation. - The children were given 20 minutes to eat dinner. - To minimize visual comparisons of portion sizes, all children in the same group were served the same experimental condition. - Children were instructed teat as little or as much as they liked. - Fixed portions of rotini pasta (204g; San Giorgio, Harrisburg, PA) with tomato sauce (106 grego, Traditional, Campbell Soup Co, Camden, NJ), 2%milk (Acme Markets, Supervalu Inc, Eden Prairie, MN) And a side of light ranch dressing (Hidden Valley, The Clorox Company, Oakland, CA) were offered in all conditions. - Only the portion sizes of the drained canned peaches in light syrup and cooked broccoli were manipulated. - Butter was added to match energy density. - Food intake of each type of food was recorded. |
| (Melnick et al., 2020) | - The Culture of Wellness in Preschools Nutrition Education (COWP) programme is a comprehensive obesity programme with five components.   - A classroom-based nutrition education component.   - A classroom-based professional development and coaching component.   - A staff workplace wellness programme.   - A parent wellness workshop series.   - A strategic planning process to make health-promoting policy, system and environment changes. - Participating schools received 12 lessons of the first component. - These 12 lessons involved a different theme each week and included different fruits and vegetables. - The lessons lasted 30-45 minutes. - During the lesson a developmentally appropriate book was read, a song was sung, and an experiential food preparation and tasting activity was included. - Letters were also sent home to parents with a healthy recipe included. |
| (Mohd Nor et al., 2021) | - Following a pre-intervention test of intake, children received 10 exposures (once/attended school day) of steamed-pureed turnip, after which it was offered once again at a post-intervention test. - The intervention itself was a repeat exposure intervention, exposing children to turnip once a day for 10 days. - A follow-up was done 3 months after postintervention to assess the durability of the effects of repeated taste exposure. - At pre- and post-intervention tests, Day 5, Day 8 of the exposure period and follow-up, children were given one pot of 100 g of steamed pureed turnip. - Children were individually taken out of their classes to a separate room. - They were asked to eat as much as or as little as they wanted. - No persuasion or force was used. - Intake and liking of the puree were measured at these times. For the rest of the exposure days (Day 1, 2, 3, 4, 6, 7, 9 and 10), only 1 teaspoon (approximately 5 g) of the puree was given, intake and liking were not measured, but refusal to eat was monitored. - At these times, children were taken out of their classes in groups of between 2 and 5 children. Intake was measured in grams (g) using a digital weighing scale (3 decimal places) - Liking was assessed using a 3-point hedonic scale. - PROP taster status was determined by using filter papers impregnated with PROP |
| (Morris et al., 2016) | - The six intervention educators were provided with a copy of the pedagogical communication strategy and a book titled ‘Young children’s play and environmental education in early childhood education’ (Cutter-Mackenzie, Edwards, Moore, & Boyd, 2014). - Using these resources, they were asked to develop a curriculum intervention that integrated content knowledge about healthy eating, active play and sustainability awareness. - The intervention educators also participated in three 1.5 hour professional learning sessions about digital media, popular-culture, health and environmental education in early childhood - Sessions were conducted by investigators with expertise in the delivery of professional learning programmes to educators and with content knowledge in the areas of play-based learning, environmental education, digital media literacy and healthy eating. - Educators subsequently planned, developed and implemented a series of learning experiences with the children attending their kindergartens from early August to mid-September, 2014. - The control wait list educators participated in an information session about the project and then continued their practice as usual. |
| (Morris et al., 2018) | - For Task 1, two placemats showing a large green circle and a large red circle were placed in front of the children. - It was explained to them that the green circle meant ‘Go, do or eat that’, and the red circle meant ‘Stop, don’t do or eat that’. - Children were then given each of the eight pictures in random order and were invited to consider placing them on either the green or red circle. - To reduce time burden of the assessment, children were not invited to share their reasoning for placing pictures on the red or green circles. - For Task 2, children were invited to indicate which of the three images they believed belonged together. - A ‘correct’ response was recorded as pairing the cup and bottles of milk. - The purpose of this task was to establish children’s capacity for knowledge connections in Task 3. - For Task 3, the eight images were randomly placed in front of the children. - They were invited to group any pictures they understood to belong together. - The researcher then invited the children to explain their selections. - After the explanation, the images were returned to the table. - Children were again asked if there were pictures, they understood to belong together and to explain these. - This process continued until the children indicated that there were no further pairings to create. - Each grouping of images was used to indicate children’s knowledge connections between wellbeing and sustainability. |
| (Naderer et al., 2017) | - The children participated in the movie presentation of the study in groups of up to 14. - To avoid social influences in the groups, all children were instructed to watch the movie carefully and not to speak during the cartoon presentation. - In the group situation, verbal and non-verbal cues that may influence the dependent variables were recorded. - The children were randomly exposed to one of three versions of the stimulus cartoon, with one experimenter supervising the screening. - After stimulus presentation, each child was individually interviewed. - As there were four experimenters, three children were simultaneously interviewed in separate interview rooms, while the experimenter supervising the screening stayed in the screening room with the remaining children entertaining them with a ballgame. - Researchers took each child to separate interview rooms and, as an alleged gesture of appreciation for them partaking in the study, gave them the option to choose a slice of one of the two provided snack options. - One of which was the embedded mandarin - The other one the embedded fruit gum - Each child was allowed to pick only one of the two snack options, with the order of snack presentation on the table (left or right) being fully randomized. - After noting the child's snack selection, the interviewer proceeded with the questionnaire. - Once all children from one class were interviewed, the children were extensively debriefed about the purpose of the study as well as healthy eating behaviour |
| (Natale et al., 2014) | - The intervention was split into 3 components. - The Teacher component – Modelled after a modified version of Hip-Hop to Health Jr (Hi-HO). - The teachers and staff were given two trainings per centre, and they were given lessons and strategies to use with the children. - The addition of weekly technical assistance was given to ensure implementation of a low fat high fibre diet including more fruits and vegetables emphasising cultural barriers. - Parent Component – Modelled on Eat right is Basic and Hi-HO. This included a monthly educational dinner discussing nutrition and physical activities. - Monthly newsletters and at home tasks. - Centre-Based Modifications Component. Childcare centre modifications included a nutritionist led change to the menu for each centre. |
| (Natale et al., 2017) | - Healthy Caregivers–Healthy Children implemented the following policies in all intervention child-care centres:   - drink policy; water promoted as the primary beverage for staff and children, all cow’s milk provided will contain <1% milk fat, and juice limited to 1 time per week   - snack policy: snack and meal time incorporate fresh fruits and/or vegetables daily   - physical activity policy; physical activity encouraged for > 60 min/d   - screen time pol-icy; TV/videotape/computer viewing logged and limited to<30 min/week.   - Parent and teacher role-modelling curriculum. - The curriculum used the nutritional gatekeeper concept with a theoretical foundation in social cog-native theory (SCT). - The goal was to for parents and teachers set a healthy example to instil beneficial lifestyle habits for their children and students as well as for themselves. - The HC2 program staff conducted joint parent–teacher meetings that focused on an evidence-based nutrition and physical activity curriculum. - Study team members taught parents and teachers about   - choosing healthy foods, emphasizing increased fruit and vegetable consumption,   - consistent healthy food choices,   - preparing nutritious snacks,   - preparing new recipes,   - tasting new foods,   - learning about food safety and storage,   - planning meals,   - making grocery lists,   - shopping wisely for groceries,   - budgeting food dollars and food stamps,   - using food labels to inform buying decisions. - parents and teachers were encouraged to implement change at the home and childcare centre. - To increase the ease of use, the lesson plan outlines included cognitive, fine motor, and self-help instructional com-ponents required for teachers as they apply to the policy objective. - During these weekly visits, curriculum specialists targeted the cognitive, cultural, and environmental barriers to consuming a healthy diet and increasing fruit/vegetable consumption. - These weekly visits were designed to increase self-efficacy to pro-mote and sustain positive dietary changes and increased physical activity. - The child curriculum was implemented weekly throughout the school year |
| (Nederkoorn et al., 2018) | - Children were manually exposed to the tactile stimuli during individual sessions. - Children were asked to play with a large bowl filled with a colourless and odourless jelly - In the beginning, the children were free to play with the gelatine mass, as long as they used their whole hands in interaction with the jelly. - After a few minutes the children were motivated to feel the texture by tasks like scooping the jelly with their hands into another bowl, finding hidden coins in the bowl and sculpting figures from the jelly - When children were shy or reluctant to touch the jelly during the free play, these tasks were initiated from the start. - This way, the experimenter made sure that all the children touched the jelly for 10 min, in a playful way. - In the control group, children played an age-appropriate boardgame (Memory) with the experimenter, for 10 mins. - Children were asked to eat from three types of strawberry dessert, in balanced order:   - Yoghurt with pieces: a pink colored strawberry yoghurt (low-fat) that contained small pieces of strawberry   - Smooth yoghurt: the same yoghurt, filtered to remove the pieces of strawberry, resulting in a smooth strawberry yoghurt   - Jelly: arid coloured strawberry jelly pudding - The desserts were presented in equal clear plastic bowls - The children were asked to taste a spoonful of the food and if they tried it, the experimenter asked if they would like another spoon. - The children handled the spoons themselves, the experimenters supervised if the spoons were properly filled and helped the younger children when needed. - The number of spoons of a dessert a child ate was considered an index of acceptance of the food. - Children were not pressured to eat if they did not want to. - They could eat 0 to 3, an average spoon contained 5 g of dessert |
| (Nekitsing, Blundell-Birtill, Cockroft, & Hetherington, 2019) | - The preschool staff members were provided with all the necessary resources and basic instructions to deliver the intervention to children in their preschools. - During Week 1 baseline intake of mooli was measured at the prearranged snack time and children’s height and weight was also measured. - Over the next 10-week children in the intervention conditions were offered either the TE, NE, or TE þNE intervention - children in the control condition were offered no intervention - After the intervention at Week 12 (postintervention) mooli intake was measured at snack time. - Mooli intake was also recorded at two follow-up periods (Week 24 and Week 36) at the usual snack time. - The intervention was delivered at the level of the preschool and outcomes measured at the individual level. |
| (Nekitsing, Blundell-Birtill, Cockroft, Fildes, et al., 2019) | - The preschool staff were provided with all the necessary resources and some basic instructions to deliver the intervention to children in their classrooms. - On the first day of the intervention, immediately after the baseline vegetable recognition test and intake assessment, the allocated storybook was read to the children. - The children in the two congruent (target) conditions were read the celeriac story - children allocated to the incongruent (control) conditions were read the carrot story ( - Children who were also allocated to the sensory play conditions were encouraged to explore and play with the respective vegetable. - Over the next 14 days (9 preschool days) staff were requested to keep the storybooks on the clear acrylic stands provided to increase visual exposure and to read their designated storybooks a minimum of five times. - The staff were also asked to keep a register of attendance so that children who were absent during the story times could be identified. - On the final day of the intervention, procedures of the first day were repeated. - This was immediately followed by a post-intervention vegetable recognition test and intake assessment. - The researcher was present to observe preschool staff on days 1 and 15, as well as several interceding occasions, taking notes on delivery and compliance with the intervention. - The story session lasted between 5 and 12 minutes, depending on the children’s age, attention span, and interest in the story. - Parents were given questionnaires to take home from preschool. - Parents were asked to report their child’s usual intake of carrot and celeriac over the last month using ratings on a 9-point scale that ranged from never or less than once per month to six plus per day, - however, for some children the preschool staff member completed the questionnaire |
| (Nicklas et al., 2017) | - The intervention was conducted in 11 classrooms at three schools in three HS districts for four consecutive weeks to accommodate the series of four puppet shows. - three professionally developed characters, unique storylines and an engaging, repetitious song were incorporated in four 20-min videotaped puppet shows. - Prior to lunch each show was shown for five consecutive days in HS and a minimum of once in the home. - On Mondays each intervention child took home a bag including the DVD video for that week, a pamphlet, main ingredients to prepare a simple vegetable snack, crayons, and a disposable camera to use as instructed in the booklets. - The pamphlet materials included positive feeding practices, instructions on snack recipe preparation, content information contained in the videos, in-home instructions on taking and sending pictures of the children preparing and tasting the snack, and questions for parents about the video content. - The questions on the video content could be answered correctly only if the parent had watched the video. - On Mondays of weeks two, three, four,and five, researchers gathered from the teachers the booklets that were returned by the parents from the previous week. - As an incentive, each time a family returned a completed pamphlet, they were entered into a drawing to have a professionally taken family portrait |
| (Nyberg et al., 2015) | - The intervention was comprised of three components - Health Information   - A brochure was developed to increase parental knowledge on how to promote healthy dietary and physical activity habits. - Motivational interviewing.   - Designed to increase parental care and control, and self efficacy to provide support for healthy eating and physical activity to the child. Parents in the intervention group were provided with two sessions without the presence of the child. Each session lasted 45 minutes. - Classroom activities,   - Aimed to target children’s knowledge, attitude and preferences and the parents’ role modelling for healthy behaviours.   - Activities were directly related to the brochure given to the parents. Focusing on the importance of eating fruits and Vegetables.   - Each session lasted 30 minutes.   - Children were given homework workbooks to discuss the sessions with their parents at home. |
| (O'Connell et al., 2012) | - Each day, raw vegetables were purchased, washed, cut into bite-sized pieces, and placed in a snack-size plastic bag with student identification. - All bags were weighed to the nearest gram and labelled. - The servings (24 to 26 g) were consistent with recommendations for this age group - At lunch, all children were served the same meal and the teachers passed the vegetable bags to the children as part of serving the meal. - All children were assigned a lunch table and ate at the same table each day. - The preschool directors at both schools required the research study vegetables to be served in addition to (and not in place of) the produce in their Child and Adult Care Food Program reimbursable lunches. - Teachers were instructed not to exert any pressure on the children to eat the vegetables - however, they were encouraged to discuss what they were eating - At the end of the lunch period, the teachers instructed students to put any waste back into the bags. - Researchers picked up the bags later from the schools, weighed them, and calculated intake to the nearest gram. |
| (Olsen et al., 2019) | - The study consisted of seven visits for each nursery group - two sessions scheduled per week. - At the first visit, hedonic evaluations were conducted, and the remaining six visits included choice versus no choice conditions. - The data collection was conducted by trained assistants. - The vegetables were served as an afternoon snack at the time the children were used to eat a snack. - Children were instructed not to comment on the food and not to share any food. - They were eating together in their usual groups to make the servings as natural to the children as possible. - During visits 2–4, children were served one type of vegetable only:100 g of either sugar snaps, snack carrots or baby corn. - During visit 5,children were offered 33 g of each of the three vegetables (the no choice condition with a mix of stimuli). - During visit 6, children were free to choose a single stimulus (referred to as one type of vegetable) from three different vegetables, - Visit 7 they were free to choose from a mix of two vegetables (each 50 g) with three alternatives. - Intake was assessed by pre- and post-weighing all types of vegetables in all serving containers, and to use markings on the containers. - All visits with no choice were scheduled prior to visits with choice. |
| (Pathirana et al., 2018) | - Four Structures cooking - four structured physical play sessions - each lasting 1-2 hours - delivered weekly by locally trained facilitators - over a period of 8 weeks in Playgroup settings. - No further details provided in study; the intervention website is no longer available. |
| (Pinket et al., 2017) | - the ToyBox-intervention targeted four key behaviours related to early childhood obesity:   - water consumption,   - snacking behaviour,   - physical activity,   - sedentary behaviour. - During the first level of implementation, kindergarten teachers made environmental changes in the kindergartens. - In the second implementation level, kindergarten teachers promoted the four key behaviours on a regular basis, for instance by reminding preschoolers to drink water regularly and by arranging a daily break for the whole class to eat healthy snacks. - In the third level of implementation, kindergarten teachers implemented fun classroom activities, such as kangaroo stories, experiments, games, and excursions, for a minimum of one hour per week. - The ToyBox-intervention materials were provided to kindergarten teachers in a box. - This box contained a teacher’s guide with general information on the ToyBox-intervention and the importance of the four key behaviours, a classroom activity guide for each key behaviour and a kangaroo hand puppet. - The classroom activity guides consisted of three sections, matching the first three levels of implementation: setting environmental changes in the classroom, preschoolers implementing the actual behaviour, and teachers implementing fun classroom activities. - The fourth level of implementation targeted parents/caregivers. - Preschoolers received nine newsletters, eight tip-cards, and four posters to take home. - The third level of each key behaviour was focused on for four weeks, followed by a repetition period in which each key behaviour was focused on for another two weeks. - Prior to the intervention, teachers were invited to two teacher training sessions in which researchers explained the ToyBox-intervention and provided detailed information on how to implement the materials. - A third teacher training session was scheduled before the repetition period. |
| (Piziak, 2021) | - Two games were included, - A Bilingual Nutrition Game was developed in the form of a pictorial Bingo to be played by adults and children. - When the card is displayed the teacher is to give nutritional information about that item, including many vegetables. - Includes also information regarding how often that it is wise to eat soda or candy. - The second game was a video nutrition and exercise game. - The children had to design their own characters, so that children would bond to them. - Through use of a dance pad it was possible to measure the number of steps a child took when playing on a game. - The nutrition element was designed to encourage the intake of healthy foods, high in fibre and low in sugar. |
| (Ray et al., 2020) | - In intervention preschools, all early educators received program training. - The training was split into a longer training session after the baseline measurements and a shorter training session around the middle of the 23-week program, in all, approximately 8 hours - Throughout the intervention, two researchers engaged with early educators conducting the program by email. - The program was run in both preschools and homes and divided into five themes, all of which lasted 4–5 weeks:   - SR skills   - physical activity   - fruit and vegetables   - screen time   - sugary foods and beverages. - SR skills along with each EBRB were emphasized throughout the program in the preschool activities. - SR skills were promoted by brain breaks, which were a few minutes’ calming down and breathing sessions three times per day, led by early educators. - In addition, early educators were trained to teach children to recognize and reflect on different feelings. - In the family activities, focus was set on the children’s EBRBs, and on how parents, by acting as role models and changing the availability and accessibility of the home environment, could influence their children’s EBRBs. - The methods used for families were, among others, information letters, emails containing videos or articles, bingos related to EBRBs, and two fairy tales written for the project. For each of the five themes, preschools arranged one activity afternoon. |
| (Rioux et al., 2018) | - This 4-week study consisted of three phases: a visual exposure phase where place mats were set on tables in the cafeteria of the three schools participating in the study, and pre and post inter-venation phases where Willingness to Try Vegetables (WTV) as well as cognitive performances were assessed for each child - In the 2-week intervention phase, the three schools participating in the study were randomly assigned to one of the three experimental conditions:   - simple exposure condition (n2 ¼ 24; 13girls and 11 boys, mean age ¼ 52.21 months, SD ¼ 9.45),   - diverse exposure condition (n3 ¼ 26; 17 girls and 9 boys, mean age ¼ 54.08months, SD ¼ 8.08)   - and control condition (n1 ¼ 20; 10 girls and 10boys, mean age ¼ 46.56 months, SD ¼ 6.25). - In each of the three schools, place mats were set every day on cafeteria tables, for two consecutive weeks, therefore children were exposed to these mats eight times (as cafeterias are closed on Wednesdays). - Each place mat was printed on a laminated card measuring 21 x 29.7 cm and contained five colour pictures. - The five pictures printed on the mats depended on the intervention condition assignment. - Simple exposure condition (SE). In this school, the same placemat with five unfamiliar vegetables was presented to children eight times. - the vegetables were: green tomato, purple cauliflower, white beetroot, yellow bell pepper, and purple carrot - Diverse exposure condition (DE). In this school, place mats presented the same kinds of vegetables as in the previous condition. - However, contrary to the simple exposure condition where one vegetable was always presented in the same colour, in this condition each of the five vegetables was presented in four different atypical and unfamiliar colours - Control condition (C). In this school, the same place mats with pictures of five stones were presented to children - The five stones were selected to match for unfamiliarity and colour diversity with the vegetables printed on the mat of the simple exposure condition |
| (Roberts et al., 2022) | - Three exposure conditions were used. - the researcher introduced the activities as follows, “Hello, we are going to play. a matching game with vegetables. Here is our vegetable matching poster and inside these boxes are some vegetables. Let’s see if you can match them to the poster”. - The researcher then placed one of the containers containing a vegetable on the table and the matching game would begin. - When the game had finished with that vegetable the next container was placed on. the table, and this process continued until all six vegetables had been exposed. - Vegetables were presented in random order. - Visual only - As each container was presented, the researcher removed the cloth surrounding the container and asked the child to point to the picture on the poster that matched the vegetable inside. - The researcher then asked follow-up questions to encourage visual exploration: “What does it look like?” “What colour is it?” If the child correctly matched the vegetable to its image on the poster, the researcher congratulated the child and labelled the vegetable, before. moving onto the next container. - If the child answered incorrectly, the researcher asked them to try again, before correctly identifying which. vegetable it was. - Smell-Visual. As each container was presented, the researcher. removed its lid and asked the child to smell the vegetable through the cloth, asking, “What does it smell like?” and encouraging the child to try. to match the smell to one of the vegetables on the poster. - If they guessed correctly, they were congratulated. - If they guessed incorrectly, they were asked to have another guess. - The cloth mesh was then removed so that the child could see the vegetable, and the child was asked again to match the vegetable to the poster, as in the Visual-only condition. S - Smell-Tactile-Visual. The researcher first conducted the ‘smelling’. matching game as described for the Smell-Visual condition above. - She then released the cloth mesh covering the container just enough to allow. the child to slip their hand in to feel the vegetable. - The child was asked. to guess which vegetable, they were feeling by pointing to the matching picture on the poster. - Finally, they were invited to see if they had. guessed correctly, at which point the mesh and cloth cover were fully. removed to allow the child to see the vegetable in the container. - The child was then asked to match the vegetable to the poster, as described. in the Visual-only condition. - Control. The procedure for children in the Control condition was identical to that in the Visual-only condition, except that child were asked to match six non-food items in containers to the pictures of these on a poster. - For each item, the researcher said: “Look what is in the container. Can you point to the matching picture on the poster?” - The second exposure session was identical except that it involved new exemplars of the same items. - Taste test The taste test was the same for children in all four conditions. - Each child was presented with a tray that included two prepared pieces of each of the six vegetables. - Those in the vegetable exposure conditions were told that these were the same vegetables they had seen in the matching game. - The researcher labelled each vegetable in turn while matching it to its picture on the poster. - The child was asked if they would like to taste the vegetables, and which one would they like to try first. - Children were given time to taste the foods at their own pace and were encouraged to taste as many as they liked. - Once they had clearly stopped engaging with the vegetables, the researcher asked if they had finished and cleared the tray away |
| (Roe et al., 2013) | - The test foods in this experiment were 3 types of vegetables and 3 types of fruit, prepared - The vegetables and fruit were chosen from among those that had previously been served at the childcare facility and that could be cut into uniform pieces - Compared with the vegetables, the fruit had a higher content of sugar and energy and a lower content of water - The vegetables and fruit were served in uniform pieces with an average weight of 10 g/piece and an allowable range of 8 to 12 g/piece; uniform pieces allowed observers to assess individual intakes of the children participating in the study. - The vegetable and fruit snacks were typically served as a variety of 2 or3 types, but occasionally a single type was served along with a grain-based item such as bread. - The afternoon snack was provided at a standard time in each classroom after either a nap or a period of quiet play. - At snack time, the children sat in their usual places in the same groups at the same tables. - Each of the 4 classrooms had 3 tables that seated 5–7 children and 1 adult helper. - Children at the table who were not participating in the study were provided with the same snack, but their intake was not recorded. - The number of studies participants in each of the 4 classrooms ranged from 9 to 19 children. - In accordance with the usual practice in the classrooms, vegetables and fruit were served family style at each table. - The tables were provided with 3 serving bowls, each of which contained 300 g (30 pieces). In the variety conditions, each of the 3 bowls contained a different type of vegetable or fruit; in the single-type conditions, all 3 bowls contained the same type of vegetable or fruit. - The bowls were passed around the table, and the children used spoons or tongs to select as many pieces as they wanted and place them on their plates. - Extra serving bowls of vegetables or fruit were available in each classroom. - There was sufficient amount of food that all children had access to each type of vegetable or fruit. - All children were also provided with a small piece of pita bread (16 g; 43 kcal) and 250 mL water as a beverage, which were set at their places at the table before the snack began. - The adult helper at the table ensured that the serving bowls were passed around the table to each child, according to the usual practice at the facility, but did not eat any of the snacks. - The adult did not encourage or comment on the children’s selection or consumption of any food and redirected any of the children’s comments about food to other topics. |
| (Roe et al., 2022) | - In each experimental condition, the children were served with the same foods using the same 5 daily menus - Only the weights of the foods were varied to change the proportions of vegetables and fruits served at meals and snacks. - All vegetables and fruits on the menus were served separately, from other foods, either as side dishes at main meals or as part of snack. - In the Control condition, we served typical portions in all food groups, - For vegetables and fruits in the Addition condition, we increased the weight of all vegetable and fruit portions by 50% from Control amounts, without changing the other components of meals and snacks. - In the Substitution condition, we increased the portions of vegetables and fruits by 50% from Control amounts and decreased portions by an equivalent weight for meal components higher in ED - The Control portions of main dishes and grain-based and dairy snack foods were greater than the CACFP minimum amounts, to ensure that after reducing their portion sizes for the Substitution condition, the minimum amounts were provided. - Across all 5 menus, vegetables and fruits accounted for 40% of the weight of food served in the Control condition, 50% in the Addition condition, and 60% in the Substitution condition - On the Control menus, 1.5cups each of vegetables and fruits each day was served, and on the Addition and Substitution menus, 2.25 cups each was served. - The children ate breakfast, lunch, afternoon snack, and dinner at their childcare centres, - Following the standard practice in the childcare centres, participating children ate at a shared table with 3–9 other participants and 1–2 adults. - At each eating occasion in the childcare centres, children were served a plate with reweighed amounts of each food and a container of reweighed milk and were instructed to eat or drink as much or as little as they would like. - Children were informed that they would not be given additional servings of any of the meal components and that no alternatives would be provided if a child chose not to consume any items. - To minimize peer influence on intake, a researcher sat with the children at each meal and snack and redirected any food-related conversations. - If any food was spilled or dropped, a researcher returned it to the correct plate before the measurement of post meal weights. - Morning and evening snacks were provided for home consumption so that children had an opportunity to eat study foods if they were hungry either before or after their childcare session. - We instructed parents to allow only the study participant to consume the foods that were sent home and to return any uneaten foods in the provided containers. - We collected snack containers and any leftovers each morning at the childcare centre and weighed them to determine intake. - On each study day, we asked parents to complete a form describing any foods and beverages consumed by their child that had not been provided by the researchers. |
| (Rohlfs Domínguez et al., 2013) | - Each of the 4 primary schools were assigned to one of three groups:   - Discrete choice condition (DDC; n = 50) including choice at the beginning of the meal   - Continuous discrete choice plus variety condition (CDCP; n = 56), having two vegetables available during the meal   - no-choice condition (NCC; n = 44) receiving only one vegetable. - Children belonging to CDCP were given the opportunity to make a choice between two vegetables with high frequency and they could enjoy a variety of vegetables during the whole meal. |
| (Savage et al., 2013) | - Experiment 1 - Each child participated in two short tasting sessions, - tasting three vegetables (one liked, one disliked, and one refused) with a preferred herb dip and plain reduced-fat dip. - The vegetables, which had been previously identified and differed for each child and were in keeping with their preferences. - Half of the children were given the plain dip and half their favourite herb-flavoured dip in the first tasting session, and the order was reversed for the second session, with a 10- to 15-minute break between the sessions. - The child was told the names of the vegetables given, encouraged to taste the liked vegetable/dip combination first, and then allowed to choose which vegetable to taste next with the dip. - After tasting the combination, the child rated it as “yummy,” “just okay,” or “yucky.” - The order in which the samples were tasted and whether the sample was rejected were recorded. - Herbs and spices were added to a dip rather than directly on the vegetable because there was concern that the flavour principle present in the dip paired with a vegetable would differ substantially from the flavour principle of herbs and spices added directly to vegetables. - Experiment 2 - Each child participated in four snack sessions, administered on four different days, to assess the impact of dip condition (with or without the child's preferred pizza or ranch dip) on ad libitum intake of two vegetables. - Yellow squash and celery were chosen because parents had indicated that their children were unfamiliar with these vegetables at study entry and because these were the most disliked vegetables in the familiarity and liking session. - One condition was tested each day:   - celery with the preferred dip,   - celery without dip,   - steamed squash with the preferred dip,   - steamed squash without dip. - The order in which children received each of these conditions was counterbalanced, such that children were randomized to receive either yellow squash or celery first. - After each tasting session, the child rated the sample as “yummy,” “just okay,” or “yucky.” - A record was made of the order in which the samples were tasted and whether the sample was rejected. - Food weights to the nearest 0.1 g were recorded before and after consumption using digital scales - The amount of each food item consumed (in grams) was determined by subtracting the post-snack weight from the pre-snack weight. |
| (Serebrennikov et al., 2020) | - The intervention involved teachers of treatment class rooms implementing a 6-week, bi-weekly curriculum designed to improve students’ knowledge and preferences for fruits and vegetables. - Each lesson was 15–20 minutes long. - The teachers in the control classrooms taught regular curriculum without any specific nutrition education. - Teachers of treatment classrooms were trained by the co-authors in the activities and lesson plans involved in the curriculum. - Teachers of treatment classrooms received teaching material, schedule, manual, and all the supporting material for successful implementation of the curriculum. - Control classroom teachers received no such training or material. - The curriculum and the teaching material were designed by the co-authors and a group of 3rd grade teachers not involved in the intervention. - The curriculum was developed based on four existing programs:   - MyPlate Levels 1 and 2,   - Two-Bite Club,   - Put a Rainbow on Your Plate. - There were no other programs or interventions related to nutrition education taught to second grade students at any of the schools. |
| (Sharma et al., 2016) | - For this study, participating families opted into the program at the start of the school year. - The food bank delivered weekly pallets of produce to the schools. - Since the program uses a co-op model, participating parents were invited to participate in produce bagging and distribution. - Produce pick-up was conducted at the end of the school day so that participating parents could pick up their child, the produce bags and taste the healthy recipe at the same time. - A Brighter Bites coordinator had a roster of the participating families to document attendance. - Distributions were conducted for 8 weeks each in the fall and spring semesters in each school. - All intervention schools implemented Brighter Bites and all comparison schools implemented CATCH only. - All participating schools were trained in CATCH prior to the baseline assessment. - A CATCH-only comparison school model was implemented to reflect a ‘real-life’ scenario since most schools in Texas implement health education as part of their curriculum. |
| (Smethers et al., 2019) | - Different daily menus were served on each of the 5 d within each experimental period - On the menus there were 82 food items, selected to be familiar tithe children, plus milk as a beverage. - The menus in the 100% portion size condition met the minimum requirements of the Child and Adult Care Food Program. - For the other condition, the portions were increased by 50%, a difference that has been used in previous studies and that can be observed in the food environment (e.g., a 4- versus 6-piece chicken nugget meal). - Breakfast consisted of a grain-based main dish, fruit, and milk, with a protein food added on 2 d; lunch and dinner consisted of a grain- and protein-based main dish, vegetable, fruit, and milk; afternoon and evening snacks consisted of 2 of 5 snack components (milk, grain, protein, fruit, or vegetable) and the morning snack was a cereal bar. - Across all 5 menus, the mean daily energy served in the 100% portion condition was1627 kcal/d and the mean served in the 150% condition was2450 kcal/d. - These amounts were equivalent to 162% and 243% of the mean estimated energy requirements of preschool children, respectively. - The baseline amounts were greater than most children would be expected to finish. - Of the total energy served on all 5 menus, the 3 main meals provided 68% and the 3 snacks provided 32%. - The children ate breakfast, lunch, and afternoon snack at the regularly scheduled times in the childcare centre; dinner was served at the end of the childcare day. - Study participants ate at shared tables with 4 to 8 other participants and 1 or 2 adults, which is standard practice at the childcare centres. - At the meals, each participant was served a plate with reweighed portions of each food and a container of reweighed milk. - The children were told that they could eat or drink as little or as much as they wanted of the food and milk served but could not request more of anytime. - If children chose not to eat a food item they were not given additional food options, which is standard practice at the childcare centres. - During the meals, staff sat with the children and redirected conversations about food-related topics to minimize any influence on intake. - After the children had finished eating, researchers returned any dropped or spilled food to the correct plate before weighing the uneaten food items to determine the intake of each child. - Morning and evening snacks were provided for home consumption to ensure that if children were still hungry at the end of study day they had an opportunity to consume additional food. - The morning snack was for consumption before breakfast and the evening snack was for consumption after dinner. - These snacks were reweighed and placed in individual containers, which were packed in personal insulated coolers for transport. - Parents were instructed that these snacks were to be consumed only by the study participant. - The containers and any uneaten amounts of the snacks were returned to the childcare centre on the subsequent day and weighed to determine intake. - On the evening of each study day, parents completed a report to document whether the child had consumed any foods or beverages other than those provided for the study and to briefly describe any non-study items consumed by their child |
| (Smith et al., 2013) | - The programme supports families by providing information on child nutrition active play and parenting practices to help parents practically integrate these recommendations into everyday life. - The programme uses a non-diet approach to prevent unduly restrictive eating which can lead to problematic eating behaviours. - MEND 5-7 is based around key principles in health-related behaviour change and behavioural parent training programmes. - Recognising the importance of family involvement for behaviour change, the programme requires a parent or carer to attend all sessions. - Targeted family support information on child nutrition and active play, as well as parenting practices. - The key is integrating these behaviours into everyday life. |
| (Staiano et al., 2016) | - Children were randomly assigned to one of three conditions.   - Copy-Kids eat fruits and vegetables dvd.   - Copy – Kids Brush Teeth DVD   - a no DVD control. - The intervention consisted of two consecutive days of intervention and a final visit one week later. - The first day consisted of watching a DVD either fruits and vegetables or brushing teeth. - The no dvd control group sat quietly for 7.5 minutes. - The F&V video consisted of toddlers interacting and eating a green bell pepper. - The tooth brushing video consisted of children brushing their teeth for the same duration. - Following the dvd children were given two bowls, one containing green bell peppers and the other containing dry cereal. - The children were instructed to eat as much as they would like of either food type. |
| (Steenbock et al., 2019) | - This consists of 5 modules focusing on physical fitness and healthy eating in day care.   - There are three modules that focus on the children,   - One on parental participation   - One on promoting health among daycare staff. - The three modules focusing on children were designed to affect dietary and physical activity habits in 3–6-year-olds. |
| (Suarez-Balcazar et al., 2014) | - Twice monthly education session lasting 30-45 minutes, including   - Identification of fruit and vegetables.   - Importance of fruit and vegetables for health- Age-appropriate highlighting 1 fruit and vegetable per month   - A project: Growing tomatoes in a large sunny window. - These sessions lasted about 30 to 45 minutes and covered the following:   - identification of fruits and vegetables (F=V),   - importance of F=V to health by highlighting one food item each month in an age-appropriate manner,   - a container gardening project in the classroom in which the students learned how to plan tomatoes which were placed by a large sunny window in the classroom.   - At one of the sessions, researchers partnered with an African American rural community farmers co-op to visit with the students and demonstrate farming skills and vegetable crops.   - Team members used handouts with large and colourful pictures, actual fruits and vegetables, and mostly visual material to introduce the F=V. |
| (Tani et al., 2021) | - School promotion of eating vegetables first as part of the “eating vegetables first at meals’ campaign. - Measuring the proportion of children eating vegetables at 133 nursery schools. - Questions include:   - “How often does your child eat vegetable dishes?”     - Response options: almost every week     - Two meals a day     - One meal a day or less - Willingness to eat vegetables as part of a measure of neophobia was recorded.   - “Does your child eat vegetables on his/her own?” Yes/no   - “Is the variety of vegetables that your child can eat increasing?” yes or no.   - “Does your child eat vegetables as the first bite at meals” yes or no   - Proxy for penetration of vegetable eating promotion. |
| (Toossi, 2017) | - Experimental procedure in a field experiment. - During the first week the children were told that they could choose between a fruit cup or a cookie for dessert. - During the next intervention period the children were told again that they could choose between a fruit cup and a cookie cup for dessert but that they would receive a prize for not choosing the former and nothing for choosing the latter. - The final post intervention week mimicked the first week of the experiment. Children also had the option of choosing neither dessert in each period. - After lunch a tray of desserts was set out lined with white napkin and displaying 24 translucent plastic cups containing fruits on the left-hand side and 24 identical cups containing cookies on the right-hand side. - As the children approached the dessert try they were provided the appropriate information for the stage of the experiment. - All of the children were instructed not to throw away their cups until their consumption was recorded. - The consumption was recorded as either a quarter, half, three quarters, all of a cup or none. |
| (Toussaint et al., 2021) | - A Healthy Start programme (AHS). 3 face-to-face meetings of 2 hours each were organised for 8 groups of ECEC teachers. - Each meeting was led by 1 of the coaches and a member of the research staff. - The 3 meetings included theory and practical assignments from the basic national AHS module about a healthy childcare environment and in-depth national AHS modules about Nutrition, Physical Activity and Body weight. - In the first meeting, the teachers reflected on their personal lifestyle outside preschool and the 2015 Dutch dietary guidelines were discussed. - The second meeting focussed on the interaction with children regarding a healthy lifestyle, and the pivotal role of ECEC teachers in setting a healthy example. - Best practices were promoted. For example, ECEC teachers learned not to comfort with food and model healthy eating by eating healthy foods in front of the children during the food breaks at preschool. - Also, they were encouraged to initiate fun games that get the children moving. The third meeting concerned the interaction with parents regarding a healthy lifestyle. - PLAYgrounds for TODdlers programme (PLAYTOD). PLAYTOD was designed to coach ECEC teachers on how to stimulate physical activity (in particular fundamental movement skills) on the playgrounds of preschools. - It focussed on the teachers’ knowledge and practices in order to create a challenging outdoor environment in which young children can practice their motor skills. - A detailed description of the intervention was previously published. In short, 2 face-to-face training sessions of 2 hours each were organised for 4 groups of ECEC teachers. - Each meeting was led by 2 certified PLAYTOD trainers. - The first training session included theory about the importance of (outdoor) physical activity and a basic inviting structure of the playground (with the use of different activity zones) for variation in fundamental movement skills was demonstrated and practiced. - After 2 weeks, ECEC teachers received a coaching on the job session. In the second training session, the activating role of the teachers (prompts) on the playground was practiced and reviewed in more detail. PLAYTOD was derived from the effective PLAY grounds programme for primary schools. - The adherence to the multi-component programmes was determined by attendance records. ECEC teachers who attended at least 2 meetings of AHS and 1 training session of PLAY-TOD received certificates. |
| (Van Stokkom et al., 2018) | - Session typically took less than 30 min, only very few sessions lasted up to 45 mins. - Each session included no more than four children. - Sessions started with oral instructions after which the parent signed the informed consent form and children gave their assent by colouring in a happy smiley face. - If children did not want to participate, they had the option colouring in a sad smiley face. - One child coloured in a sad smiley face and therefore did not participate. - Children were placed in test rooms with no more than two children per test room. - Research assistants helped each child one-on-one and recorded the results. - Parents were seated nearby, but not in direct view of the child and were instructed not to communicate with the child during the test. Serving order of vegetable type was balanced and alternated across sessions, so in one session all children started with cucumber and the next session with green capsicum. - For each vegetable type, children tasted a small portion of each of the seven samples, one by one, using a randomized design across participants. - Vegetable samples (∼15 g per sample) were offered at room temperature in plastic containers (30 ml) covered with a lid until consumption. - During tasting, children were instructed to sip a small amount of water between samples to cleanse their palate. - Children received a tray containing seven samples of the first vegetable (unmodified; low, medium and high sucrose; low, medium and high citric acid). - When children completed the assessment of the first vegetable type, there was a break of at least 1 min. - The procedure was then repeated for the second vegetable. - Acceptance was assessed using a 5-point hedonic facial scale and by preference ranking. - First, children categorised each sample by placing the sample below the appropriate image of a 5-point hedonic facial scale. - The five categories were visualized with smiley faces on an A3 page. - Children's under-standing of the scale was established during the oral instruction basking children to explain the different categories in their own words. - Additionally, the research assistant repeated that the faces represent ‘really yucky’, ‘yucky’, ‘okay’, ‘yummy’ and ‘really yummy’. - Next, the same seven samples were ranked for preference from most (rank number 1) to least liked (rank number 7) |
| (Vandeweghe et al., 2016) | - Mothers and children were invited to the Tasting Lab at Ghent University for participation on any of the 17 days with 16 moments on each day. - In advance, each day was assigned to a specific condition through simple randomization (by throwing some dice). - The Randomized Controlled Design permitted between-group analyses of the different strategies. - Each child was tested individually without the mother's presence by a trained research assistant following a standardized protocol. - To put the child at ease, the experimenter first socialized with him or her for 5 min. - Then, the child was seated at a children’s table and the child's degree of hunger was assessed. - In every condition, the child was offered a small portion (±4 g) of bite-sized steamed or boiled vegetable that he or she dislikes. - The vegetable was unseasoned and was served on a neutral plate. - The way in which the instruction to taste was given differed according to four different strategy conditions: in the neutral instructions condition, which was a control condition, the experimenter asked the child neutrally to taste the vegetable (“If you want, you are allowed to taste”). - In the modelling condition, the tasting behaviours modelled by the experimenter along with the words “Mammoth’s vegetable is delicious! If you want, you are also allowed to taste”. - In the reward condition, the experimenter promised the child a small tangible reward worth approximately 1 dollar (e.g., atopy of choice such as stickers, toy bears, coloured pencils, jumping ropes, bubble blowers, colouring books, toy cars, balls, coloured chalk, paint) if he or she tasted (“If you taste, you can choose a toy from this box!”). - In the encouragement condition, the experimenter verbally encouraged the child to taste (“Come on, you can do it!”). - The sentences were voiced in a positive, child-friendly tone. - Furthermore, in every condition, we made efforts to prevent children from feeling obligated to taste (“You can choose whether you taste or not. I won't be angry if you don't taste”). - An important note that no verbal encouragement was offered in the reward and the modelling conditions. - To control for visual exposure, the child was exposed to the food 1 min before he or she was allowed to taste. - After 1 min during which the child was supplied with the relevant instructions, the experimenter gave the child a spoon and a fork, along with the words: “Now you can taste”. - Each session was videorecorded and lasted approximately 10 min. - Afterwards, the children and the parents were thanked for their cooperation and debriefed by e-mail; each child received a small toy, and the parent(s) were given two information brochures about healthy food. |
| (Vandeweghe et al., 2018) | - Four comparable conditions   - RE + token reward condition,   - RE + social reward condition,   - RNE condition   - control condition - Classes were assigned to conditions using a stratified randomization procedure, based on grade and school (i.e. each condition comprised two classes from a different grade and a different school). - In every condition (except for the control condition), a small portion was offered repeatedly (i.e. 8 times on 8 different days) to each child individually ( ± 4 g) of steamed chicory. - Each time, the child was seated at a small table next to the classroom across the research assistant who provided the instructions. - The way the chicory was offered differed dependent on the allocated condition. In the RE + token reward condition, the child could earn a sticker if he or she tasted. - When the child had collected enough stickers, he or she would receive a toy. - In the RE + social reward condition, the child received social approval when he or she tasted. - This social reward was announced through the experimenters’ verbal encouragement. - In the RNE condition, the experimenter asked the child very neutrally to taste the vegetable. - After tasting, the child had to indicate the degree to which he or she liked the chicory by means of cartoon drawings of facial expressions. - Besides these liking assessments during the tasting trials, we also assessed the willingness to taste (i.e. whether or not the child tasted on each tasting trial). - Each tasting trial lasted approximately 3 min. - The tasting trials begun after playtime (at 10:30 PM) and were finished before noon. - The tasting trials took place twice a week during 4 weeks - A ninth tasting trial was included for children who were absent on a previous tasting trial. - In the control condition, no tasting trials took place. |
| (Vaughn et al., 2021) | - Titles the Healthy Me Healthy We study, designed to increased children’s diet quality and physical activity. - Four - 6-week units. - Teachers hand the posters in the classroom as a reminder of the unit goal. - And a prompt for classroom activities. - Cue cards (n=16) were used to lead 8 a classroom activities. - At home activities were also offered. - Parents were also asked to log activities on an at home activity tracker. - The campaign concluded with celebration events. |
| (von Nordheim et al., 2022) | - At both time points pre and post-test, groups of children were accompanied by one to two familiar nursery teachers into and activity room and instructed to sit down in a viewing area. - In this viewing area the researcher welcomed the children and explained what was going to happen. - The children were given a summary of the ‘Shaun the sheep’ episode before they watched it. - At time 1 the children watched one episode of Shaun the Sheep and then selected healthy foods from the buffet, which they then consumed in an eating area that was set up. - At time 2 the children watched the same shaun the sheep, however it was interrupted by an advertising clip, which was depicting healthy foods in the intervention group and toys in the control group. - This advertising clip was played at the beginning, middle and end of the episode of shaun the sheep. - Once the children had finished viewing they were asked to sit in front of the buffet. The benches were placed so that peer influence was minimised. - The children were advised that they could eat what and how much of anything they wanted and that they could return to the buffet to eat more. - Drinking water was provided at all the tables. - Children were told not to swap or share foods. - Children who were finished were asked once if they wanted any more, if they replied ‘no' they were allowed to return to the nursery so they did not distract the others. |
| (Whiteside-Mansell et al., 2021) | - WISE was specifically developed for preschool and elementary schools serving children from backgrounds impacted by limited resources. - The three key components of WISE was implemented:   - a manualized classroom curriculum,   - educator training   - material and technology to educate parents. - WISE includes Windy, the barn owl mascot, to engage children in the lesson and take the message home. - WISE includes a lesson plan/schedule, suggestions for integration into educational activities (e.g., math), and hands-on recipes. - We provided educators with standardized, hands-on training on best-practice messages and implementation. - WISE focuses on one target food each month in a specific order that begins with typically familiar foods (e.g., apples) and moves to less familiar, in-season foods throughout the school year (e.g., greens). - Carotenoid levels of WISE foods vary from low (e.g., apples) to moderate (e.g., tomatoes) to high (e.g., spinach). - WISE engages families via monthly letters from a farmer and on social media (e.g., Facebook and Pinterest). - Training for preschool educators consists of an interactive session based on adult learning theories. - Changing adult behaviour is thought to require increases in both knowledge and practice. - Adult learners benefit from active rather than passive instruction, appropriate levels of challenge, monitoring and feedback, time to organize and integrate new ideas and concepts, and help generalizing to different contexts. - Training content should be related to participants’ own practice situations, and use learners’ experiences as a point of departure. - Training includes an overview of the roles of educators and parents in child nutrition and exploration of the participant’s food attitudes and beliefs. - Training also includes positive role modelling, food experiences in small groups, appropriate use of the WISE mascot, and positive feeding practices (e.g., reduced pressure). |
| (Williams et al., 2014) | - Program Components. The curriculum includes 10 modules, each with detailed, weekly lesson plans and activities. - RDNs, in consultation with the centre directors, select 6 of the10 modules to use in classrooms and with parents separately. - Some of the most frequently taught modules used for this intervention included   - trying new foods (Food Mood)   - eating a variety of vegetables (Vary Your Veggies);   - eating a variety of fruits (Flavorful Fruit)   - incorporating more healthy dairy products into the diet (Dairylicious)   - eating healthier snacks (Smart Snacking)   - and engaging in physical activity (Fitness Is Fun). - The four less frequently used modules focused on helping children listen to their own body’s   - signs of hunger and fullness(Awesome Appetites)   - safe and healthy food preparation with young children (Cooking with Children)   - identifying, buying, and preparing locally grown fruits and vegetables (Growing Goodness)   - a lesson providing additional ideas for increased physical activity and decreasing television watching at home (Choose Your Fun). - The classes for children last approximately 30 minutes, while the parent classes are 30 to 60 minutes per session. - Each module also includes take-home materials and activities provided in English and in Spanish (when appropriate) that the parents can do with their children to reinforce the messages at home. - Finally, the RDN works with each centre director to identify areas of policy improvement that can enhance nutrition at the centre and teaches at least two classes to the centre’s staff to help them integrate the program’s messages into their classroom activities when the RDN is not there. - Seven of the 11 RDNs who taught the lessons had 3 or more years of experience providing nutrition and health education for children and families in community-based settings. - The RDNs were racially and ethnically diverse. |
| (Willis et al., 2014) | - The 8-week HENRY parent course is delivered by trained facilitator pairs to groups of 8–10 parents. - Each session lasts for 2.5 h, - Each session consisted of ‘Family Time’ where parents and children enjoy a healthy snack and play an active game - Also ‘Parent Time’ where group members explore the programme topics together while the children attend a crèche. - Participants explore a new topic each week (e.g. parenting skills, portion sizes, physical activity and play) through activities that lead to shared understanding and ideas for strategies to support changes. - At the end of each session, group members are encouraged to set individual goals for the week ahead. - Each week, parents build a ‘resource toolkit’ of materials that promote the course's key messages, including a game, portion size guide and story books. |
| (Witt & Dunn, 2012) | - The 17 classrooms were randomly assigned to either group 1 or group 2. - 10 classrooms were assigned to the CMH program and the remaining 7 acted as comparisons. - During the study the comparison classrooms did not incorporate nutrition curriculum into their lesson plans. - For the CMH program classes the Color Me Healthy program was delivered across 6 weeks. - This involved 2 circle time lessons and 1 imaginary trip being taught to the children each week. - The lessons lasted 15-30 minutes. - To evaluate whether there was an increase in consumption of fruits and vegetables as snacks the children were given fruit and vegetable snacks 1 week prior to, 1 week after and 3 months after the program. - The snacks were weighed before and after they were given and the difference was recorded. - The fruit snack consisted of   - 4 purple grapes,   - 4 chunks of pineapple,   - 4 cantaloupe chunks,   - 2 strawberries. - The vegetable snacks consisted of   - 2 cherry tomatoes,   - 2 celery sticks,   - 3 broccoli florets   - 3 baby carrots. - Ranch dressing was given with the vegetables. - Each snack also contained 2 Ritz crackers and a large graham cracker. - Fruits were served on Tuesdays and Wednesdays, Vegetables on Thursdays and Fridays. |
| (Yoong et al., 2020) | - Web-based menu-planning program (“feedAustralia”): The program undertook automated assessments on meals and snacks entered by menu planners and provided realtime feedback on the number of servings of each of the core food groups and discretionary foods - The feedback also outlined whether the menu was compliant with that recommended by sector-specific nutrition guidelines. - The calculation of compliance was underpinned by a national nutrition database containing food group and nutrient information developed by the research team. - Provision of educational resources: Where menus were not compliant, the program automatically provided childcare centres with suggestions and recipes on how to modify the menus to meet guidelines. - The online menu-planning tool (“feedAustralia”) also included >200 healthy recipes that met the guidelines for inclusion in childcare menus as well as complete 1-wk sample menus that were compliant with guidelines. - Reminders: Childcare centres were also prompted fortnightly in the main CCMS to make changes to their menu to increase compliance if noncompliant, or if they had an incomplete menu in the program. - Training, educational resources, and managerial support: A health promotion officer with experience using the program conducted a 3-h training session with the supervisor and menu planner within each childcare service. - The training consisted of updating centres with sector-specific nutrition guidelines, the main features of the online menu-planning program and how to use them, and supporting the service to make changes to their menu. - To generate service-level support for use of the program, the health promotion officer also undertook action planning to ensure that allocated time and resources were provided to the menu planner to access the program. - Centres were also provided with a portable tablet to facilitate access to the program and recipes during food preparation processes. - Ongoing support: Ongoing support was provided over 2–4 phone calls to childcare service cooks. - The purpose of these calls was to provide technical support with using the program and overcome any reported barriers to using the program. - The number of phone calls provided to each service was tailored depending on engagement with the program and menu compliance as assessed via analytics data collected via the program. - Centres could also seek support via an online “helpdesk” feature available within the program. - The helpdesk feature, however, was poorly accessed with no centre accessing this. |
| (Zeinstra et al., 2017) | - A video film was created specifically for this study with the help of two Dutch children’s TV idols (adults), called Ernst and Bobbie - In the 4-min video, they are enthusiastic about vegetables in general, and about carrots in particular. - While they eat carrots enthusiastically, the story illustrates that carrots will make you strong and superfast. - The film includes a catchy song about vegetables. - Because children may associate healthy with distaste no explicit emphasis was given to the healthfulness of vegetables. - The same video was shown during the sessions, as repetition is well suited for this age group. - The intervention took place in the children’s own classroom. - The children were sitting in their normal class situation: small groups with approximately six children per table. - The eating sessions and the choice tests (separate days) took place on Tuesdays and Thursdays during the children’s habitual morning snack time (10:30) and lasted both about 18 min. - During all sessions, the children received a 200 ml fruit drink (78 kcal) and one container with 100 g of vegetables (carrots during S1–S8 and their own chosen vegetable during the choice test). - They were instructed to eat from their own container and not to share food with each other. - A researcher was present in the classroom during the sessions to help distribute the products and to observe the procedures and the children’s reactions, but he/she remained in the background. - All teachers participated in an informative workshop prior to the start of the study in which the study procedures and guidelines were explained |
